# Transient *DUX4* expression in human embryonic stem cells induces blastomere-like expression program that is marked by SLC34A2

**DOI:** 10.1016/j.stemcr.2022.06.002

**Published:** 2022-06-30

**Authors:** Masahito Yoshihara, Ida Kirjanov, Sonja Nykänen, Joonas Sokka, Jere Weltner, Karolina Lundin, Lisa Gawriyski, Eeva-Mari Jouhilahti, Markku Varjosalo, Mari H. Tervaniemi, Timo Otonkoski, Ras Trokovic, Shintaro Katayama, Sanna Vuoristo, Juha Kere

**Affiliations:** 1 Department of Biosciences and Nutrition, Karolinska Institutet, Stockholm, Sweden; 2 Institute for Advanced Academic Research, Chiba University, Chiba, Japan; 3 Department of Artificial Intelligence Medicine, Graduate School of Medicine, Chiba University, Chiba, Japan; 4 Department of Obstetrics and Gynecology, University of Helsinki, Helsinki, Finland; 5 Research Programs Unit, Stem Cells and Metabolism and Biomedicum Stem Cell Centre, Faculty of Medicine, University of Helsinki, Helsinki, Finland; 6 Department of Clinical Science, Intervention and Technology, Karolinska Institutet, Stockholm, Sweden; 7 Division of Obstetrics and Gynecology, Karolinska University Hospital, Stockholm, Sweden; 8 Institute of Biotechnology, University of Helsinki, Helsinki, Finland; 9 Folkhälsan Research Center, Helsinki, Finland; 10 Children’s Hospital, Helsinki University Central Hospital, University of Helsinki, Helsinki, Finland

**Keywords:** DUX4, embryonic genome activation, embryonic stem cells, human embryo, blastomere, reprogramming, SLC34A2, NaPi2b

## Abstract

Embryonic genome activation (EGA) is critical for embryonic development. However, our understanding of the regulatory mechanisms of human EGA is still incomplete. Human embryonic stem cells (hESCs) are an established model for studying developmental processes, but they resemble epiblast and are sub-optimal for modeling EGA. DUX4 regulates human EGA by inducing cleavage-stage-specific genes, while it also induces cell death. We report here that a short-pulsed expression of *DUX4* in primed hESCs activates an EGA-like gene expression program in up to 17% of the cells, retaining cell viability. These *DUX4*-induced cells resembled eight-cell stage blastomeres and were named induced blastomere-like (iBM) cells. The iBM cells showed marked reduction of POU5F1 protein, as previously observed in mouse two-cell-like cells. Finally, the iBM cells were successfully enriched using an antibody against NaPi2b (SLC34A2), which is expressed in human blastomeres. The iBM cells provide an improved model system to study human EGA transcriptome.

## Highlights

• Transient *DUX4* induction activates EGA genes in hESCs with little cytotoxicity
• Up to 17% of the cells showed high similarity with the eight-cell stage blastomeres
• These cells showed marked reduction of POU5F1 protein, as observed in mouse 2CLCs
• These cells were enriched with FACS using an antibody against NaPi2b (SLC34A2)

## Graphical abstract

**Figure:**
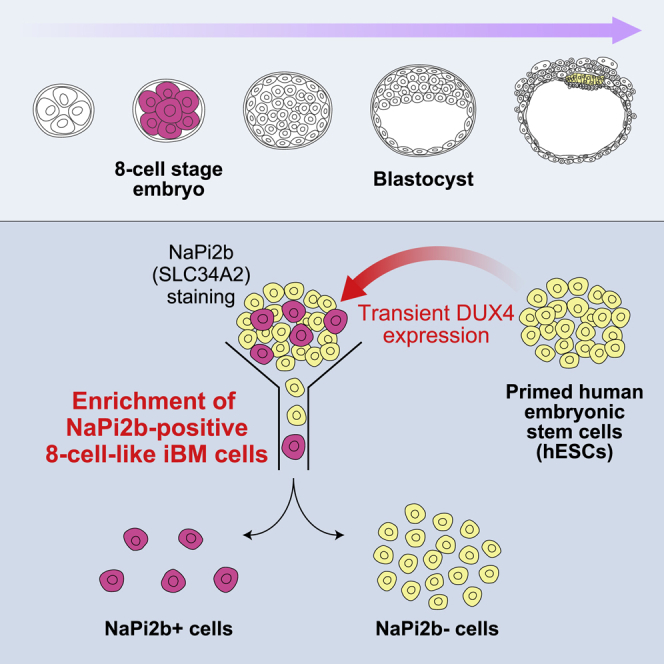

## Teaser

In this article, Yoshihara and colleagues show that transient *DUX4* induction reprograms human ESCs into an eight-cell-like transcriptional state with little cytotoxicity. These cells, named iBM cells, were enriched with FACS using an antibody against NaPi2b (SLC34A2). The iBM cells provide a new *in vitro* model to study the mechanisms of human EGA transcriptional program.

## Introduction

Embryonic genome activation (EGA) is a crucial process for the normal development of preimplantation embryos, where zygotic genes start to be transcribed. The timing of EGA varies among species, at the two-cell stage in mouse and at the four- to eight-cell stage in human (Braude et al., 1988; Jukam et al., 2017; Töhönen et al., 2015). Recent technological advances have enabled us to study transcriptional dynamics of EGA during the embryogenesis (Petropoulos et al., 2016; Töhönen et al., 2015; Yan et al., 2013). However, the detailed regulatory mechanisms of EGA have yet to be elucidated, especially in human, due to the limited availability of samples and ethical concerns. Therefore, there is a great need for an *in vitro* model system to investigate human EGA transcriptional program.

*DUX4*, a double homeobox transcription factor, is transiently expressed in the human cleavage stage embryo (Töhönen et al., 2017) and regulates human EGA by inducing transcription of cleavage-stage-specific genes and repetitive elements (De Iaco et al., 2017; Hendrickson et al., 2017; Vuoristo et al., 2022; Whiddon et al., 2017). However, *DUX4* overexpression in somatic cells leads to cell death both *in vitro* and *in vivo* (Bosnakovski et al., 2008; Kowaljow et al., 2007; Rickard et al., 2015; Wallace et al., 2011). A recent study reported that transient *DUX4* expression in human myoblasts activates its target genes with little cytotoxicity by inducing histone variants H3.X/Y, which contribute to the perdurance of DUX4 target gene expression with the open chromatin conformation (Resnick et al., 2019).

A rare cell population of mouse embryonic stem cells (mESCs) exhibit two-cell-like signatures (Macfarlan et al., 2012), with the reduced expression of pluripotency markers, such as POU5F1, SOX2, and NANOG, and increased expression of targets of DUX, the mouse ortholog of human DUX4 (Rodriguez-Terrones et al., 2018). These two-cell-like cells (2CLCs) have been used as an *in vitro* model to study mouse EGA (De Iaco et al., 2019; Genet and Torres-Padilla, 2020), and they spontaneously transit toward the pluripotent state under the culture conditions optimal for mESCs (Macfarlan et al., 2012). Recent studies have revealed that mouse 2CLCs can be induced by *Dux* expression (De Iaco et al., 2017; Fu et al., 2019; Hendrickson et al., 2017; Yang et al., 2020). These findings prompted us to examine whether human ESCs (hESCs) could be converted to an early embryonic-like state by transient *DUX4* expression.

Here, we show that short induction of *DUX4* in primed hESCs activates EGA genes with little toxicity. We further identified a cell population, named induced blastomere-like (iBM) cells, that showed similar expression profile with eight-cell stage blastomeres. These iBM cells were enriched with fluorescence-activated cell sorting (FACS) using an antibody against a cell surface antigen, NaPi2b (SLC34A2), expressed in preimplantation embryos. The iBM cells provide a new *in vitro* model to study the mechanisms of human EGA.

## Results

### Transient *DUX4* induction activates EGA genes in hESCs with little cytotoxicity

**Figure 1:**
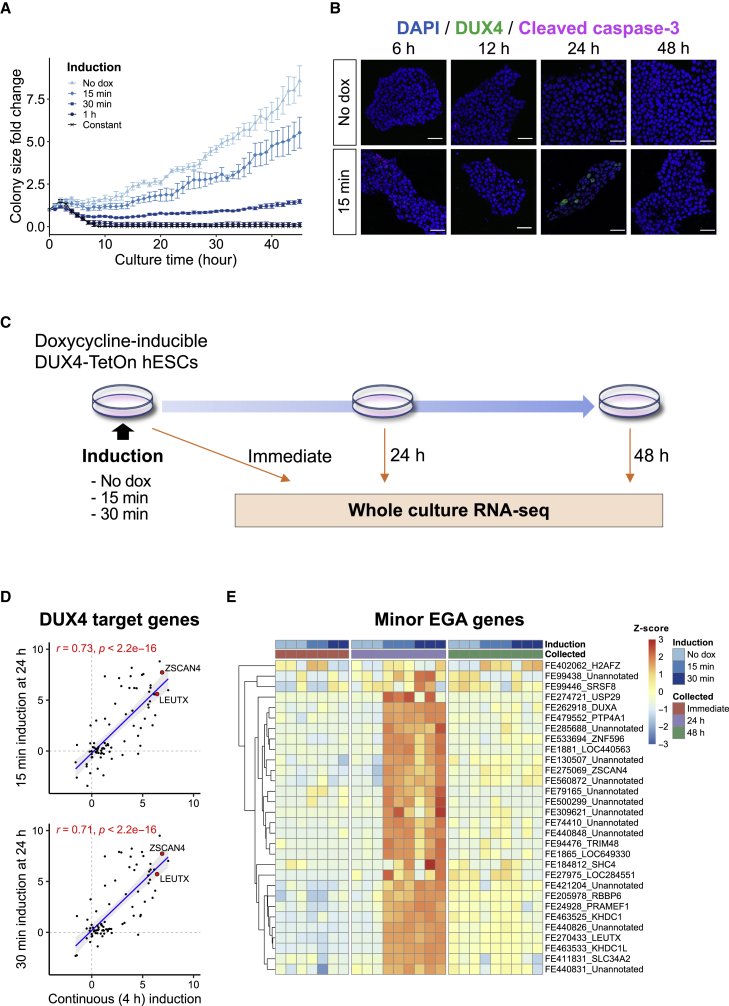
Transient *DUX4* induction activates EGA genes with little cellular toxicity (A) Growth rate of DUX4-TetOn hESCs after varied times of doxycycline induction. Colony size fold change was calculated based on the starting time point (0 h). Data represent mean ± SEM (n = 5 replicates from different culture wells). (B) Immunocytochemical detection of DUX4 and cleaved caspase-3 in DUX4-TetOn hESCs without induction (top) and after 15 min of induction (bottom). DAPI (blue) was used as nuclear counterstain. Scale bars, 20 μm. (C) Schematic representation of the whole culture RNA-seq on DUX4-TetOn hESCs with varied induction times. (D) Transcriptional changes of 80 DUX4 target genes expressed in early human embryo after continuous *DUX4* induction (x axis) and transient *DUX4* induction (y axis). Axes show the log_2_ fold expression changes over no *DUX4* induction. The correlation coefficients (r) and p values were calculated using a two-sided Spearman’s correlation test. The linear regression line (blue) and 95% confidence interval (gray shaded) are shown. See also Table S1. (E) Heatmap showing the expression of minor EGA genes at TFE level. Of the 32 minor EGA genes, 30 genes that were expressed are shown. TFE, transcript far 5′ ends. See also Figure S1.

To test whether hESCs continue to proliferate after the transient *DUX4* induction, we first measured the expansion of the doxycycline-inducible DUX4-TetOn hESCs after various durations of doxycycline exposure (15 min, 30 min, 1 h, and constant). While doxycycline treatment for 1 h or longer caused vast cell death after prolonged culture, 15-min treatment resulted in a temporary decrease in growth rate, returning to a similar level with that of the cells without induction (Figures 1A and S1A). Moreover, only a small number of apoptotic cells were detected after 15 min of treatment, at levels similar to cells without an induction. Importantly, DUX4-positive cells were not positive for cleaved caspase-3, implying that transient *DUX4* expression did not induce apoptosis in hESCs (Figure 1B).

Next, to determine whether a transient *DUX4* induction is sufficient to activate its target genes, DUX4-TetOn hESCs were exposed to doxycycline for 15 or 30 min and subjected to RNA sequencing (RNA-seq) (Figure 1C). Principal-component analysis (PCA) demonstrated that cells collected at 24 h after either 15- or 30-min treatment were clearly separated from other samples along PC2, which was highly contributed by *LEUTX* and *ZSCAN4*, suggesting that short induction had the largest effect at 24 h post-induction (Figure S1B). Notably, cells at 24 h after 15 and 30 min of treatment showed highly similar expression profiles (r = 0.96; Spearman correlation) (Figure S1C). The expression changes of DUX4 target genes (Table S1; Resnick et al., 2019) were similar between continuously (4 h) treated cells (Vuoristo et al., 2022) and pulsed cells at 24 h post-treatment, suggesting that the 15- and 30-min pulses were sufficient to activate DUX4 target genes (Figures 1D and S1D). Furthermore, cleavage-stage-specific repetitive elements, such as MLT2A1, MLT2A2, and HERVL, activated by DUX4 (Geng et al., 2012; Hendrickson et al., 2017; Young et al., 2013), were significantly upregulated at 24 h after pulse (Figure S1E).

We previously investigated the dynamics of the human preimplantation transcriptome by single-cell tagged reverse transcriptase (STRT) RNA-seq quantifying the transcript far 5′ ends (TFEs) and identified 32 TFEs upregulated at the four-cell stage as minor EGA genes (Töhönen et al., 2015), most of which should be regulated by DUX4 (De Iaco et al., 2017). We found that 30 of them were expressed in the *DUX4*-induced hESCs, and most of them showed the highest expression at 24 h after short induction but again reduced at 48 h (Figure 1E). These observations suggest that only a 15-min induction of *DUX4* might be able to convert hESC transcriptome into a blastomere-like state.

### Transient *DUX4* induction reprograms hESCs into an eight-cell-like transcriptional state

**Figure 2:**
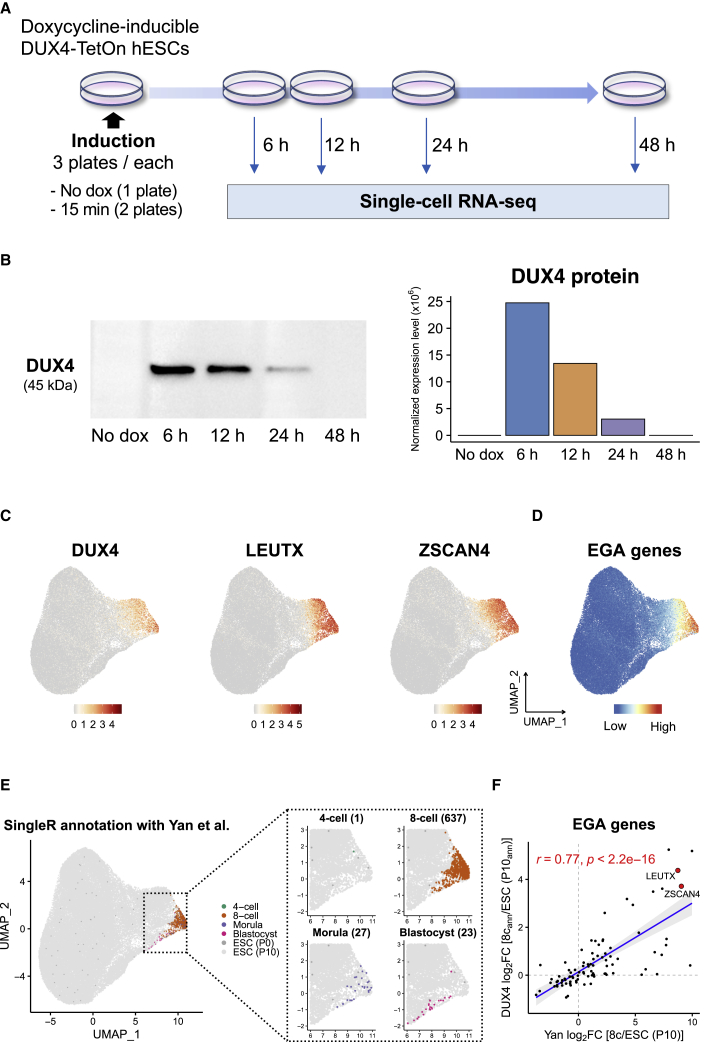
Time series single-cell transcriptomic profiling of *DUX4*-pulsed hESCs (A) Schematic representation of the scRNA-seq experiments. (B) Western blot analysis (left) and quantification (right) of DUX4 protein expression levels after 15 min of *DUX4* induction by collected time points. Expression levels were normalized to total protein. (C) Expression of *DUX4*, *LEUTX*, and *ZSCAN4* projected onto the UMAP plot. (D) Expression score of EGA genes projected onto the UMAP plot. See also Table S1. (E) Cell type annotation with human preimplantation embryos and hESCs using SingleR. The right four panels show the magnified plots of the cells annotated as early embryonic stage cells. Numbers in parentheses indicate the number of the annotated cells. P0, passage 0; P10, passage 10. (F) Transcriptional changes of 92 expressed EGA genes in actual eight-cell stage cells (x axis) and *DUX4*-pulsed hESCs annotated as eight-cell stage cells (y axis) compared with hESCs. Axes show the log_2_ fold expression changes over hESCs (P10; x axis) or cells annotated as hESCs (P10; y axis). The correlation coefficients (r) and p values were calculated using a two-sided Spearman’s correlation test. The linear regression line (blue) and 95% confidence interval (gray shaded) are shown. 8c_ann_, cells annotated as eight-cell stage cells; ESC (P10_ann_), cells annotated as ESC (P10). See also Table S1 and Figure S2.

To examine whether early embryonic-like cells arise after transient *DUX4* induction, we performed time-series single-cell RNA-seq (scRNA-seq) on the DUX4-TetOn hESCs treated with doxycycline for 15 min (Figure 2A). We added two earlier time points before 24 h because the expression of DUX4 target genes might peak earlier, given their temporal expression in the embryo (De Iaco et al., 2017; Hendrickson et al., 2017). Here, we confirmed that DUX4 protein was highly expressed already at 6 h after induction but rapidly reduced and disappeared at 48 h (Figure 2B), which mimics its dynamics in the embryo (Vuoristo et al., 2022). After filtering out low-quality cells, 65,460 cells were retained for downstream analyses (Table S2). Dimensionality reduction by uniform manifold approximation and projection (UMAP) demonstrated that untreated (no dox) cells formed one main cluster, which clustered with many of the *DUX4*-pulsed cells (Figure S2A), in line with the low reprogramming efficiencies of human induced pluripotent stem cells (Schlaeger et al., 2015). *DUX4* and its target genes were highly expressed in the rightmost *DUX4*-pulsed cells along the first UMAP dimension (Figures 2C and S2B). Approximately 30%–40% of cells expressed *DUX4* and its target genes at 6 and 12 h, whereas only ∼5% of cells expressed them at 24 h, in line with the quantitative real-time PCR of whole culture cells (Figure S2C). EGA genes were specifically expressed in the rightmost cluster (Figure 2D; Table S1).

To address the similarity of these cells with early human embryonic cells, we annotated the cells against the scRNA-seq data of preimplantation embryos and hESCs (Yan et al., 2013). Altogether, 637 cells were annotated as eight-cell stage cells (Figure 2E). Of these, 544 cells were collected at 12 h after induction. This indicates that 6.6% of the *DUX4*-pulsed cells (8,268 cells) were converted to a state that transcriptionally resembled eight-cell stage embryo 12 h after induction. Transcriptional changes of the EGA genes in these eight-cell-like cells correlated highly with those in eight-cell stage blastomeres (r = 0.77; Spearman correlation) (Figure 2F). Transcriptional changes of all the expressed genes were less correlated (r = 0.5; Figure S2D, left), most likely reflecting the remaining maternal transcripts that are present in the eight-cell stage blastomeres, but not activated by *DUX4* induction in hESCs. In support of this, among the genes highly expressed in eight-cell stage blastomeres (log_2_ FC > 5 over ESCs), the genes activated by *DUX4* induction were those most highly expressed at eight-cell stage, while the genes not activated by *DUX4* induction were highly expressed in oocytes and zygotes (Figure S2D, right). Of note, the cells annotated as four-cell, eight-cell, or morula were predominant at 12 h, whereas the cells annotated as blastocyst were predominant at 24 and 48 h (Figure S2E). These findings suggest that the transient *DUX4*-pulsed cells might recapitulate the transcriptional dynamics of EGA genes of early human embryo.

### Cell-state transition dynamics after transient *DUX4* induction

**Figure 3:**
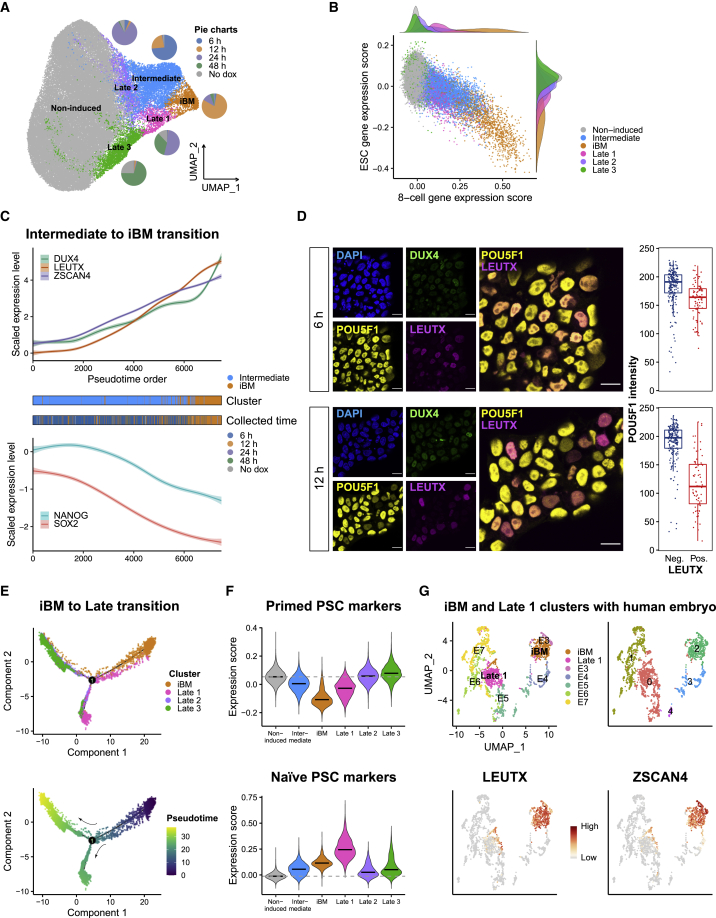
Clustering and trajectory analysis of *DUX4*-pulsed hESCs (A) Clustering analysis of *DUX4*-pulsed hESCs with assigned cluster names. Pie charts represent the proportion of collected time points across the clusters. (B) Expression scores of eight-cell (x axis) and ESC (y axis) of each cell, colored by clusters. Distribution of cells of each cluster is shown at the top and right. See also Table S1. (C) Expression changes of *DUX4* and its target genes (top) and pluripotency marker genes (bottom) of single cells from the intermediate and the iBM clusters along the pseudotime. Middle panels show the cluster (above) and collected time point (below) of each single cell. (D) Immunocytochemical detection of DUX4, LEUTX, and POU5F1 in *DUX4*-pulsed hESCs at 6 h (top) and 12 h (bottom). DAPI (blue) was used as nuclear counterstain. Scale bars, 20 μm. Mean fluorescence intensity of POU5F1 per nuclei in LEUTX-negative (Neg.) and positive (Pos.) cells is shown at the right (n = 293 for 6 h and 259 for 12 h; n denotes the number of analyzed nuclei). (E) Trajectory reconstruction of single cells from the iBM and late clusters: pre-branch (before bifurcation), late 1 fate, and late 2/3 fate (after bifurcation). Top panel is colored by cluster, and bottom is colored by pseudotime. (F) Expression scores of primed and naive PSC marker genes in each cluster. The horizontal black bars in violin plots indicate the median score per cluster, and the horizontal gray dotted line indicates the median score in the non-induced cluster. (G) Integration of iBM and late 1 cluster cells with the human embryo (Petropoulos et al., 2016) projected onto the UMAP plot. iBM and late 1 cluster cells were downsampled to 400 cells per cluster. Colored by original cell identity (top left) and cluster annotation (top right) is shown. Expression levels of *LEUTX* and *ZSCAN4* in each single cell are shown at the bottom. See also Figure S3.

To further characterize the *DUX4*-pulsed cells, we assigned them to six clusters by unsupervised clustering. Based on the proportion of the collected time points and cell type annotations, we named the clusters as follows: non-induced, intermediate, iBM, and late 1, 2, and 3 (Figure 3A). The intermediate cell cluster consisted primarily of 6-h sample cells, the iBM cluster of 12-h sample cells, and the late clusters of 24- and 48-h sample cells. Hierarchical clustering of these six clusters demonstrated that the iBM cluster showed a unique expression profile, whereas the late 2/3 clusters shared similar profile with the non-induced cluster (Figure S3A). The majority of the iBM-cluster-specific genes (Table S3) were most highly expressed at eight-cell stage and downregulated in blastocyst (Figure S3B). The intermediate and late 1 clusters moderately expressed these genes with different patterns. Although none of these genes were expressed in the non-induced cluster, *CCNA1* and *ALPG* were expressed in the late 2/3 clusters. The LEUTX target genes (Table S1; L.G., E.-M.J., M.Y., Fei Liangru, J.W., Tomi T. Airenne, R.T., Shruti Bhagat, M.H.T., Yasuhiro Murakawa, Kari Salokas, Xiaonan Liu, Sini Miettinen, S.V., Thomas R. Bürglin, Biswajyoti Sahu, T.O., Mark S. Johnson, S.K., M.V., J.K., unpublished data) were expressed higher in the late 2/3 clusters than in the non-induced cluster (Figure S3C). As *LEUTX* expression peaked in the iBM cluster (Figure S3B), the late 2/3 clusters were likely derived from the iBM cells, distinguishing them from the non-induced cluster.

To estimate the reprogramming state changes from hESCs to iBM cells, we calculated the eight-cell and ESC gene expression scores in each cell, based on our scRNA-seq data of eight-cell stage cells and hESCs (Table S1; Jouhilahti et al., 2016). As expected, the cells in the non-induced cluster showed a high ESC score with a low eight-cell score, whereas the cells in the iBM cluster showed the lowest ESC score with the highest eight-cell score (Figure 3B). Cells in the intermediate cluster located between these two, suggesting that these cells were in the midst of the transcriptional reprogramming process. Cells in the late clusters showed higher ESC scores with lower eight-cell scores, suggesting that the iBM cells proceeded toward the ESC state.

To dissect the reprogramming process to the iBM cells via intermediate cells, we performed a pseudotime trajectory analysis on the 7,478 cells from these two clusters. The pseudotime order was consistent with the actual collected time points, with the cells collected at 6 h being earlier and 12 h later (Figure 3C). The eight-cell and ESC gene expression scores showed an inverse changing pattern along the pseudotime (Figure S3D). Expression of *DUX4* and its targets increased along the pseudotime, whereas that of pluripotency marker genes, such as *SOX2* and *NANOG*, decreased (Figures 3C and S3E; Table S4). These pluripotency marker genes are lowly expressed in cleavage-stage human embryos (Töhönen et al., 2015). DUX4 and LEUTX proteins were not detected in the untreated cells but were positive at 6 and 12 h (Figures 3D and S3F). Although *POU5F1* transcript did not significantly decrease, LEUTX-positive cells showed remarkably reduced POU5F1 staining, especially at 12 h (Figure 3D), as observed in mouse 2CLCs (Hendrickson et al., 2017; Macfarlan et al., 2012; Rodriguez-Terrones et al., 2018).

Next, to monitor the expression changes after the iBM stage, a pseudotime analysis was conducted on the 8,101 cells from the iBM and three late clusters. Cells from the iBM cluster bifurcated into two diverse branches, late 1 and late 2/3 (Figure 3E). Most of the naive pluripotent stem cell (PSC) markers (Liu et al., 2020) were upregulated along the pseudotime progression in the late 1 lineage, whereas primed PSC markers were highly expressed in the late 2/3 lineage (Figures 3F and S3G). We further directly compared the transcriptome of these cells with that of naive and primed hESCs (Messmer et al., 2019) and found that late 1 cluster cells clustered together with naive hESCs, whereas late 2/3 cluster cells clustered with primed hESCs (Figure S3H). Since naive PSCs have been described to have a similar expression profile as preimplantation epiblast (Liu et al., 2017), late 1 cluster cells were suggested to have some similarity to preimplantation embryos. To investigate the similarity of these cells to human embryos, we integrated our scRNA-seq data with that of human preimplantation embryos (Petropoulos et al., 2016). Of note, the iBM cells clustered together with eight-cell stage embryos (E3) showing similar expression levels of *LEUTX* and *ZSCAN4* (Figure 3G). Late 1 cluster cells clustered with early blastocysts (E5) (Figures 3G and S3I), whereas late 2/3 cluster cells clustered independently from the preimplantation embryos (Figure S3I). These results suggest that a majority of the iBM cells reverted to their original primed hESC state, but a subpopulation of the cells might mimic the transcriptional transition from morula to blastocyst in embryo.

### Viable iBM cells can be enriched with an anti-NaPi2b antibody

**Figure 4:**
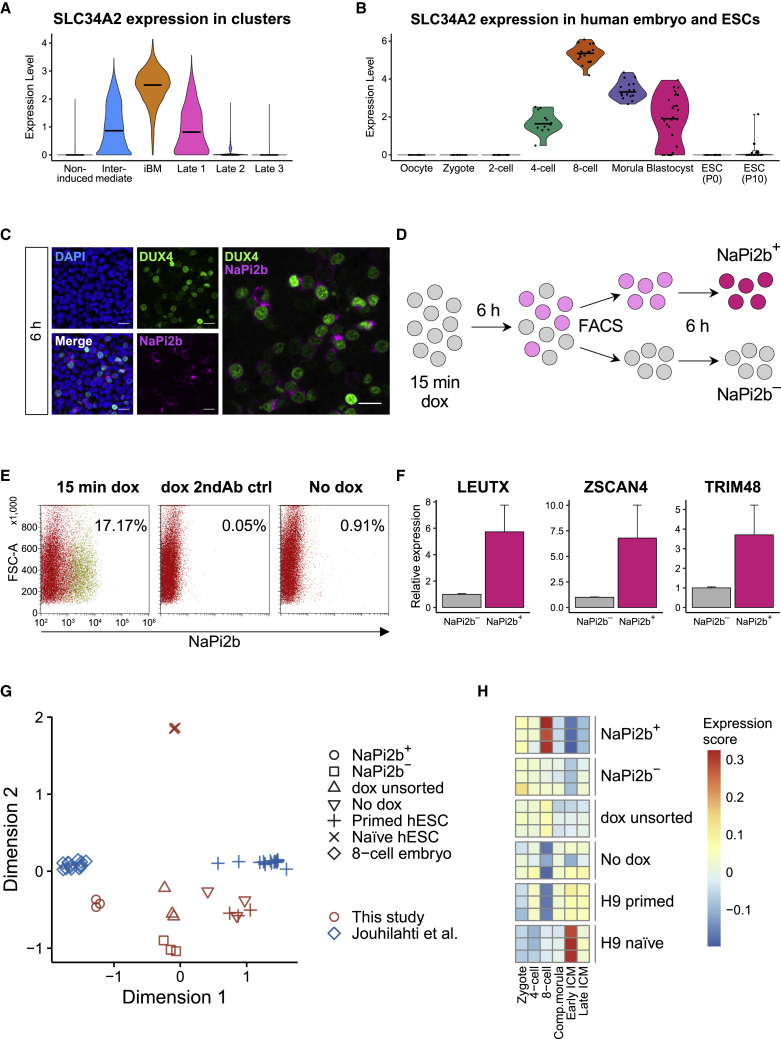
Enrichment of viable iBM cells with an anti-NaPi2b antibody (A) *SLC34A2* expression in *DUX4*-pulsed hESCs in each cluster. Expression levels are shown as log-normalized UMI counts. (B) *SLC34A2* expression in human preimplantation embryo and ESCs (Yan et al., 2013). Expression levels are shown as log fragments per kilobase of transcript per million mapped reads (FPKM) values. The horizontal bars in each violin plot indicate the median expression level per cluster (A) or stage (B). (C) Immunocytochemical detection of DUX4 and NaPi2b in *DUX4*-pulsed hESCs at 6 h. DAPI (blue) was used as nuclear counterstain. Scale bars, 20 μm. (D) Schematic illustration of the cell-sorting procedure. (E) Flow cytometric analysis showing the proportion of NaPi2b^+^ cells (yellow dots). Representative data from two independent experiments are shown. FSC, forward scatter; 2ndAb ctrl, secondary antibody control. (F) DUX4 target gene expression in NaPi2b^+^ and NaPi2b^−^ cells cultured for 6 h after cell sorting. Error bars represent the SEM of two independent experiments. (G) Multidimensional scaling analysis integrating the sorted NaPi2b^+^ and NaPi2b^−^ cells, unsorted *DUX4*-pulsed hESCs (dox unsorted), no induction (no dox), and H9 primed and naive ESCs (n = 3 independent experiments), with the single-cell RNA-seq data of HS980 primed ESCs (n = 16 cells) and eight-cell stage embryo (n = 13 cells) from Jouhilahti et al. (2016). (H) Heatmap showing the expression scores of stage-specific genes. Comp.morula, compacted morula; ICM, inner cell mass. See also Figure S4.

Given that a subset of the *DUX4*-pulsed hESCs were classified as iBM cells, a practical method for the iBM cell enrichment is needed. We searched for a potential cell surface antigen specifically expressed in the iBM cluster (Table S3) and identified *SLC34A2*, encoding the sodium-dependent phosphate transporter NaPi2b (Figure 4A). *SLC34A2* is also one of the DUX4 target genes (Hendrickson et al., 2017) and an EGA gene that is highly upregulated at the four- and eight-cell stage embryos (Figure 1E; Töhönen et al., 2015) but rarely expressed in hESCs (Figure 4B). Mouse 2CLCs also highly express *Slc34a2* (Hendrickson et al., 2017). Similar to other DUX4 target genes, *SLC34A2* was highly expressed at 6 and 12 h after induction (Figure S4A). We confirmed its expression on *DUX4*-pulsed hESCs at protein level using an anti-NaPi2b monoclonal antibody MX35, which recognizes its extracellular domain (Yin et al., 2008). MX35 specifically stained the cell surface of a subset of the *DUX4*-pulsed hESCs, already at 6 h after induction (Figures 4C and S4B).

Finally, we enriched the NaPi2b^+^ cells by FACS at 6 h post-doxycycline treatment, and given that the iBM cells were most enriched at 12 h post-treatment, we plated the sorted cells for an additional 6 h culture (Figure 4D). The proportion of the NaPi2b^+^ cells in the *DUX4*-pulsed hESCs was up to 17% (in two independent experiments; Figures 4E and S4C). The sorted NaPi2b^+^ cells expressed higher levels of DUX4 target genes than the NaPi2b^−^ cells (Figure 4F). We further characterized these cells by RNA-seq, which distinguished NaPi2b^+^ cells from unsorted *DUX4*-pulsed hESCs or NaPi2b^−^ cells (Figure S4D). The NaPi2b^+^ cells showed more similar expression profile to that of eight-cell stage blastomeres (Figure 4G). Expression levels of eight-cell stage-specific genes (Stirparo et al., 2018) in the NaPi2b^+^ cells were much higher than in the unsorted *DUX4*-pulsed hESCs and NaPi2b^−^ cells (Figures 4H and S4E). Moreover, annexin V staining 6 h post-sorting showed that apoptotic rate between NaPi2b^+^ and NaPi2b^−^ cells was comparable, although slightly higher proportion of the NaPi2b^−^ cells seemed to have attached (Figure S4F). These observations indicate that NaPi2b can be used as a marker to enrich the iBM cells by FACS.

## Discussion

We describe here the transcriptional reprogramming of primed hESCs into iBM cells by transient *DUX4* induction. Our data suggest that hESCs tolerate a short-term DUX4 activation with continued proliferation and without increased apoptosis. Although several studies have investigated DUX4-mediated cytotoxicity (Bosnakovski et al., 2008; Geng et al., 2012; Rickard et al., 2015; Shadle et al., 2017; Wallace et al., 2011), its mechanism is not fully understood yet. Given that DUX4 regulates human EGA genes, it remains unclear how its toxic effect is avoided in embryos. The balance between cytotoxicity and cell survival after *DUX4* induction deserves further studies.

The iBM cells share several features with mouse 2CLCs, which have been used as a model to study totipotency (Genet and Torres-Padilla, 2020). Both showed significant downregulation of *NANOG* and *SOX2* transcripts. POU5F1 protein was reduced in both cell types, although its expression was not significantly affected at transcriptional level (Fu et al., 2020; Hendrickson et al., 2017; Macfarlan et al., 2012; Rodriguez-Terrones et al., 2018). Since the late 1 cluster cells that were likely derived from iBM cells resembled early blastocyst cells, the iBM cells may provide another model with broad differentiation potential.

Finally, the iBM cells were marked by the expression of *SLC34A2*, encoding NaPi2b. Importantly, NaPi2b^+^ cells showed higher similarity with the eight-cell stage blastomeres than other PSC types. Moreover, as an endogenous extracellular epitope, NaPi2b staining allows enrichment of the iBM cells without the need for a transgenic reporter construct. We envision that NaPi2b may be of use for isolating and culturing human eight-cell-like cells that were recently discovered among naive hESCs (Taubenschmid-Stowers et al., 2022).

The iBM cells can become a powerful tool to study the roles of specific genes in the context of the EGA, without the need for early human embryos that is ethically questioned and that are available in limited numbers where allowed. We envision further experiments where single EGA-associated genes can be inactivated by gene editing in hESCs and subsequently induced toward iBM using methods described here. Comparison of such cells to iBM cells by scRNA-seq may illuminate each gene’s functional role and possible redundancies in the EGA transcriptome.

There are some limitations in our study. Our transcriptomic comparison with cleavage-stage embryos showed that EGA genes were efficiently activated by a transient *DUX4* induction. However, many oocyte-specific genes, which remain in the eight-cell stage blastomeres, were absent. Therefore, the iBM cells do not completely mimic the transcriptome of the embryonic blastomere cells. The relevance of these oocyte-specific factors for modeling early embryo behavior with stem cells remains to be determined. Our time-series analysis demonstrated that the iBM cell transcriptome reverted to the original ESC state within 48 h after *DUX4* induction, suggesting that the iBM cells could not be maintained under the culture condition optimal for primed hESCs. Identification of the critical signaling pathways that drive the differentiation of the iBM cells would allow further optimization of the conditions aiming at stable iBM cell cultures. A recent study succeeded in converting human PSCs into eight-cell-like cells with a combination of chemical treatments (Mazid et al., 2022). Another study established stable totipotent-like stem cells from mESCs by chemical induction (Yang et al., 2022). These reported chemicals might be useful for the optimization of the prolonged culture of the iBM cells. Feasibility of the iBM cells as embryo model requires further functional validation, such as directed differentiation assays.

## Experimental procedures

Additional methods and more detailed descriptions of STRT RNA-seq and scRNA-seq can be found in the supplemental experimental procedures.

### Cell culture

DUX4-TetOn hESCs (Vuoristo et al., 2022) were maintained on hESC-qualified Geltrex (Thermo Fisher Scientific) coated tissue culture dishes in Essential 8 culture medium (Thermo Fisher Scientific) in 5% CO_2_ at 37°C. The cells were passaged every 3–5 days after a 3-min incubation with 0.5 mM EDTA (Thermo Fisher Scientific). For the cell growth assays, the cells were imaged with the Incucyte S3 analysis system (Sartorius). Naive H9 hESCs, which had been previously converted from primed to naive stem cell stage using the NaïveCult Induction kit (STEMCELL Technologies), were cultured on irradiated mouse embryonic fibroblast (MEF) feeders (Gibco) in the NaïveCult Expansion Medium (STEMCELL Technologies) in 5% O_2_/5% CO_2_ at 37°C. Naive hESCs were dissociated with TrypLE Express (Thermo Fisher Scientific) every 3–5 days and re-plated on MEF feeders, prepared a day before hESC seeding. The cell culture medium was supplemented with 10 μM ROCKi Y-27632 (Selleckchem) for the first 24 h post-naive hESC plating.

### Doxycycline pulsing on DUX4-TetOn hESCs

DUX4-TetOn hESCs were incubated with 1 μg/mL of doxycycline in Essential 8 culture medium in 5% CO_2_ incubator at 37°C for varied times as indicated. After the doxycycline induction, the DUX4-TetOn hESCs were washed three times with Essential 8 culture medium and incubated thereafter in Essential 8 medium for the indicated times.

### RNA extraction and quantitative real-time PCR

Total RNA was isolated using NucleoSpin RNA kit (Macherey Nagel) according to the manufacturer’s protocol. For cDNA synthesis, 500 ng of total RNA was reverse-transcribed by MMLV-RTase (Promega) with oligo dT priming. The resulting cDNA was used as a template for quantitative real-time PCR using 5× HOT FIREPol qPCR Mix (Solis BioDyne) on the LightCycler 96 System (Roche). Relative expression values were calculated with the 2^−ΔΔCt^ method (Livak and Schmittgen, 2001), using cyclophilin G (*PPIG*) as an internal control, normalized against the untreated cells (Figure S2C) or NaPi2b^−^ cells (Figure 4F).

### STRT whole-culture RNA-seq library preparation and sequencing

Doxycycline-induced and control DUX4-TetOn hESCs were collected for STRT whole-culture RNA-seq immediately, 24 h, and 48 h after 15 min or 30 min of doxycycline or without doxycycline treatment. FACS-sorted cells were collected from three independent experiments as described later. Naive H9 hESCs were collected by hand picking the colonies from the cell culture dishes, using sterile needles. Conventional primed H9 hESCs were collected by washing the cells once with PBS and lysing the culture according to the NucleoSpin RNA kit protocol. We used 16–20 ng of RNA to generate a 48-plex RNA-seq library using a modified STRT method with unique molecular identifiers (UMIs) (Ezer et al., 2021; Islam et al., 2011, 2014). Briefly, RNA samples were placed in a 48-well plate, and a universal primer, template-switching oligo-nucleotides, and a well-specific 6-bp barcode sequence (for sample identification) were added to each well (Katayama et al., 2013; Krjutškov et al., 2016). We pooled the synthesized cDNAs into one library, performed fragmentation to 200–400 bp using an M220 Focused-ultrasonicator (Covaris), captured the 5′ fragments, added an adapter, and amplified the targets by PCR. The RNA-seq library was sequenced with Illumina NextSeq 500 System, High Output mode.

### scRNA-seq library preparation and sequencing

DUX4-TetOn hESCs were seeded into three plates at each experiment, two doxycycline-treated and one untreated, and then collected at 6, 12, 24, and 48 h after treatment. The cells were washed once with PBS and incubated with TrypLE Express for 4 min. TrypLE was diluted with Essential 8 medium, and the cell suspensions were filtered through 40 μm Cell Strainers. Cell suspensions were centrifuged at 400 rcf for 8 min. Cell pellets were resuspended each in 100 μL of Dead Cell Removal Kit microbeads (Miltenyi Biotec) and incubated at room temperature for 15 min. After incubation, each cell-microbead suspension was gently resuspended to 800 μL of freshly prepared 1× binding buffer. Cell suspensions were pipetted to magnetic MS columns (Miltenyi Biotec) 500 μL at a time and let flow through. The columns were washed three times with 1× binding buffer. The cell suspensions were centrifuged at 400 rcf for 5 min, and the pellets were resuspended each in 400 μL of 10x Genomics sample buffer. The cells were counted, and the volumes were adjusted to approximately 1,200 cells/μL of suspension. The samples were kept on ice prior to analysis of cell quality and number and preparation of the scRNA sequencing libraries. Approximately 94% of the nucleated cells were alive. The libraries were prepared using Chromium Next GEM Single Cell 3′ Gene Expression v.3.1 chemistry and sequencing was performed using Illumina NovaSeq 6000 system at the Institute for Molecular Medicine Finland (FIMM) Single-Cell Analytics unit.

### Immunocytochemical staining of DUX4-TetOn hESCs

Cells were fixed on Ibidi eight-well *μ* slides with 3.8% paraformaldehyde at room temperature for 15 min and washed three times with PBS. For the nuclear epitopes, the cells were permeabilized using 0.5% Triton X-100-PBS at room temperature for 7 min. The cells were washed once with PBS, and unspecific binding of antibodies was blocked by Ultravision Protein Block solution (Thermo Fisher Scientific) by a 10-min incubation at room temperature. Primary antibodies were diluted in washing buffer (0.1% Tween20-PBS) and incubated at 4°C overnight. Excess primary antibody solutions were removed, and the cells were washed three times with washing buffer. The secondary antibodies were diluted 1:1,000 in washing buffer and incubated at room temperature for 30 min. The samples were washed three times with washing buffer, and nuclei were counterstained with DAPI, diluted 1:1,000 in washing buffer. The samples were washed once and kept in PBS for imaging. Protocols of western blotting and annexin V staining are provided in supplemental experimental procedures. All the antibodies used in this study are listed in the key resources table.

### Confocal microscopy and image analysis

Images were captured with a Leica TCS SP8 confocal laser scanning microscope (Leica Microsystems, Mannheim, Germany) using Leica HC PL APO CS2 40×/1.10NA water objective and 1,024 × 1,024 scan format. For annexin V stainings, the cells were imaged with a Leica TCS SP8 X confocal microscope with white laser. The images were captured with either 20× air objective or 63× oil objective using 1,024 × 1,024 scan format. The data were processed using Fiji (http://fiji.sc; Schindelin et al., 2012). The images were softened using Gaussian filter (radius = 1-pixel kernel). Fluorescence intensity was quantified using Fiji by segmenting the regions of interest with the Otsu thresholding method (Otsu, 1979). The mean fluorescence intensities of POU5F1 staining were compared between LEUTX-positive (intensity ≥ 10) and negative (intensity < 10) cells.

### FACS of DUX4-TetOn hESCs

The DUX4-TetOn hESCs were washed once with PBS and incubated with TrypLE Express for 4 min in 5% CO_2_ incubator at 37°C. The TrypLE Express was diluted with cold FACS buffer (5% fetal bovine serum in PBS supplemented with 10 μM ROCK inhibitor Y-27632), and the cell suspensions were let flow through 40 μm Cell Strainers. The cells were counted, and approximately 5 × 10^5^ cells were aliquoted per Eppendorf tube. From here, on the cells were kept on ice. The cells were centrifuged at 4°C, 300 rcf for 5 min. The primary anti-NaPi2b antibody, mouse MX35, a kind gift from Dr. Gerd Ritter, was diluted 1:100 (final concentration 20 μg/mL) in FACS buffer. The cells were incubated for 1 h on ice for primary antibody staining (MX35). The samples were washed three times with FACS buffer by centrifugation as above. Secondary antibody Alexa-Fluor-594-conjugated donkey anti-mouse (A-21203, Thermo Fisher Scientific), was diluted 1:1,000 in FACS buffer and incubated with cells on ice for 30 min. The cells were washed three times as above. The cells were analyzed and separated using Sony SH800Z Cell Sorter (Sony Biotechnology), using 100 μm nozzle. Altogether 5 × 10^5^ cells were collected for follow-up culture. The cells were centrifuged at 4°C, 300 rcf for 5 min, resuspended in Essential 8 culture medium with 10 μM ROCK inhibitor, and cultured for 6 h in 5% CO_2_, at 37°C, prior to cell lysis for RNA isolation.

### STRT RNA-seq data processing

The sequenced STRT RNA-seq raw reads were processed as described elsewhere (https://github.com/my0916/STRT2; Ezer et al., 2021). Two samples collected immediately after induction were excluded due to a low number of mapped reads. The STRT RNA-seq data of continuous *DUX4* induction, treated by doxycycline for 4 h, were obtained from Vuoristo et al. (2022) and reprocessed as described elsewhere (https://github.com/my0916/STRT2; Ezer et al., 2021). The list of EGA genes was retrieved from Töhönen et al. (2015; Table S1), and TFEs overlapped with these gene regions were analyzed further. The list of DUX4 target genes expressed in the cleavage-stage human embryo was retrieved from Resnick et al. (2019; Table S1). The list of stage-specific genes was retrieved from Stirparo et al. (2018). The STRT RNA-seq data of HS980 primed ESCs and eight-cell stage cells were obtained from Jouhilahti et al. (2016). Detailed analysis methods are provided in the supplemental experimental procedures.

### scRNA-seq data processing

The raw BCL files were demultiplexed and converted to FASTQ files with Cell Ranger (10x Genomics, v.3.1.0) mkfastq and mapped against the customized human reference genome (GRCh38 with DUX4-IRES-EmGFP) with STAR (Dobin et al., 2013). The cellranger aggr pipeline was used to combine all the data to generate a gene-count matrix. The output count data were subsequently analyzed with the R package Seurat (v.4.0.0) (Hao et al., 2021). To measure the expression of *DUX4*, we quantified the expression of DUX4-IRES-EmGFP to avoid problems of mapping to the D4Z4 repeat locus. Gene expression scores of each signature were calculated using the gene signature scoring function retrieved from Liu et al. (2020). The list of EGA genes was obtained from Töhönen et al. (2015), and that of signature genes of primed and naive PSCs was obtained from Liu et al. (2020). The list of eight-cell and ESC genes was retrieved from Jouhilahti et al. (2016; Table S1). The list of 299 LEUTX-target genes was retrieved from our STRT RNA-seq data of *LEUTX*-inducible hESCs (L.G., E.-M.J., M.Y., Fei Liangru, J.W., Tomi T. Airenne, R.T., Shruti Bhagat, M.H.T., Yasuhiro Murakawa, Kari Salokas, Xiaonan Liu, Sini Miettinen, S.V., Thomas R. Bürglin, Biswajyoti Sahu, T.O., Mark S. Johnson, S.K., M.V., J.K., unpublished data; Table S1). Cell type annotation was conducted with the R package SingleR (v.1.4.1) (Aran et al., 2019), using the scRNA-seq data of human preimplantation embryos and ESCs (Yan et al., 2013) as the reference data. Pseudotime trajectory analysis was performed using the R package Monocle (v.2.18.0) (Qiu et al., 2017). scRNA-seq data of human preimplantation embryos (Petropoulos et al., 2016) and naive and primed hESCs (Messmer et al., 2019) were obtained from the ArrayExpress database with the accession number E-MTAB-3929 and E-MTAB-6819, respectively. These data were processed and integrated with our scRNA-seq dataset of *DUX4*-pulsed hESCs using the FindIntegrationAnchors and IntegrateData functions in Seurat. Our cells were randomly downsampled to 400 cells per cluster so that the number of cells was comparable between different datasets. Detailed analysis methods are provided in the supplemental experimental procedures.

### Data and code availability

The STRT whole-culture RNA-seq and scRNA-seq data of DUX4-TetOn hESCs used in this study have been deposited in the ArrayExpress database at EMBL-EBI and are available under the accession codes “E-MTAB-10569” and “E-MTAB-10581,” respectively.

## Author contributions

Conceptualization, M.Y., S.V., and J.K.; data curation, M.Y.; formal analysis, M.Y. and J.S.; funding acquisition, M.Y., T.O., S.V., and J.K.; investigation, M.Y., I.K., S.N., J.S., K.L., L.G., E.-M.J., M.V., M.H.T., and S.V.; methodology, M.Y., J.W., R.T., and S.V.; project administration, J.K.; resources, R.T., S.K., and S.V.; software, M.Y. and S.K.; supervision, T.O., R.T., S.K., S.V., and J.K.; validation, M.Y., I.K., S.N., and S.V.; visualization, M.Y. and S.V.; writing – original draft, M.Y. and S.V.; writing – review & editing, all authors.

## Conflicts of interests

The authors declare no competing interests.

## Supplemental information

Document S1. Figures S1–S4 and supplemental experimental procedures

Table S1. List of genes used in this study, related to Figures 1, 2, and 3

Table S2. Quality metrics of the scRNA-seq data in each experiment, related to Figures 2 and 3

Table S3. List of marker genes in each cluster, related to Figure 3

Table S4. List of differentially expressed genes along the pseudotime (q < 1e−100) from intermediate to iBM transition, related to Figure 3

Document S2. Article plus supplemental information

## References

[bib1] Aran D., Looney A.P., Liu L., Wu E., Fong V., Hsu A., Chak S., Naikawadi R.P., Wolters P.J., Abate A.R. (2019). Reference-based analysis of lung single-cell sequencing reveals a transitional profibrotic macrophage. Nat. Immunol..

[bib2] Bosnakovski D., Xu Z., Gang E.J., Galindo C.L., Liu M., Simsek T., Garner H.R., Agha-Mohammadi S., Tassin A., Coppée F. (2008). An isogenetic myoblast expression screen identifies DUX4-mediated FSHD-associated molecular pathologies. EMBO J..

[bib3] Braude P., Bolton V., Moore S. (1988). Human gene expression first occurs between the four- and eight-cell stages of preimplantation development. Nature.

[bib4] De Iaco A., Planet E., Coluccio A., Verp S., Duc J., Trono D. (2017). DUX-family transcription factors regulate zygotic genome activation in placental mammals. Nat. Genet..

[bib5] De Iaco A., Coudray A., Duc J., Trono D. (2019). DPPA2 and DPPA4 are necessary to establish a 2C-like state in mouse embryonic stem cells. EMBO Rep..

[bib6] Dobin A., Davis C.A., Schlesinger F., Drenkow J., Zaleski C., Jha S., Batut P., Chaisson M., Gingeras T.R. (2013). STAR: ultrafast universal RNA-seq aligner. Bioinformatics.

[bib7] Ezer S., Yoshihara M., Katayama S., Daub C., Lohi H., Krjutskov K., Kere J. (2021). Generation of RNA sequencing libraries for transcriptome analysis of globin-rich tissues of the domestic dog. STAR Protoc..

[bib8] Fu X., Wu X., Djekidel M.N., Zhang Y. (2019). Myc and Dnmt1 impede the pluripotent to totipotent state transition in embryonic stem cells. Nat. Cell Biol..

[bib9] Fu X., Djekidel M.N., Zhang Y. (2020). A transcriptional roadmap for 2C-like-to-pluripotent state transition. Sci. Adv..

[bib10] Genet M., Torres-Padilla M.E. (2020). The molecular and cellular features of 2-cell-like cells: a reference guide. Development.

[bib11] Geng L.N., Yao Z., Snider L., Fong A.P., Cech J.N., Young J.M., van der Maarel S.M., Ruzzo W.L., Gentleman R.C., Tawil R. (2012). DUX4 activates germline genes, retroelements, and immune mediators: implications for facioscapulohumeral dystrophy. Dev. Cell.

[bib12] Hao Y., Hao S., Andersen-Nissen E., Mauck W.M., Zheng S., Butler A., Lee M.J., Wilk A.J., Darby C., Zager M. (2021). Integrated analysis of multimodal single-cell data. Cell.

[bib13] Hendrickson P.G., Doráis J.A., Grow E.J., Whiddon J.L., Lim J.W., Wike C.L., Weaver B.D., Pflueger C., Emery B.R., Wilcox A.L. (2017). Conserved roles of mouse DUX and human DUX4 in activating cleavage-stage genes and MERVL/HERVL retrotransposons. Nat. Genet..

[bib14] Islam S., Zeisel A., Joost S., La Manno G., Zajac P., Kasper M., Lönnerberg P., Linnarsson S. (2014). Quantitative single-cell RNA-seq with unique molecular identifiers. Nat. Methods.

[bib15] Islam S., Kjällquist U., Moliner A., Zajac P., Fan J.B., Lönnerberg P., Linnarsson S. (2011). Characterization of the single-cell transcriptional landscape by highly multiplex RNA-seq. Genome Res..

[bib16] Jouhilahti E.M., Madissoon E., Vesterlund L., Töhönen V., Krjutškov K., Plaza Reyes A., Petropoulos S., Månsson R., Linnarsson S., Bürglin T. (2016). The human PRD-like homeobox gene LEUTX has a central role in embryo genome activation. Development.

[bib17] Jukam D., Shariati S.A.M., Skotheim J.M. (2017). Zygotic genome activation in vertebrates. Dev. Cell.

[bib18] Katayama S., Töhönen V., Linnarsson S., Kere J. (2013). SAMstrt: statistical test for differential expression in single-cell transcriptome with spike-in normalization. Bioinformatics.

[bib19] Kowaljow V., Marcowycz A., Ansseau E., Conde C.B., Sauvage S., Mattéotti C., Arias C., Corona E.D., Nuñez N.G., Leo O. (2007). The DUX4 gene at the FSHD1A locus encodes a pro-apoptotic protein. Neuromuscul. Disord..

[bib20] Krjutškov K., Katayama S., Saare M., Vera-Rodriguez M., Lubenets D., Samuel K., Laisk-Podar T., Teder H., Einarsdottir E., Salumets A. (2016). Single-cell transcriptome analysis of endometrial tissue. Hum. Reprod..

[bib21] Liu X., Nefzger C.M., Rossello F.J., Chen J., Knaupp A.S., Firas J., Ford E., Pflueger J., Paynter J.M., Chy H.S. (2017). Comprehensive characterization of distinct states of human naive pluripotency generated by reprogramming. Nat. Methods.

[bib22] Liu X., Ouyang J.F., Rossello F.J., Tan J.P., Davidson K.C., Valdes D.S., Schröder J., Sun Y.B.Y., Chen J., Knaupp A.S. (2020). Reprogramming roadmap reveals route to human induced trophoblast stem cells. Nature.

[bib23] Livak K.J., Schmittgen T.D. (2001). Analysis of relative gene expression data using real-time quantitative PCR and the 2(-Delta Delta C(T)) Method. Methods.

[bib24] Macfarlan T.S., Gifford W.D., Driscoll S., Lettieri K., Rowe H.M., Bonanomi D., Firth A., Singer O., Trono D., Pfaff S.L. (2012). Embryonic stem cell potency fluctuates with endogenous retrovirus activity. Nature.

[bib25] Mazid M.A., Ward C., Luo Z., Liu C., Li Y., Lai Y., Wu L., Li J., Jia W., Jiang Y. (2022). Rolling back human pluripotent stem cells to an eight-cell embryo-like stage. Nature.

[bib26] Messmer T., von Meyenn F., Savino A., Santos F., Mohammed H., Lun A.T.L., Marioni J.C., Reik W. (2019). Transcriptional heterogeneity in naive and primed human pluripotent stem cells at single-cell resolution. Cell Rep..

[bib27] Otsu N. (1979). A threshold selection method from gray-level histograms. IEEE Trans. Syst. Man Cybern..

[bib28] Petropoulos S., Edsgärd D., Reinius B., Deng Q., Panula S.P., Codeluppi S., Plaza Reyes A., Linnarsson S., Sandberg R., Lanner F. (2016). Single-cell RNA-seq reveals lineage and X chromosome dynamics in human preimplantation embryos. Cell.

[bib29] Qiu X., Mao Q., Tang Y., Wang L., Chawla R., Pliner H.A., Trapnell C. (2017). Reversed graph embedding resolves complex single-cell trajectories. Nat. Methods.

[bib30] Resnick R., Wong C.J., Hamm D.C., Bennett S.R., Skene P.J., Hake S.B., Henikoff S., van der Maarel S.M., Tapscott S.J. (2019). DUX4-Induced histone variants H3.X and H3.Y mark DUX4 target genes for expression. Cell Rep..

[bib31] Rickard A.M., Petek L.M., Miller D.G. (2015). Endogenous DUX4 expression in FSHD myotubes is sufficient to cause cell death and disrupts RNA splicing and cell migration pathways. Hum. Mol. Genet..

[bib32] Rodriguez-Terrones D., Gaume X., Ishiuchi T., Weiss A., Kopp A., Kruse K., Penning A., Vaquerizas J.M., Brino L., Torres-Padilla M.E. (2018). A molecular roadmap for the emergence of early-embryonic-like cells in culture. Nat. Genet..

[bib33] Schindelin J., Arganda-Carreras I., Frise E., Kaynig V., Longair M., Pietzsch T., Preibisch S., Rueden C., Saalfeld S., Schmid B. (2012). Fiji: an open-source platform for biological-image analysis. Nat. Methods.

[bib34] Schlaeger T.M., Daheron L., Brickler T.R., Entwisle S., Chan K., Cianci A., DeVine A., Ettenger A., Fitzgerald K., Godfrey M. (2015). A comparison of non-integrating reprogramming methods. Nat. Biotechnol..

[bib35] Shadle S.C., Zhong J.W., Campbell A.E., Conerly M.L., Jagannathan S., Wong C.J., Morello T.D., van der Maarel S.M., Tapscott S.J. (2017). DUX4-induced dsRNA and MYC mRNA stabilization activate apoptotic pathways in human cell models of facioscapulohumeral dystrophy. PLoS Genet..

[bib36] Stirparo G.G., Boroviak T., Guo G., Nichols J., Smith A., Bertone P. (2018). Integrated analysis of single-cell embryo data yields a unified transcriptome signature for the human pre-implantation epiblast. Development.

[bib37] Taubenschmid-Stowers J., Rostovskaya M., Santos F., Ljung S., Argelaguet R., Krueger F., Nichols J., Reik W. (2022). 8C-like cells capture the human zygotic genome activation program in vitro. Cell Stem Cell.

[bib38] Töhönen V., Katayama S., Vesterlund L., Jouhilahti E.M., Sheikhi M., Madissoon E., Filippini-Cattaneo G., Jaconi M., Johnsson A., Bürglin T.R. (2015). Novel PRD-like homeodomain transcription factors and retrotransposon elements in early human development. Nat. Commun..

[bib39] Töhönen V., Katayama S., Vesterlund L., Sheikhi M., Antonsson L., Filippini-Cattaneo G., Jaconi M., Johnsson A., Linnarsson S., Hovatta O. (2017). Transcription activation of early human development suggests DUX4 as an embryonic regulator. bioRxiv.

[bib40] Vuoristo S., Bhagat S., Hydén-Granskog C., Yoshihara M., Gawriyski L., Jouhilahti E.M., Ranga V., Tamirat M., Huhtala M., Kirjanov I. (2022). DUX4 is a multifunctional factor priming human embryonic genome activation. iScience.

[bib41] Wallace L.M., Garwick S.E., Mei W., Belayew A., Coppee F., Ladner K.J., Guttridge D., Yang J., Harper S.Q. (2011). DUX4, a candidate gene for facioscapulohumeral muscular dystrophy, causes p53-dependent myopathy in vivo. Ann. Neurol..

[bib42] Whiddon J.L., Langford A.T., Wong C.J., Zhong J.W., Tapscott S.J. (2017). Conservation and innovation in the DUX4-family gene network. Nat. Genet..

[bib43] Yan L., Yang M., Guo H., Yang L., Wu J., Li R., Liu P., Lian Y., Zheng X., Yan J. (2013). Single-cell RNA-Seq profiling of human preimplantation embryos and embryonic stem cells. Nat. Struct. Mol. Biol..

[bib44] Yang F., Huang X., Zang R., Chen J., Fidalgo M., Sanchez-Priego C., Yang J., Caichen A., Ma F., Macfarlan T. (2020). DUX-miR-344-ZMYM2-Mediated activation of MERVL LTRs induces a totipotent 2C-like state. Cell Stem Cell.

[bib45] Yang M., Yu H., Yu X., Liang S., Hu Y., Luo Y., Izsvák Z., Sun C., Wang J. (2022). Chemical-induced chromatin remodeling reprograms mouse ESCs to totipotent-like stem cells. Cell Stem Cell.

[bib46] Yin B.W., Kiyamova R., Chua R., Caballero O.L., Gout I., Gryshkova V., Bhaskaran N., Souchelnytskyi S., Hellman U., Filonenko V. (2008). Monoclonal antibody MX35 detects the membrane transporter NaPi2b (SLC34A2) in human carcinomas. Cancer Immun..

[bib47] Young J.M., Whiddon J.L., Yao Z., Kasinathan B., Snider L., Geng L.N., Balog J., Tawil R., van der Maarel S.M., Tapscott S.J. (2013). DUX4 binding to retroelements creates promoters that are active in FSHD muscle and testis. PLoS Genet..

