# Supplementary material for: Transient DUX4 expression in human embryonic stem cells induces blastomere-like expression program that is marked by SLC34A2
Source: Stem Cell Reports. 2022 Jun 30;17(7):1743–56. doi: 10.1016/j.stemcr.2022.06.002 (PMC9287684; doi:10.1016/j.stemcr.2022.06.002)
Supplement: Document S1. Figures S1–S4 and supplemental experimental procedures [file mmc1.pdf]

Stem Cell Reports, Volume *17*

Supplemental Information

Transient *DUX4* expression in human embryonic stem cells induces blastomere-like expression program that is marked by SLC34A2

Masahito Yoshihara, Ida Kirjanov, Sonja Nykänen, Joonas Sokka, Jere Weltner, Karolina Lundin, Lisa Gawriyski, Eeva-Mari Jouhilahti, Markku Varjosalo, Mari H. Tervaniemi, Timo Otonkoski, Ras Trokovic, Shintaro Katayama, Sanna Vuoristo, and Juha Kere

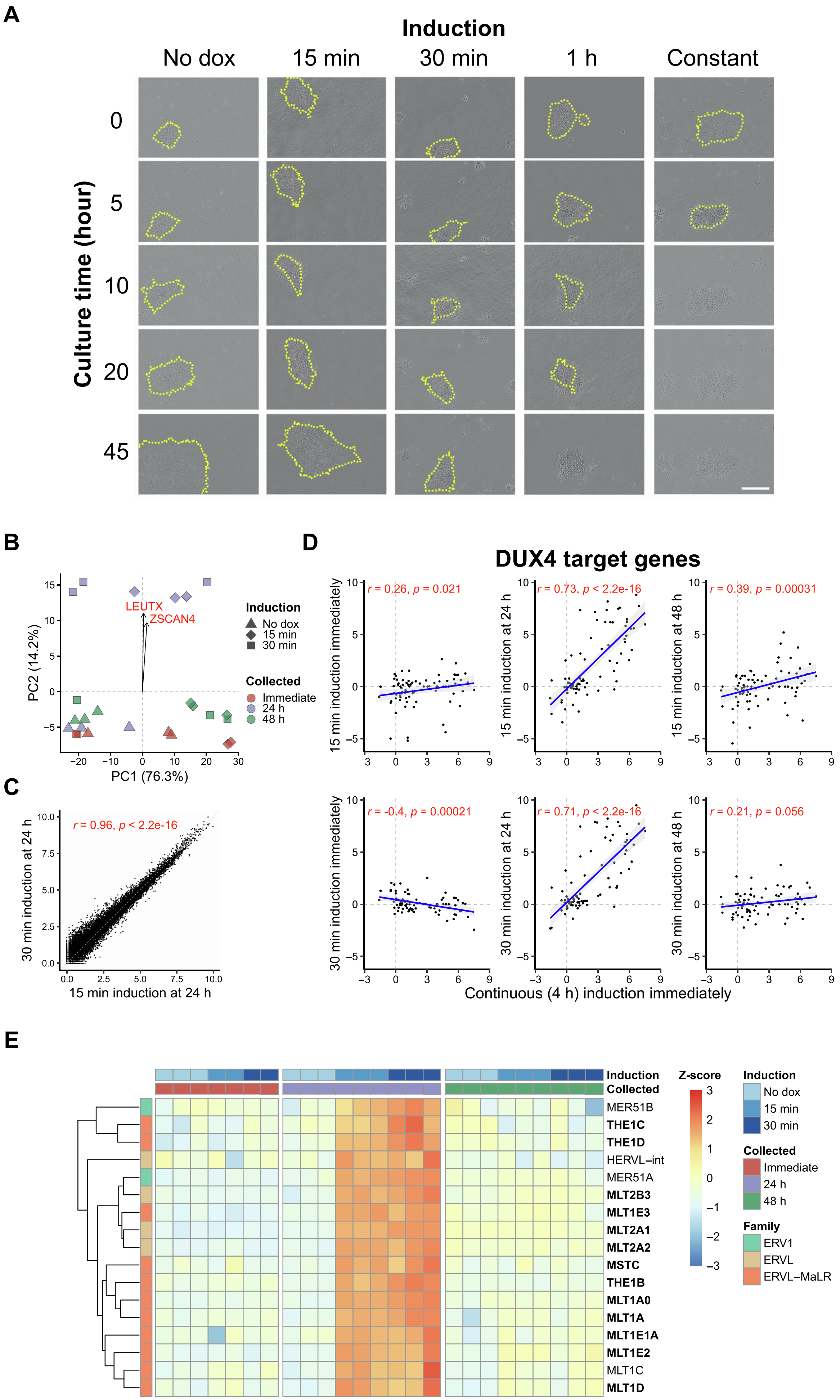

A
Induction
No dox
15 min
30 min
1 h
Constant
Culture time (hour)
0
5
10
20
45
B
PC2 (14.2%)
PC1 (76.3%)
LEUTX
ZSCAN4
Induction
No dox
15 min
30 min
Collected
Immediate
24 h
48 h
C
r = 0.96, p < 2.2e-16
30 min induction at 24 h
15 min induction at 24 h
D
DUX4 target genes
r = 0.26, p = 0.021
r = 0.73, p < 2.2e-16
r = 0.39, p = 0.00031
r = -0.4, p = 0.00021
r = 0.71, p < 2.2e-16
r = 0.21, p = 0.056
15 min induction immediately
15 min induction at 24 h
15 min induction at 48 h
30 min induction immediately
30 min induction at 24 h
30 min induction at 48 h
Continuous (4 h) induction immediately
E
Induction
Collected
MER51B
THE1C
THE1D
HERVL–int
MER51A
MLT2B3
MLT1E3
MLT2A1
MLT2A2
MSTC
THE1B
MLT1A0
MLT1A
MLT1E1A
MLT1E2
MLT1C
MLT1D
Z-score
Induction
No dox
15 min
30 min
Collected
Immediate
24 h
48 h
Family
ERV1
ERVL
ERVL–MaLR

Figure S1. Effect of transient *DUX4* induction in hESCs, related to Figure 1

(A) Bright field microscopy images of DUX4-TetOn hESCs after varied times of doxycycline induction. Living cells used for the measurement of colony size are surrounded by yellow lines. Scale bars, 200 μm.

(B) Principal component analysis of the STRT whole-culture RNA-seq data. Arrows show the variables of *LEUTX* and *ZSCAN4* on PC1 and PC2.

(C) Correlation of gene expression profiles of DUX4-TetOn hESCs at 24 h after 15 min (x-axis) and 30 min (y-axis) of induction. Expression levels are shown as log normalized counts.

(D) Transcriptional changes of 80 DUX4 target genes after continuous *DUX4* induction (x-axis) and transient *DUX4* induction (y-axis). Axes show the $\log_2$ fold expression changes over no *DUX4* induction. The correlation coefficients (r) and *P*-values were calculated using a two-sided Spearman's correlation test. The linear regression line (blue) and 95% confidence interval (gray shaded) are shown. See also Table S1.

(E) Heatmap showing the expression of repetitive elements. Repetitive elements significantly upregulated at 24 h after 15 min and 30 min of induction are shown. Elements bound by DUX4 (Young et al., 2013) are shown in bold.

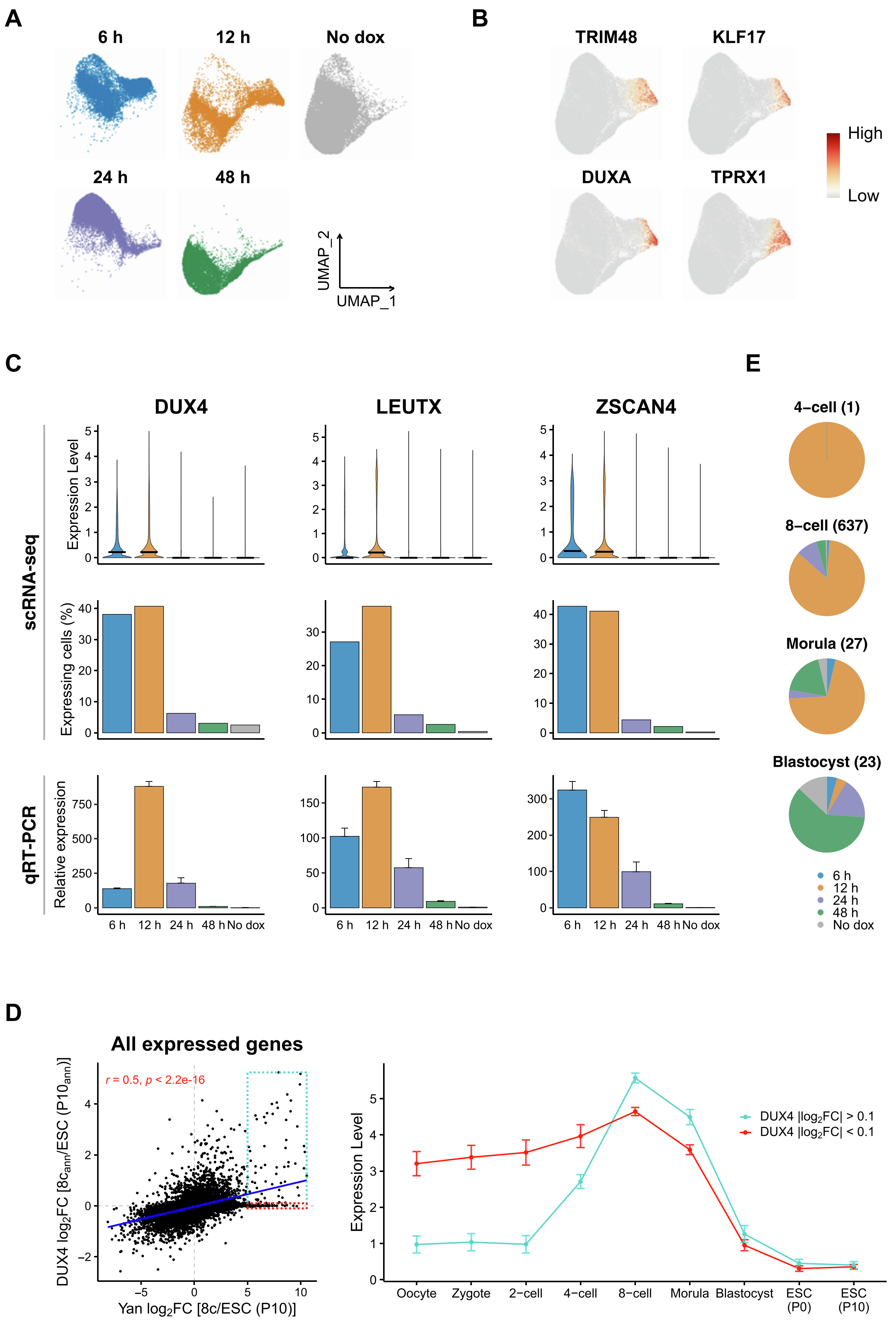

A
6 h
12 h
No dox
24 h
48 h
UMAP_2
UMAP_1
B
TRIM48
KLF17
DUXA
TPRX1
High
Low
C
DUX4
LEUTX
ZSCAN4
scRNA-seq
Expression Level
Expressing cells (%)
qRT-PCR
Relative expression
6 h 12 h 24 h 48 h No dox
E
4−cell (1)
8−cell (637)
Morula (27)
Blastocyst (23)
6 h
12 h
24 h
48 h
No dox
D
All expressed genes
r = 0.5, p < 2.2e-16
DUX4 log2FC [8cann/ESC (P10ann)]
Yan log2FC [8c/ESC (P10)]
Expression Level
DUX4 |log2FC| > 0.1
DUX4 |log2FC| < 0.1
Oocyte
Zygote
2−cell
4−cell
8−cell
Morula
Blastocyst
ESC (P0)
ESC (P10)

Figure S2. Time-course analysis of *DUX4*-pulsed hESCs at single-cell level, related to Figure 2

(A) UMAP plot colored by collected time points. Note that there are few untreated (No dox) cells in the rightmost cluster.

(B) Expression pattern of DUX4 target genes projected onto the UMAP plot.

(C) Expression of *DUX4* and its target genes by collected time points. Top: expression levels shown as log normalized UMI counts. Middle: proportion of expressing cells (UMI count > 1). Bottom: expression in whole-culture cells measured by quantitative real-time (qRT)-PCR. Error bars represent the SEM of three different culture plates.

(D) Transcriptional changes of 15,902 genes in actual eight-cell stage cells (x-axis) and *DUX4*-pulsed hESCs annotated as eight-cell stage cells (y-axis) compared with hESCs. Axes show the $\log_2$ fold expression changes over hESCs (P10; x-axis) or cells annotated as hESCs (P10; y-axis). $8c_{ann}$, cells annotated as eight-cell stage cells; ESC ($P10_{ann}$), cells annotated as ESC (P10). Right panel shows the mean expression (log FPKM) during early development of 64 differentially expressed genes ($|\log_2 FC| > 0.1$; lightblue) and 72 unchanged genes ($|\log_2 FC| < 0.1$; red) by *DUX4* induction. Error bars denote SEM.

(E) Proportion of collected time points in cells annotated as early embryonic stage cells.

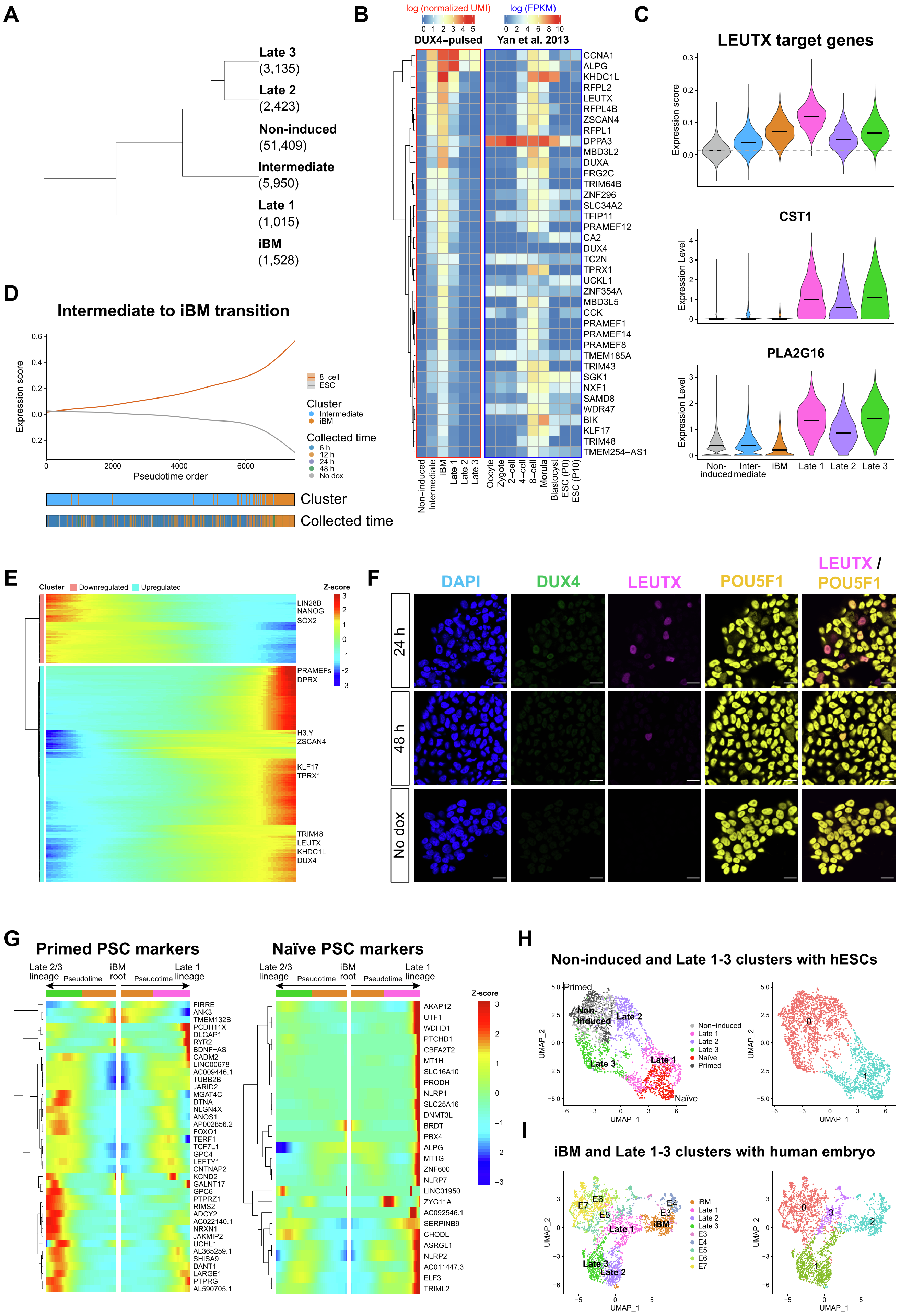
A
Late 3 (3,135)
Late 2 (2,423)
Non-induced (51,409)
Intermediate (5,950)
Late 1 (1,015)
iBM (1,528)
B
log (normalized UMI)
log (FPKM)
DUX4–pulsed
Yan et al. 2013
CCNA1
ALPG
KHDC1L
RFPL2
LEUTX
RFPL4B
ZSCAN4
RFPL1
DPPA3
MBD3L2
DUXA
FRG2C
TRIM64B
ZNF296
SLC34A2
TFIP11
PRAMEF12
CA2
DUX4
TC2N
TPRX1
UCKL1
ZNF354A
MBD3L5
CCK
PRAMEF1
PRAMEF14
PRAMEF8
TMEM185A
TRIM43
SGK1
NXF1
SAMD8
WDR47
BIK
KLF17
TRIM48
TMEM254–AS1
Non–induced
Intermediate
iBM
Late 1
Late 2
Late 3
Oocyte
Zygote
2–cell
4–cell
8–cell
Morula
Blastocyst
ESC (P0)
ESC (P10)
C
LEUTX target genes
Expression score
CST1
Expression Level
PLA2G16
Non-induced
Inter-mediate
iBM
Late 1
Late 2
Late 3
D
Intermediate to iBM transition
Expression score
Pseudotime order
8–cell
ESC
Cluster
Intermediate
iBM
Collected time
6 h
12 h
24 h
48 h
No dox
Cluster
Collected time
E
Cluster
Downregulated
Upregulated
Z-score
LIN28B
NANOG
SOX2
PRAMEFs
DPRX
H3.Y
ZSCAN4
KLF17
TPRX1
TRIM48
LEUTX
KHDC1L
DUX4
F
DAPI
DUX4
LEUTX
POU5F1
LEUTX / POU5F1
24 h
48 h
No dox
G
Primed PSC markers
Naïve PSC markers
Late 2/3 lineage
Pseudotime
iBM root
Late 1 lineage
Z-score
FIRRE
ANK3
TMEM132B
PCDH11X
DLGAP1
RYR2
BDNF–AS
CADM2
LINC00678
AC009446.1
TUBB2B
JARID2
MGAT4C
DTNA
NLGN4X
ANOS1
AP002856.2
FOXO1
TERF1
TCF7L1
GPC4
LEFTY1
CNTNAP2
KCND2
GALNT17
GPC6
PTPRZ1
RIMS2
ADCY2
AC022140.1
NRXN1
JAKMIP2
UCHL1
AL365259.1
SHISA9
DANT1
LARGE1
PTPRG
AL590705.1
AKAP12
UTF1
WDHD1
PTCHD1
CBFA2T2
MT1H
SLC16A10
PRODH
NLRP1
SLC25A16
DNMT3L
BRDT
PBX4
ALPG
MT1G
ZNF600
NLRP7
LINC01950
ZYG11A
AC092546.1
SERPINB9
CHODL
ASRGL1
NLRP2
AC011447.3
ELF3
TRIML2
H
Non-induced and Late 1-3 clusters with hESCs
Primed
Non-induced
Late 2
Late 1
Late 3
Naïve
Non-induced
Late 1
Late 2
Late 3
Naïve
Primed
UMAP_1
UMAP_2
I
iBM and Late 1-3 clusters with human embryo
E7
E6
E5
E4
E3
Late 1
iBM
Late 3
Late 2
iBM
Late 1
Late 2
Late 3
E3
E4
E5
E6
E7
UMAP_1
UMAP_2

Figure S3. Detailed characterization of *DUX4*-pulsed hESCs at single-cell level, related to Figure 3

(A) Hierarchical clustering analysis of the 6 clusters. Numbers in parentheses indicate the number of cells.

(B) iBM cluster-marker gene expression in *DUX4*-pulsed hESCs in each cluster (left) and human preimplantation embryo (right). Clustering of genes was performed based on the expression pattern across clusters in *DUX4*-pulsed hESCs. See also Table S3.

(C) LEUTX target gene expression in each cluster. LEUTX target gene expression score was calculated with the 299 genes. The horizontal gray dotted line indicates the median score in the non-induced cluster. *CST1* and *PLA2G16* are representatives. See also Table S1.

(D) Gene expression score changes of eight-cell and ESC in cells from the intermediate and the iBM clusters along the pseudotime.

(E) Heatmap of 675 significantly changed genes ($q < 1e-100$) along the pseudotime from intermediate to iBM transition, clustered by pseudotemporal expression pattern. x-axis corresponds to the pseudotime order shown in Figure S3D. See also Table S4.

(F) Immunocytochemical detection of DUX4, LEUTX, and POU5F1 in *DUX4*-pulsed hESCs at 24 h, 48 h, and without induction (No dox). DAPI (blue) was used as nuclear counterstain. Scale bars, 20 µm.

(G) Expression changes of primed (left) and naïve (right) PSC markers along the pseudotime from iBM to late transition.

(H) Integration of non-induced and late 1–3 cluster cells with naïve and primed hESCs (Messmer et al., 2019) projected onto the UMAP plot. Non-induced and late 1–3 cluster cells were downsampled to 400 cells per cluster. Colored by original cell identity (left) and cluster annotation (right).

(I) Integration of iBM and late 1–3 cluster cells with the human embryo (Petropoulos et al., 2016) projected onto the UMAP plot. iBM and late 1–3 cluster cells were downsampled to 400 cells per cluster. Colored by original cell identity (left) and cluster annotation (right).

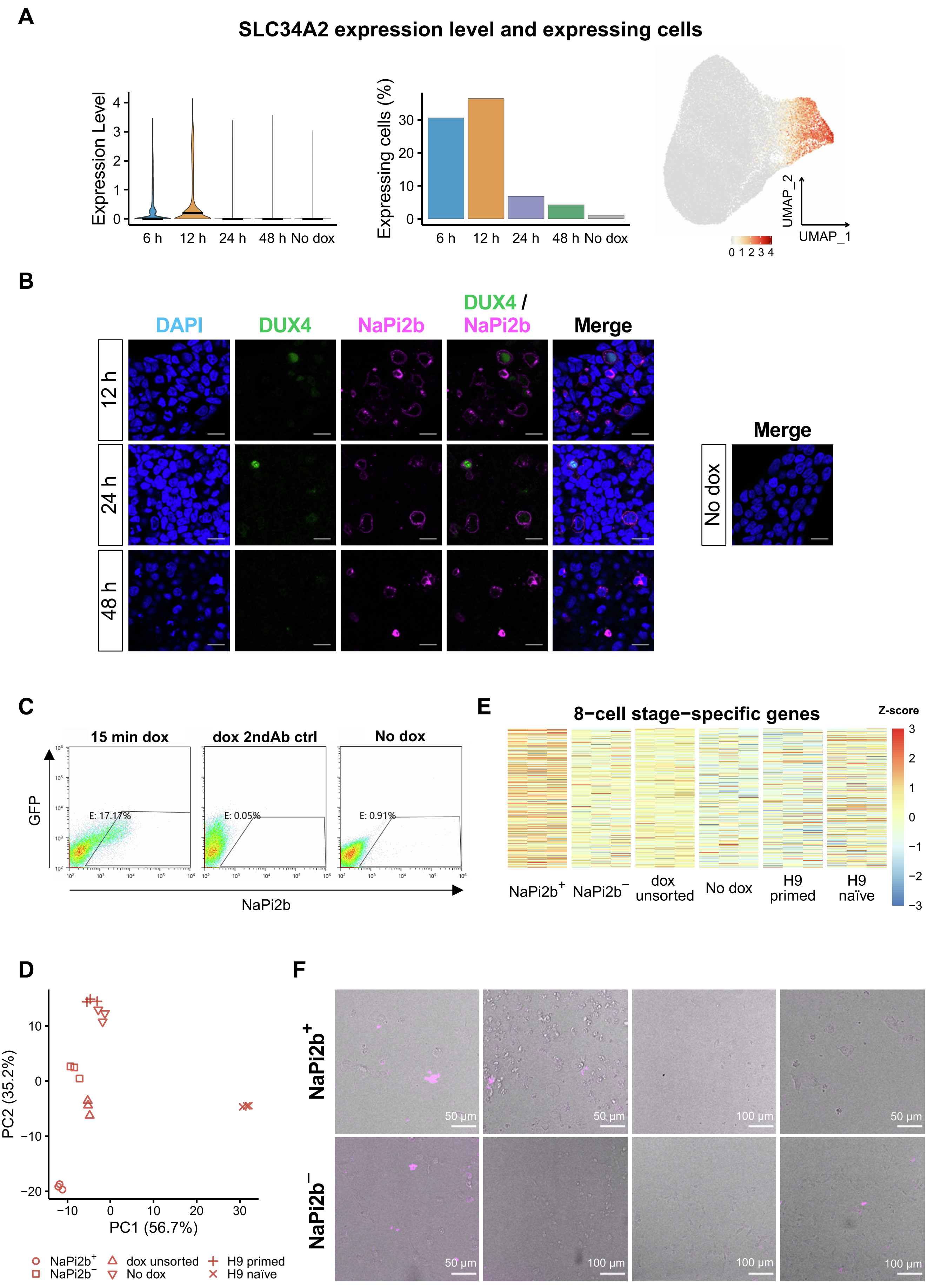
A
SLC34A2 expression level and expressing cells
Expression Level
6 h
12 h
24 h
48 h
No dox
Expressing cells (%)
UMAP_2
UMAP_1
B
DAPI
DUX4
NaPi2b
DUX4 / NaPi2b
Merge
12 h
24 h
48 h
No dox
C
15 min dox
dox 2ndAb ctrl
No dox
GFP
E: 17.17%
E: 0.05%
E: 0.91%
NaPi2b
E
8−cell stage−specific genes
Z-score
NaPi2b+
NaPi2b−
dox unsorted
No dox
H9 primed
H9 naïve
D
PC2 (35.2%)
PC1 (56.7%)
NaPi2b+
NaPi2b−
dox unsorted
No dox
H9 primed
H9 naïve
F
NaPi2b+
NaPi2b−
50 μm
100 μm

Figure S4. iBM cells could be enriched with an anti-NaPi2b antibody, related to Figure 4

(A) Left: *SLC34A2* expression levels shown as log normalized UMI counts. Middle: proportion of *SLC34A2* expressing cells (UMI count > 1). Right: *SLC34A2* expression projected onto the UMAP plot.

(B) Immunocytochemical detection of DUX4 and NaPi2b in *DUX4*-pulsed hESCs at 12 h, 24 h, and 48 h. Untreated cells (No dox) are shown in right. DAPI (blue) was used as nuclear counterstain. Scale bars, 20 μm.

(C) Flow cytometric analysis showing the gating of NaPi2b-positive cells. Representative data from two independent experiments are shown. 2ndAb ctrl, secondary antibody control.

(D) Principal component analysis of the sorted NaPi2b^{+} and NaPi2b^{-} cells, unsorted *DUX4*-pulsed hESCs (dox unsorted), no induction (No dox), and H9 primed and naïve ESCs (n = 3 independent experiments).

(E) Heatmap showing the expression of eight-cell stage-specific genes (990 genes).

(F) Annexin V staining of NaPi2b^{+} and NaPi2b^{-} cells after 6 h of culture. Scale bars are as shown in the images.

KEY RESOURCES TABLE

REAGENT or RESOURCE	SOURCE	IDENTIFIER
**Antibodies**		
Mouse monoclonal anti-DUX4	Merck Millipore	Cat# MABD116; clone 9A12
Rabbit monoclonal anti-DUX4	Abcam	Cat# ab124699; RRID: AB_10973363; clone E5-5
Goat polyclonal anti-OCT3/4 (N-19)	Santa Cruz Biotechnology	Cat# sc-8628; RRID: AB_653551
Rabbit polyclonal anti-KLF17	Atlas Antibodies	Cat# HPA024629; RRID: AB_1668927
Rabbit polyclonal anti-LEUTX	Novus Biologicals	Cat# NBP1-90890; RRID: AB_11053314
Rabbit polyclonal anti-Cleaved Caspase-3 (Asp175)	Cell Signaling Technology	Cat# 9661; RRID: AB_2341188
Mouse monoclonal anti-NaPi2b (MX35)	Memorial Sloan Kettering	
Donkey Anti-Mouse IgG (H+L) (Alexa Fluor® 647)	Thermo Fisher Scientific	Cat# A31571; RRID: AB_162542
Donkey Anti-Rabbit IgG (H+L) (Alexa Fluor® 488)	Thermo Fisher Scientific	Cat# A21206; RRID: AB_2535792
Donkey Anti-Goat IgG (H+L) (Alexa Fluor® 594)	Thermo Fisher Scientific	Cat# A11058; RRID: AB_2534105
Donkey Anti-Mouse IgG (H+L) (Alexa Fluor® 488)	Thermo Fisher Scientific	Cat# A21202; RRID: AB_141607
Donkey Anti-Rabbit IgG (H+L) (Alexa Fluor® 594)	Thermo Fisher Scientific	Cat# A21207; RRID: AB_141637
Donkey Anti-Rabbit IgG HRP Conjugate	Jackson ImmunoResearch	Cat# 711-035-152; RRID: AB_10015282
Annexin V-Cy5 Apoptosis Detection Kit	Abcam	Cat# ab14150
**Chemicals, peptides, and recombinant proteins**		
Geltrex	Thermo Fisher Scientific	Cat# A1413302
Essential 8 culture medium	Thermo Fisher Scientific	Cat# A1517001
NaïveCult Expansion Medium	Stemcell Technologies	Cat# 05590
EDTA	Thermo Fisher Scientific	Cat# 15575-020
MMLV-RTase	Promega	Cat# M1701
5× HOT FIREPol qPCR Mix	Solis BioDyne	Cat# 08-25-00020
TrypLE Express Enzyme	Thermo Fisher Scientific	Cat# 12604-021
Ultravision protein Block solution	Thermo Fisher Scientific	Cat# TA-060-PBQ
ROCK inhibitor: Y-27632	Selleckchem	Cat# S1049
DPBS, no calcium, no magnesium	Thermo Fisher Scientific	Cat# 14200
Tween20	Fisher Scientific	Cat# BP337-100
Triton X-100	Fisher Scientific	Cat# BP151-100

4× Laemmli Sample Buffer	Bio-Rad	Cat# 161-0747
Clarity Western ECL Substrate	Bio-Rad	Cat# 170-5061
Critical commercial assays		
NucleoSpin RNA kit	Macherey Nagel	Cat# 740955
Dead Cell Removal Binding Kit	Miltenyi Biotec	Cat# 130-090-101
MS Columns	Miltenyi Biotec	Cat# 130-042-201
Chromium Next GEM Single Cell 3' Kit v3.1	10x Genomics	
Deposited data		
STRT RNA-seq data of *DUX4*-pulsed hESCs	This paper	E-MTAB-10569
STRT RNA-seq data of continuously *DUX4*-induced hESCs	(Vuoristo et al., 2022)	GEO: GSE171803
STRT RNA-seq data of early human embryos	(Töhönen et al., 2015)	ENA PRJEB8994
scRNA-seq data of *DUX4*-pulsed hESCs	This paper	E-MTAB-10581
scRNA-seq data of early human embryos and hESCs	(Yan et al., 2013)	GEO: GSE36552
STRT RNA-seq data of human eight-cell stage cells and hESCs	(Jouhilahti et al., 2016)	ENA PRJEB12467
STRT RNA-seq data of *LEUTX*-inducible hESCs	Gawriyski et al., unpublished	E-MTAB-10539
scRNA-seq data of human preimplantation embryos	(Petropoulos et al., 2016)	E-MTAB-3929
scRNA-seq data of naïve and primed hESCs	(Messmer et al., 2019)	E-MTAB-6819
Experimental models: Cell lines		
Human: DUX4-TetOn hESCs	(Vuoristo et al., 2022)	N/A
H9	WiCell	WA09
Oligonucleotides		
Primer: *LEUTX* Forward: GCTACAATGGGGAAACTGGC	(Jouhilahti et al., 2016)	N/A
Primer: *LEUTX* Reverse: CTCTTCCATTTGGCACGCTG	(Jouhilahti et al., 2016)	N/A
Primer: *ZSCAN4* Forward: CCTCCCAGACTTCCCAAGAT	(Vuoristo et al., 2022)	N/A
Primer: *ZSCAN4* Reverse: TGTTCCAGCCATCTTGTTCA	(Vuoristo et al., 2022)	N/A
Primer: *TRIM48* Forward: CATCACTGGACTGAGGGACA	(Vuoristo et al., 2022)	N/A
Primer: *TRIM48* Reverse: TGACTGTTGGCTTCATTGTGA	(Vuoristo et al., 2022)	N/A
Primer: cyclophilin G (*PPIG*) Forward: TCTTGTCAATGGCCAACAGA	(Weltner et al., 2018)	N/A
Primer: cyclophilin G (*PPIG*) Reverse: GCCCATCTAAATGAGGAGTT	(Weltner et al., 2018)	N/A
Software and algorithms		
STRT2 pipeline b3e589c	(Ezer et al., 2021)	https://github.com/my0916/STRT2
Picard v2.20.4	https://github.com/broadinstitute/picard	http://broadinstitute.github.io/picard/
HISAT2 v2.1.0	(Kim et al., 2019)	https://daehwankimlab.github.io/hisat2/
featureCounts v1.5.2	(Liao et al., 2014)	http://subread.sourceforge.net/
StringTie v1.3.3	(Pertea et al., 2015)	https://ccb.jhu.edu/software/stringtie/
TEtranscripts v2.2.1	(Jin et al., 2015)	https://github.com/mhammell-laboratory/TEtranscripts

R v4.0.0	(R Development Core Team, 2020)	https://www.r-project.org/
DESeq2 v1.30.0	(Love et al., 2014)	http://www.bioconductor.org/packages/release/bioc/html/DESeq2.html
Cell Ranger v3.1.0	10x Genomics	https://support.10xgenomics.com/single-cell-gene-expression/software/pipelines/latest/what-is-cell-ranger
STAR aligner	(Dobin et al., 2013)	https://github.com/alexdobin/STAR
Seurat v4.0.0	(Hao et al., 2021)	https://satijalab.org/seurat/
SingleR v1.4.1	(Aran et al., 2019)	https://bioconductor.org/packages/release/bioc/html/SingleR.html
Monocle v2.18.0	(Qiu et al., 2017)	http://cole-trapnell-lab.github.io/monocle-release/
STRTprep	(Krjutškov et al., 2016)	https://github.com/shka/STRTprep
ImageLab	Bio-Rad	http://www.bio-rad.com/en-ch/product/image-lab-software?ID=KRE6P5E8Z
Fiji	(Schindelin et al., 2012)	https://fiji.sc/

Supplemental Tables in Excel files

Table S1. List of genes used in this study, related to Figures 1–3.

Table S2. Quality metrics of the scRNA-seq data in each experiment, related to Figures 2 and 3.

Table S3. List of marker genes in each cluster, related to Figure 3.

Table S4. List of differentially expressed genes along the pseudotime (q < 1e-100) from intermediate to iBM transition, related to Figure 3.

Supplemental experimental procedures

Western blotting

Cells were washed with PBS and lysed with RIPA buffer (Thermo Scientific). Lysate was centrifuged at 14,000 rcf for 15 min and supernatant was collected to a new tube. Protein samples were prepared using 4× Laemmli Sample Buffer (Bio-Rad) with 10% beta mercaptoethanol and boiled at 100°C for 5 min. Samples were loaded on Mini-PROTEAN TGX Stain-Free Gels (Bio-Rad) and run with 1× Tris-Glycine-SDS (Bio-Rad) buffer in Mini-PROTEAN Tetra Vertical Electrophoresis Cell (Bio-Rad). Proteins were transferred using the Trans Blot Turbo device (Bio-Rad) and Trans-Blot Turbo Transfer Pack (Bio-Rad). Membranes were quickly soaked first in dH_2O and then in 70% EtOH and blocked in 5% skim milk diluted in 1× Tris Buffered Saline with 0.05% Tween 20 (TBST) for 1 h. After blocking, membrane was washed three times with TBST and incubated with DUX4 antibody (diluted 1:1000 in 3% Skim Milk-TBST) overnight at 4°C. The next day, the membrane was washed three times with TBST on shaker for 10 min and incubated with HRP-conjugated anti-rabbit IgG (diluted 1:40000 in 3% Skim Milk-TBST) at room temperature for 1 h. The membrane was washed three times with TBST on shaker for 10 min and developed in Clarity Western ECL Substrate (Bio-Rad) at room temperature for 5 min. Membrane was imaged with ChmeiDoc MP Imaging System. Image was analyzed with ImageLab Software (Bio-Rad) by normalizing DUX4 to total protein.

Annexin V staining

The cells were washed with Annexin V binding buffer and incubated in Annexin V solution (1:100 in binding buffer) at room temperature in dark, for 5 min. The cells were washed with Annexin V Binding buffer, fixed with 2% paraformaldehyde at room temperature in dark, for 10 min. The cells were washed twice with PBS and imaged.

STRT RNA-seq data processing

The sequenced STRT RNA-seq raw reads were processed as described elsewhere (https://github.com/my0916/STRT2) (Ezer et al., 2021). Briefly, raw base call (BCL) files were demultiplexed and converted to FASTQ files with Picard tools (v2.20.4; http://broadinstitute.github.io/picard/), and aligned to the human reference genome hg19, human ribosomal DNA unit (GenBank: U13369), and ERCC spike-ins (SRM 2374) with the GENCODE (v28) transcript annotation by HISAT2 (v2.1.0) (Kim et al., 2019). Potential PCR duplicates were flagged with Picard MarkDuplicates. For gene-based analysis, uniquely mapped reads within the 5'-UTR or 500 bp upstream of the protein-coding genes were counted using Subread featureCounts (v1.5.2) (Liao et al., 2014) with '−−ignoreDup' option. The mapped reads were further assembled by StringTie (v1.3.3) (Pertea et al., 2015) and those reads within the first exons of the assembled transcripts (TFEs) were counted as previously described (Töhönen et al., 2015). Two samples collected immediately after induction were

excluded due to a low number of mapped reads. PCA was performed using the top 500 most variable genes. The STRT RNA-seq data of continuous *DUX4* induction, treated by doxycycline for 4 h, was obtained from Vuoristo et al. (Vuoristo et al., 2022) and reprocessed as described above. The expression of transposable elements was quantified using TEtranscripts (v2.2.1) with 'uniq' mode (Jin et al., 2015). Differential expression analysis between doxycycline-induced and non-induced cells was performed with the R (v4.0.0) package DESeq2 (v1.30.0) (Love et al., 2014), and the expression values were normalized by the library size calculated with DESeq2. Genes or transposable elements with Benjamini–Hochberg-adjusted *P*-value < 0.05 were considered statistically significant. The correlation coefficients (r) and *P*-values were calculated using a two-sided Spearman's correlation test. The list of EGA genes was retrieved from Töhönen et al. (Töhönen et al., 2015) (**Table S1**), and TFEs overlapped with these gene regions were analyzed further. The list of DUX4 target genes expressed in the cleavage-stage human embryo was retrieved from Resnick et al. (Resnick et al., 2019) (**Table S1**). The list of stage-specific genes was retrieved from Stirparo et al. (Stirparo et al., 2018). The STRT RNA-seq data of HS980 primed ESCs and eight-cell stage cells was obtained from Jouhilahti et al. (Jouhilahti et al., 2016). For the integration of the two STRT RNA-seq datasets, log-normalized expression values were quantile normalized and subtracted by the mean of each gene across the samples in each dataset (Liu et al., 2017). The multidimensional scaling analysis was performed using the cmdscale function based on the Spearman correlation distance matrix between the samples of the two datasets.

scRNA-seq data processing

Data pre-processing and cell clustering

The raw BCL files were demultiplexed and converted to FASTQ files with Cell Ranger (10x Genomics, v3.1.0) mkfastq, and mapped against the customized human reference genome (GRCh38 with DUX4-IRES-EmGFP) with STAR (Dobin et al., 2013). The cellranger aggr pipeline was used to combine all the data to generate a gene-count matrix. The output count data were subsequently analyzed with the R package Seurat (v4.0.0) (Hao et al., 2021). Cells with 15,000–100,000 UMI counts, expressing over 3,500 genes and less than 15% mitochondrial counts were kept, resulting in 65,460 cells in total. These data were then processed using the NormalizeData and FindVariableFeatures (using 2,000 features) functions. Next, cell-cycle scores were calculated using the CellCycleScoring function, and data scaling was performed with the ScaleData function, regressing out the S and G2M scores. Principal component analysis (PCA) was performed on the scaled data using the RunPCA function, and cell clustering was performed using the FindNeighbours (using the top 10 PCs) and FindClusters (resolution = 0.6) functions. UMAP was implemented on the top 10 PCs with the RunUMAP function. Here, 10 clusters with lower UMAP_1 values (left clusters) showing similar expression profiles were mainly composed of no-dox cells and were assigned as the 'non-induced' cluster. The dendrogram was generated with the BuildClusterTree function. To measure the expression of *DUX4*, we quantified the expression of DUX4-IRES-EmGFP to avoid problems of mapping to the D4Z4 repeat locus. Average expression level in each

cluster was calculated with the AverageExpression function. The iBM cluster specific markers shown in Figure S3B were identified by the FindAllMarkers function and selected as pct.1 > 0.8, pct.2 < 0.5, and avg_logFC > 1.

Gene expression scoring and cell type annotation

Gene expression scores of each signature were calculated using the gene signature scoring function retrieved from Liu et al. (Liu et al., 2020). Briefly, the average expression values of the genes of interest were subtracted by the aggregated expression values of a set of randomly selected control genes at similar expression level. The list of EGA genes were obtained from Töhönen et al. (Töhönen et al., 2015), and that of signature genes of primed and naïve PSCs were obtained from Liu et al. (Liu et al., 2020). The list of eight-cell and ESC genes were retrieved from Jouhilahti et al. (Jouhilahti et al., 2016), where the top 121 and 119 differentially expressed genes based on the differential expression score by STRTprep (Krjutškov et al., 2016) were selected, respectively (**Table S1**). The list of 299 LEUTX-target genes were retrieved from the significantly upregulated genes in our unpublished STRT RNA-seq data on *LEUTX*-inducible hESCs (Gawriyski et al., unpublished) (**Table S1**). Cell type annotation was conducted with the R package SingleR (v1.4.1) (Aran et al., 2019), using the scRNA-seq data of human preimplantation embryos and ESCs (Yan et al., 2013) as the reference data.

Pseudotime trajectory analysis

Pseudotime trajectory analysis was performed using the R package Monocle (v2.18.0) (Qiu et al., 2017) for two groups of clusters: i) intermediate and iBM clusters, ii) iBM and late 1–3 clusters, respectively. Cluster marker genes (**Table S3**) identified by the FindAllMarkers function in Seurat were used for ordering the cells, and dimensionality was reduced using the DDRTree algorithm. A generalized additive model (GAM) was fitted to the scaled expression values calculated by Seurat and the pseudotime order of cells using the geom_smooth function of the R package ggplot2 (v3.3.3). Heatmaps were generated with the Monocle plot_pseudotime_heatmap and plot_genes_branched_heatmap functions.

Integration of scRNA-seq datasets

scRNA-seq data of human preimplantation embryos (Petropoulos et al., 2016) and naïve and primed hESCs (Messmer et al., 2019) were obtained from the ArrayExpress database with the accession number E-MTAB-3929 and E-MTAB-6819, respectively. These data were processed and integrated with our scRNA-seq dataset of *DUX4*-pulsed hESCs using the FindIntegrationAnchors and IntegrateData functions in Seurat with dimensionality of 30. Our cells were randomly downsampled to 400 cells per cluster so that the number of cells was comparable between different datasets (1,529 human embryonic cells and 836 hESCs). Identical cells from each cluster were used for the integration with different datasets.

Supplemental References

Aran, D., Looney, A.P., Liu, L., Wu, E., Fong, V., Hsu, A., Chak, S., Naikawadi, R.P., Wolters, P.J., Abate, A.R., *et al.* (2019). Reference-based analysis of lung single-cell sequencing reveals a transitional profibrotic macrophage. Nat Immunol *20*, 163-172.

Dobin, A., Davis, C.A., Schlesinger, F., Drenkow, J., Zaleski, C., Jha, S., Batut, P., Chaisson, M., and Gingeras, T.R. (2013). STAR: ultrafast universal RNA-seq aligner. Bioinformatics *29*, 15-21.

Ezer, S., Yoshihara, M., Katayama, S., Daub, C., Lohi, H., Krjutskov, K., and Kere, J. (2021). Generation of RNA sequencing libraries for transcriptome analysis of globin-rich tissues of the domestic dog. STAR Protoc *2*, 100995.

Hao, Y., Hao, S., Andersen-Nissen, E., Mauck, W.M., 3rd, Zheng, S., Butler, A., Lee, M.J., Wilk, A.J., Darby, C., Zager, M., *et al.* (2021). Integrated analysis of multimodal single-cell data. Cell *184*, 3573-3587.e3529.

Jin, Y., Tam, O.H., Paniagua, E., and Hammell, M. (2015). TEtranscripts: a package for including transposable elements in differential expression analysis of RNA-seq datasets. Bioinformatics *31*, 3593-3599.

Jouhilahti, E.M., Madissoon, E., Vesterlund, L., Töhönen, V., Krjutškov, K., Plaza Reyes, A., Petropoulos, S., Månsson, R., Linnarsson, S., Bürglin, T., *et al.* (2016). The human PRD-like homeobox gene LEUTX has a central role in embryo genome activation. Development *143*, 3459-3469.

Kim, D., Paggi, J.M., Park, C., Bennett, C., and Salzberg, S.L. (2019). Graph-based genome alignment and genotyping with HISAT2 and HISAT-genotype. Nat Biotechnol *37*, 907-915.

Krjutškov, K., Katayama, S., Saare, M., Vera-Rodriguez, M., Lubenets, D., Samuel, K., Laisk-Podar, T., Teder, H., Einarsdottir, E., Salumets, A., *et al.* (2016). Single-cell transcriptome analysis of endometrial tissue. Hum Reprod *31*, 844-853.

Liao, Y., Smyth, G.K., and Shi, W. (2014). featureCounts: an efficient general purpose program for assigning sequence reads to genomic features. Bioinformatics *30*, 923-930.

Liu, X., Nefzger, C.M., Rossello, F.J., Chen, J., Knaupp, A.S., Firas, J., Ford, E., Pflueger, J., Paynter, J.M., Chy, H.S., *et al.* (2017). Comprehensive characterization of distinct states of human naive pluripotency generated by reprogramming. Nat Methods *14*, 1055-1062.

Liu, X., Ouyang, J.F., Rossello, F.J., Tan, J.P., Davidson, K.C., Valdes, D.S., Schröder, J., Sun, Y.B.Y., Chen, J., Knaupp, A.S., *et al.* (2020). Reprogramming roadmap reveals route to human induced trophoblast stem cells. Nature *586*, 101-107.

Love, M.I., Huber, W., and Anders, S. (2014). Moderated estimation of fold change and dispersion for RNA-seq data with DESeq2. Genome Biol *15*, 550.

Messmer, T., von Meyenn, F., Savino, A., Santos, F., Mohammed, H., Lun, A.T.L., Marioni, J.C., and Reik, W. (2019). Transcriptional Heterogeneity in Naive and Primed Human Pluripotent Stem Cells at Single-Cell Resolution. Cell Rep *26*, 815-824.e814.

Pertea, M., Pertea, G.M., Antonescu, C.M., Chang, T.C., Mendell, J.T., and Salzberg, S.L. (2015). StringTie enables improved reconstruction of a transcriptome from RNA-seq reads. Nat Biotechnol *33*, 290-295.

Petropoulos, S., Edsgärd, D., Reinius, B., Deng, Q., Panula, S.P., Codeluppi, S., Plaza Reyes, A., Linnarsson, S., Sandberg, R., and Lanner, F. (2016). Single-Cell RNA-Seq Reveals Lineage and X Chromosome Dynamics in Human Preimplantation Embryos. Cell *165*, 1012-1026.

Qiu, X., Mao, Q., Tang, Y., Wang, L., Chawla, R., Pliner, H.A., and Trapnell, C. (2017). Reversed graph embedding resolves complex single-cell trajectories. Nat Methods *14*, 979-982.

R Development Core Team (2020). R: A language and environment for statistical computing. (R Foundation for Statistical Computing, Vienna, Austria).

Resnick, R., Wong, C.J., Hamm, D.C., Bennett, S.R., Skene, P.J., Hake, S.B., Henikoff, S., van der Maarel, S.M., and Tapscott, S.J. (2019). DUX4-Induced Histone Variants H3.X and H3.Y Mark DUX4 Target Genes for Expression. Cell Rep *29*, 1812-1820.e1815.

Stirparo, G.G., Boroviak, T., Guo, G., Nichols, J., Smith, A., and Bertone, P. (2018). Integrated analysis of single-cell embryo data yields a unified transcriptome signature for the human pre-implantation epiblast. Development *145,* dev158501.

Töhönen, V., Katayama, S., Vesterlund, L., Jouhilahti, E.M., Sheikhi, M., Madissoon, E., Filippini-Cattaneo, G., Jaconi, M., Johnsson, A., Bürglin, T.R*., et al.* (2015). Novel PRD-like homeodomain transcription factors and retrotransposon elements in early human development. Nat Commun *6*, 8207.

Vuoristo, S., Bhagat, S., Hydén-Granskog, C., Yoshihara, M., Gawriyski, L., Jouhilahti, E.M., Ranga, V., Tamirat, M., Huhtala, M., Kirjanov, I*., et al.* (2022). DUX4 is a multifunctional factor priming human embryonic genome activation. iScience *25*, 104137.

Weltner, J., Balboa, D., Katayama, S., Bespalov, M., Krjutskov, K., Jouhilahti, E.M., Trokovic, R., Kere, J., and Otonkoski, T. (2018). Human pluripotent reprogramming with CRISPR activators. Nat Commun *9*, 2643.

Yan, L., Yang, M., Guo, H., Yang, L., Wu, J., Li, R., Liu, P., Lian, Y., Zheng, X., Yan, J*., et al.* (2013). Single-cell RNA-Seq profiling of human preimplantation embryos and embryonic stem cells. Nat Struct Mol Biol *20*, 1131-1139.
