# Supplementary material for: Transient DUX4 expression in human embryonic stem cells induces blastomere-like expression program that is marked by SLC34A2
Source: Stem Cell Reports. 2022 Jun 30;17(7):1743–56. doi: 10.1016/j.stemcr.2022.06.002 (PMC9287684; doi:10.1016/j.stemcr.2022.06.002)
Supplement: Document S2. Article plus supplemental information [file mmc6.pdf]

Stem Cell Reports
Article
OPEN ACCESS

Transient *DUX4* expression in human embryonic stem cells induces blastomere-like expression program that is marked by SLC34A2

Masahito Yoshihara,[1,2,3,11,*] Ida Kirjanov,[4,11] Sonja Nykänen,[4] Joonas Sokka,[5] Jere Weltner,[6,7] Karolina Lundin,[4] Lisa Gawriyski,[8] Eeva-Mari Jouhilahti,[5,9] Markku Varjosalo,[8] Mari H. Tervaniemi,[5,9] Timo Otonkoski,[5,10] Ras Trokovic,[5] Shintaro Katayama,[1,5,9] Sanna Vuoristo,[4,12,*] and Juha Kere[1,5,9,12,*]
[1]Department of Biosciences and Nutrition, Karolinska Institutet, Stockholm, Sweden
[2]Institute for Advanced Academic Research, Chiba University, Chiba, Japan
[3]Department of Artificial Intelligence Medicine, Graduate School of Medicine, Chiba University, Chiba, Japan
[4]Department of Obstetrics and Gynecology, University of Helsinki, Helsinki, Finland
[5]Research Programs Unit, Stem Cells and Metabolism and Biomedicum Stem Cell Centre, Faculty of Medicine, University of Helsinki, Helsinki, Finland
[6]Department of Clinical Science, Intervention and Technology, Karolinska Institutet, Stockholm, Sweden
[7]Division of Obstetrics and Gynecology, Karolinska University Hospital, Stockholm, Sweden
[8]Institute of Biotechnology, University of Helsinki, Helsinki, Finland
[9]Folkhälsan Research Center, Helsinki, Finland
[10]Children's Hospital, Helsinki University Central Hospital, University of Helsinki, Helsinki, Finland
[11]These authors contributed equally
[12]These authors contributed equally
*Correspondence: masahito.yoshihara@chiba-u.jp (M.Y.), sanna.vuoristo@helsinki.fi (S.V.), juha.kere@ki.se (J.K.)
https://doi.org/10.1016/j.stemcr.2022.06.002

SUMMARY

Embryonic genome activation (EGA) is critical for embryonic development. However, our understanding of the regulatory mechanisms of human EGA is still incomplete. Human embryonic stem cells (hESCs) are an established model for studying developmental processes, but they resemble epiblast and are sub-optimal for modeling EGA. DUX4 regulates human EGA by inducing cleavage-stage-specific genes, while it also induces cell death. We report here that a short-pulsed expression of *DUX4* in primed hESCs activates an EGA-like gene expression program in up to 17% of the cells, retaining cell viability. These *DUX4*-induced cells resembled eight-cell stage blastomeres and were named induced blastomere-like (iBM) cells. The iBM cells showed marked reduction of POU5F1 protein, as previously observed in mouse two-cell-like cells. Finally, the iBM cells were successfully enriched using an antibody against NaPi2b (SLC34A2), which is expressed in human blastomeres. The iBM cells provide an improved model system to study human EGA transcriptome.

INTRODUCTION

Embryonic genome activation (EGA) is a crucial process for the normal development of preimplantation embryos, where zygotic genes start to be transcribed. The timing of EGA varies among species, at the two-cell stage in mouse and at the four- to eight-cell stage in human (Braude et al., 1988; Jukam et al., 2017; Töhönen et al., 2015). Recent technological advances have enabled us to study transcriptional dynamics of EGA during the embryogenesis (Petropoulos et al., 2016; Töhönen et al., 2015; Yan et al., 2013). However, the detailed regulatory mechanisms of EGA have yet to be elucidated, especially in human, due to the limited availability of samples and ethical concerns. Therefore, there is a great need for an *in vitro* model system to investigate human EGA transcriptional program.

DUX4, a double homeobox transcription factor, is transiently expressed in the human cleavage stage embryo (Töhönen et al., 2017) and regulates human EGA by inducing transcription of cleavage-stage-specific genes and repetitive elements (De Iaco et al., 2017; Hendrickson et al., 2017; Vuoristo et al., 2022; Whiddon et al., 2017). However, *DUX4* overexpression in somatic cells leads to cell death both *in vitro* and *in vivo* (Bosnakovski et al., 2008; Kowaljow et al., 2007; Rickard et al., 2015; Wallace et al., 2011). A recent study reported that transient *DUX4* expression in human myoblasts activates its target genes with little cytotoxicity by inducing histone variants H3.X/Y, which contribute to the perdurance of DUX4 target gene expression with the open chromatin conformation (Resnick et al., 2019).

A rare cell population of mouse embryonic stem cells (mESCs) exhibit two-cell-like signatures (Macfarlan et al., 2012), with the reduced expression of pluripotency markers, such as POU5F1, SOX2, and NANOG, and increased expression of targets of DUX, the mouse ortholog of human DUX4 (Rodriguez-Terrones et al., 2018). These two-cell-like cells (2CLCs) have been used as an *in vitro* model to study mouse EGA (De Iaco et al., 2019; Genet and Torres-Padilla, 2020), and they spontaneously transit toward the pluripotent state under the culture conditions optimal for mESCs (Macfarlan et al., 2012). Recent studies have revealed that mouse 2CLCs can be induced by *Dux* expression (De Iaco et al., 2017; Fu et al., 2019; Hendrickson et al., 2017; Yang et al., 2020). These findings prompted

This is an open access article under the CC BY license (http://creativecommons.org/licenses/by/4.0/).

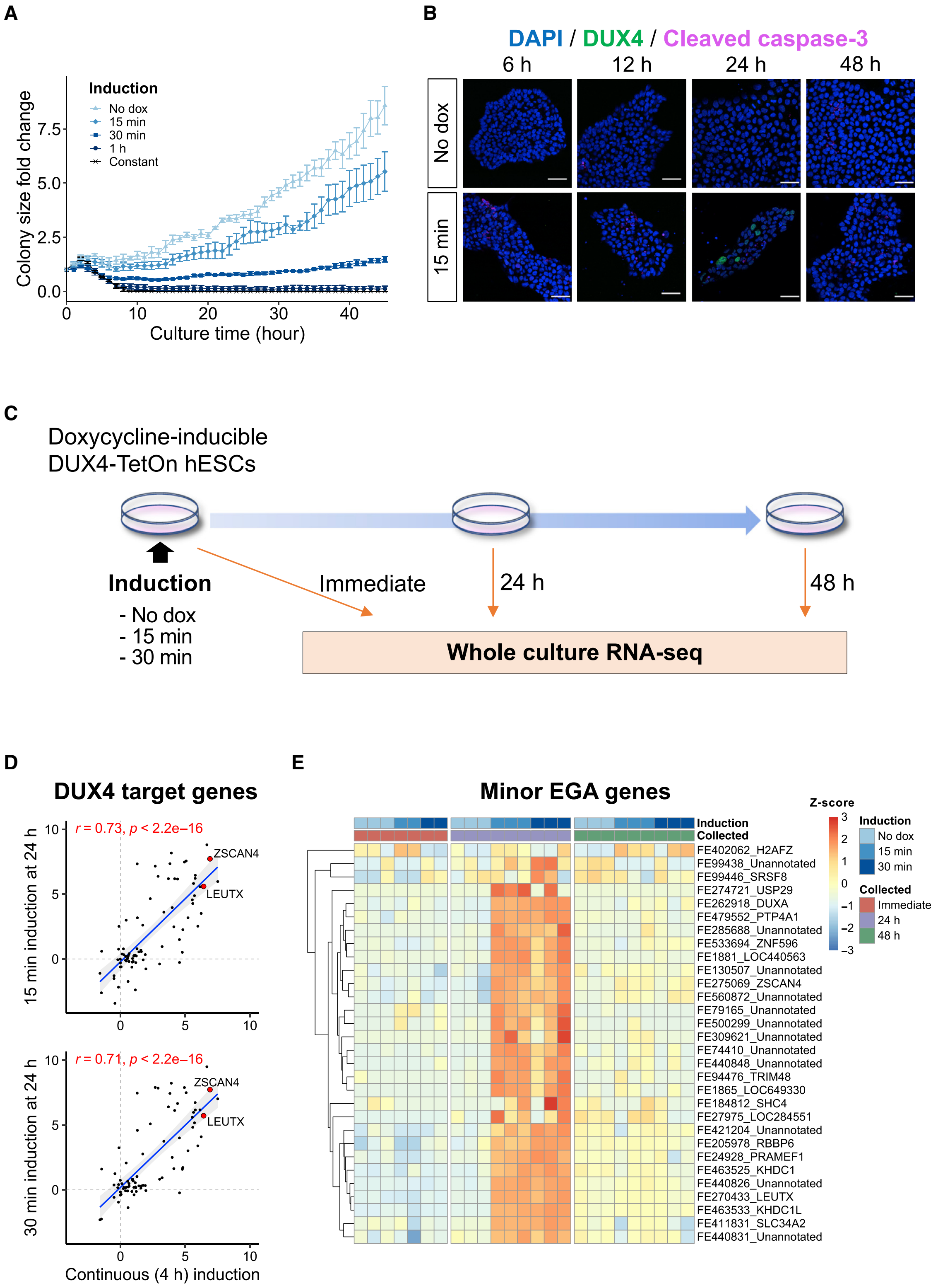

Figure 1. Transient *DUX4* induction activates EGA genes with little cellular toxicity

(A) Growth rate of DUX4-TetOn hESCs after varied times of doxycycline induction. Colony size fold change was calculated based on the starting time point (0 h). Data represent mean ± SEM (n = 5 replicates from different culture wells).

(legend continued on next page)

us to examine whether human ESCs (hESCs) could be converted to an early embryonic-like state by transient *DUX4* expression.

Here, we show that short induction of *DUX4* in primed hESCs activates EGA genes with little toxicity. We further identified a cell population, named induced blastomere-like (iBM) cells, that showed similar expression profile with eight-cell stage blastomeres. These iBM cells were enriched with fluorescence-activated cell sorting (FACS) using an antibody against a cell surface antigen, NaPi2b (SLC34A2), expressed in preimplantation embryos. The iBM cells provide a new *in vitro* model to study the mechanisms of human EGA.

RESULTS

Transient *DUX4* induction activates EGA genes in hESCs with little cytotoxicity

To test whether hESCs continue to proliferate after the transient *DUX4* induction, we first measured the expansion of the doxycycline-inducible DUX4-TetOn hESCs after various durations of doxycycline exposure (15 min, 30 min, 1 h, and constant). While doxycycline treatment for 1 h or longer caused vast cell death after prolonged culture, 15-min treatment resulted in a temporary decrease in growth rate, returning to a similar level with that of the cells without induction (Figures 1A and S1A). Moreover, only a small number of apoptotic cells were detected after 15 min of treatment, at levels similar to cells without an induction. Importantly, DUX4-positive cells were not positive for cleaved caspase-3, implying that transient *DUX4* expression did not induce apoptosis in hESCs (Figure 1B).

Next, to determine whether a transient *DUX4* induction is sufficient to activate its target genes, DUX4-TetOn hESCs were exposed to doxycycline for 15 or 30 min and subjected to RNA sequencing (RNA-seq) (Figure 1C). Principal-component analysis (PCA) demonstrated that cells collected at 24 h after either 15- or 30-min treatment were clearly separated from other samples along PC2, which was highly contributed by *LEUTX* and *ZSCAN4*, suggesting that short induction had the largest effect at 24 h post-induction (Figure S1B). Notably, cells at 24 h after 15 and 30 min of treatment showed highly similar expression profiles (r = 0.96; Spearman correlation) (Figure S1C). The expression changes of DUX4 target genes (Table S1; Resnick et al., 2019) were similar between continuously (4 h) treated cells (Vuoristo et al., 2022) and pulsed cells at 24 h post-treatment, suggesting that the 15- and 30-min pulses were sufficient to activate DUX4 target genes (Figures 1D and S1D). Furthermore, cleavage-stage-specific repetitive elements, such as MLT2A1, MLT2A2, and HERVL, activated by DUX4 (Geng et al., 2012; Hendrickson et al., 2017; Young et al., 2013), were significantly upregulated at 24 h after pulse (Figure S1E).

We previously investigated the dynamics of the human preimplantation transcriptome by single-cell tagged reverse transcriptase (STRT) RNA-seq quantifying the transcript far 5′ ends (TFEs) and identified 32 TFEs upregulated at the four-cell stage as minor EGA genes (Töhönen et al., 2015), most of which should be regulated by DUX4 (De Iaco et al., 2017). We found that 30 of them were expressed in the *DUX4*-induced hESCs, and most of them showed the highest expression at 24 h after short induction but again reduced at 48 h (Figure 1E). These observations suggest that only a 15-min induction of *DUX4* might be able to convert hESC transcriptome into a blastomere-like state.

Transient *DUX4* induction reprograms hESCs into an eight-cell-like transcriptional state

To examine whether early embryonic-like cells arise after transient *DUX4* induction, we performed time-series single-cell RNA-seq (scRNA-seq) on the DUX4-TetOn hESCs treated with doxycycline for 15 min (Figure 2A). We added two earlier time points before 24 h because the expression of DUX4 target genes might peak earlier, given their temporal expression in the embryo (De Iaco et al., 2017; Hendrickson et al., 2017). Here, we confirmed that DUX4 protein was highly expressed already at 6 h after induction but rapidly reduced and disappeared at 48 h (Figure 2B), which mimics its dynamics in the embryo (Vuoristo et al., 2022). After filtering out low-quality cells, 65,460 cells were retained for downstream analyses (Table S2). Dimensionality reduction by uniform manifold approximation and projection (UMAP) demonstrated that untreated (no dox) cells

(B) Immunocytochemical detection of DUX4 and cleaved caspase-3 in DUX4-TetOn hESCs without induction (top) and after 15 min of induction (bottom). DAPI (blue) was used as nuclear counterstain. Scale bars, 20 μm.
(C) Schematic representation of the whole culture RNA-seq on DUX4-TetOn hESCs with varied induction times.
(D) Transcriptional changes of 80 DUX4 target genes expressed in early human embryo after continuous *DUX4* induction (x axis) and transient *DUX4* induction (y axis). Axes show the $\log_2$ fold expression changes over no *DUX4* induction. The correlation coefficients (r) and p values were calculated using a two-sided Spearman's correlation test. The linear regression line (blue) and 95% confidence interval (gray shaded) are shown. See also Table S1.
(E) Heatmap showing the expression of minor EGA genes at TFE level. Of the 32 minor EGA genes, 30 genes that were expressed are shown. TFE, transcript far 5′ ends.
See also Figure S1.

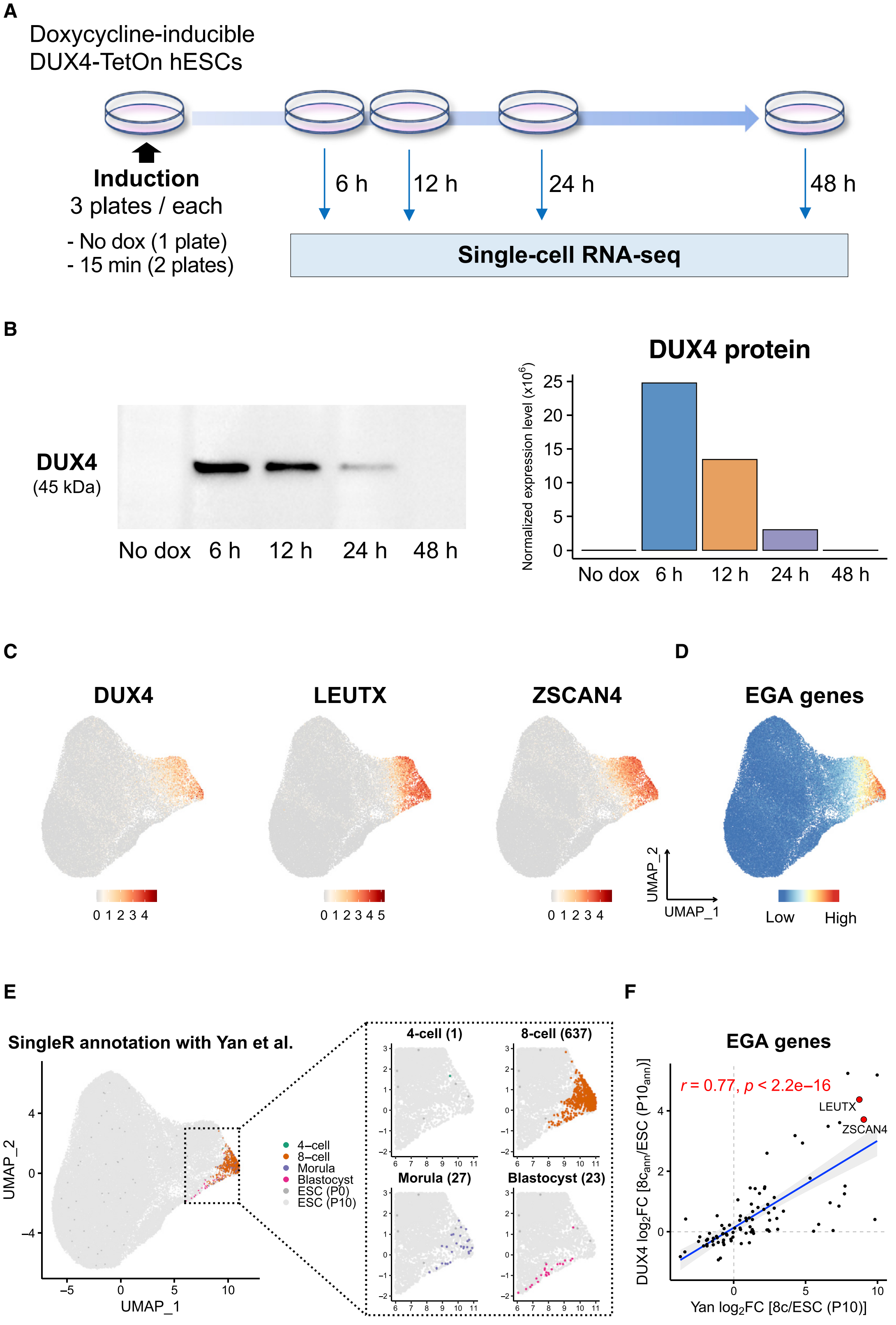

(legend on next page)

formed one main cluster, which clustered with many of the *DUX4*-pulsed cells (Figure S2A), in line with the low reprogramming efficiencies of human induced pluripotent stem cells (Schlaeger et al., 2015). *DUX4* and its target genes were highly expressed in the rightmost *DUX4*-pulsed cells along the first UMAP dimension (Figures 2C and S2B). Approximately 30%–40% of cells expressed *DUX4* and its target genes at 6 and 12 h, whereas only ~5% of cells expressed them at 24 h, in line with the quantitative real-time PCR of whole culture cells (Figure S2C). EGA genes were specifically expressed in the rightmost cluster (Figure 2D; Table S1).

To address the similarity of these cells with early human embryonic cells, we annotated the cells against the scRNA-seq data of preimplantation embryos and hESCs (Yan et al., 2013). Altogether, 637 cells were annotated as eight-cell stage cells (Figure 2E). Of these, 544 cells were collected at 12 h after induction. This indicates that 6.6% of the *DUX4*-pulsed cells (8,268 cells) were converted to a state that transcriptionally resembled eight-cell stage embryo 12 h after induction. Transcriptional changes of the EGA genes in these eight-cell-like cells correlated highly with those in eight-cell stage blastomeres (r = 0.77; Spearman correlation) (Figure 2F). Transcriptional changes of all the expressed genes were less correlated (r = 0.5; Figure S2D, left), most likely reflecting the remaining maternal transcripts that are present in the eight-cell stage blastomeres, but not activated by *DUX4* induction in hESCs. In support of this, among the genes highly expressed in eight-cell stage blastomeres ($\log_2$ FC > 5 over ESCs), the genes activated by *DUX4* induction were those most highly expressed at eight-cell stage, while the genes not activated by *DUX4* induction were highly expressed in oocytes and zygotes (Figure S2D, right). Of note, the cells annotated as four-cell, eight-cell, or morula were predominant at 12 h, whereas the cells annotated as blastocyst were predominant at 24 and 48 h (Figure S2E). These findings suggest that the transient *DUX4*-pulsed cells might recapitulate the transcriptional dynamics of EGA genes of early human embryo.

Cell-state transition dynamics after transient *DUX4* induction

To further characterize the *DUX4*-pulsed cells, we assigned them to six clusters by unsupervised clustering. Based on the proportion of the collected time points and cell type annotations, we named the clusters as follows: non-induced, intermediate, iBM, and late 1, 2, and 3 (Figure 3A). The intermediate cell cluster consisted primarily of 6-h sample cells, the iBM cluster of 12-h sample cells, and the late clusters of 24- and 48-h sample cells. Hierarchical clustering of these six clusters demonstrated that the iBM cluster showed a unique expression profile, whereas the late 2/3 clusters shared similar profile with the non-induced cluster (Figure S3A). The majority of the iBM-cluster-specific genes (Table S3) were most highly expressed at eight-cell stage and downregulated in blastocyst (Figure S3B). The intermediate and late 1 clusters moderately expressed these genes with different patterns. Although none of these genes were expressed in the non-induced cluster, *CCNA1* and *ALPG* were expressed in the late 2/3 clusters. The LEUTX target genes (Table S1; L.G., E.-M.J., M.Y., Fei Liangru, J.W., Tomi T. Airenne, R.T., Shruti Bhagat, M.H.T., Yasuhiro Murakawa, Kari Salokas, Xiaonan Liu, Sini Miettinen, S.V., Thomas R. Bürglin, Biswajyoti Sahu, T.O., Mark S. Johnson, S.K., M.V., J.K., unpublished data) were expressed higher in the late 2/3 clusters than in the non-induced cluster (Figure S3C). As *LEUTX* expression peaked in the iBM cluster (Figure S3B), the late 2/3 clusters were likely derived from the iBM cells, distinguishing them from the non-induced cluster.

To estimate the reprogramming state changes from hESCs to iBM cells, we calculated the eight-cell and ESC gene expression scores in each cell, based on our scRNA-seq

Figure 2. Time series single-cell transcriptomic profiling of *DUX4*-pulsed hESCs
(A) Schematic representation of the scRNA-seq experiments.
(B) Western blot analysis (left) and quantification (right) of DUX4 protein expression levels after 15 min of *DUX4* induction by collected time points. Expression levels were normalized to total protein.
(C) Expression of *DUX4*, *LEUTX*, and *ZSCAN4* projected onto the UMAP plot.
(D) Expression score of EGA genes projected onto the UMAP plot. See also Table S1.
(E) Cell type annotation with human preimplantation embryos and hESCs using SingleR. The right four panels show the magnified plots of the cells annotated as early embryonic stage cells. Numbers in parentheses indicate the number of the annotated cells. P0, passage 0; P10, passage 10.
(F) Transcriptional changes of 92 expressed EGA genes in actual eight-cell stage cells (x axis) and *DUX4*-pulsed hESCs annotated as eight-cell stage cells (y axis) compared with hESCs. Axes show the $\log_2$ fold expression changes over hESCs (P10; x axis) or cells annotated as hESCs (P10; y axis). The correlation coefficients (r) and p values were calculated using a two-sided Spearman's correlation test. The linear regression line (blue) and 95% confidence interval (gray shaded) are shown. $8c_{ann}$, cells annotated as eight-cell stage cells; ESC ($P10_{ann}$), cells annotated as ESC (P10).
See also Table S1 and Figure S2.

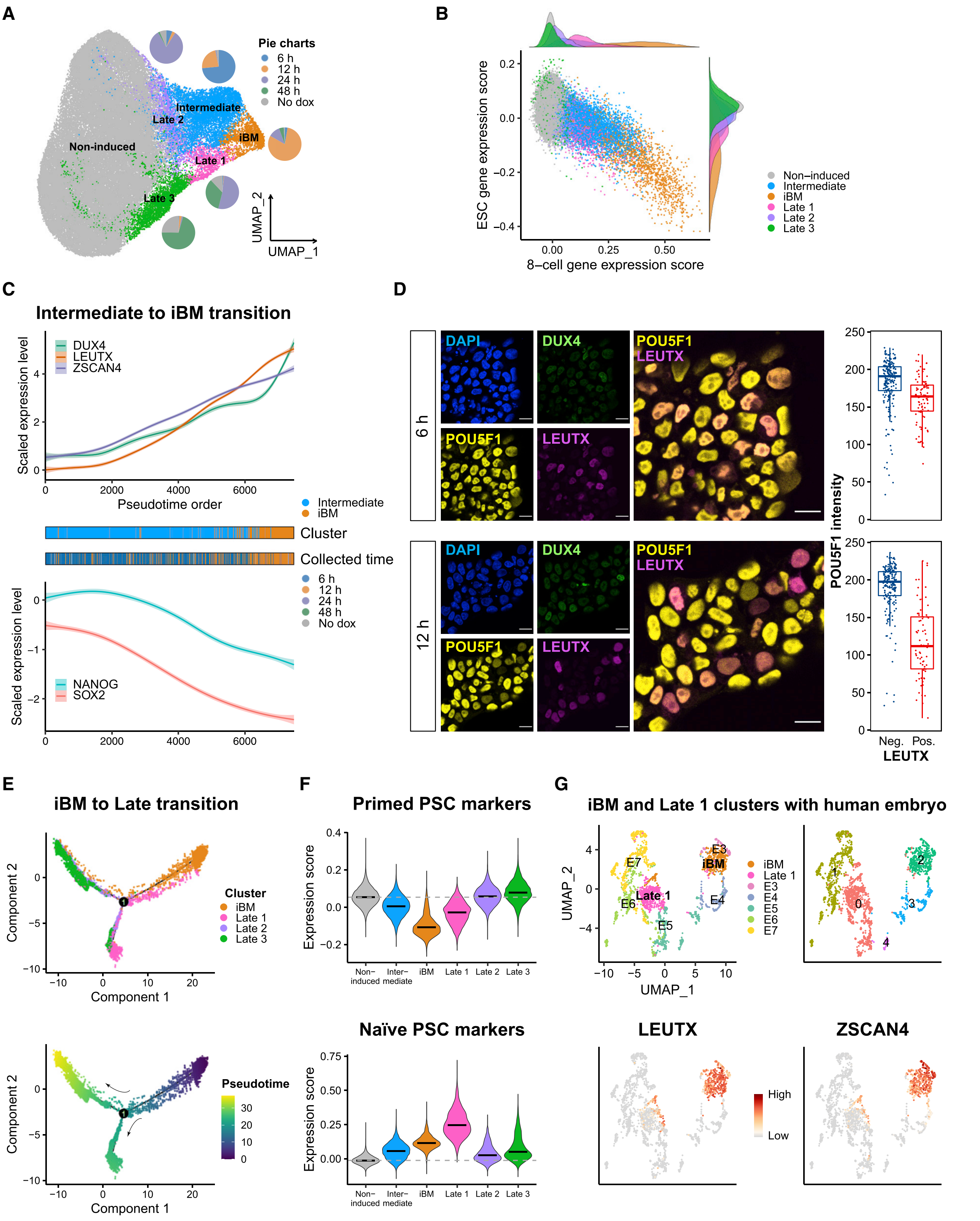

(legend on next page)

data of eight-cell stage cells and hESCs (Table S1; Jouhilahti et al., 2016). As expected, the cells in the non-induced cluster showed a high ESC score with a low eight-cell score, whereas the cells in the iBM cluster showed the lowest ESC score with the highest eight-cell score (Figure 3B). Cells in the intermediate cluster located between these two, suggesting that these cells were in the midst of the transcriptional reprogramming process. Cells in the late clusters showed higher ESC scores with lower eight-cell scores, suggesting that the iBM cells proceeded toward the ESC state.

To dissect the reprogramming process to the iBM cells via intermediate cells, we performed a pseudotime trajectory analysis on the 7,478 cells from these two clusters. The pseudotime order was consistent with the actual collected time points, with the cells collected at 6 h being earlier and 12 h later (Figure 3C). The eight-cell and ESC gene expression scores showed an inverse changing pattern along the pseudotime (Figure S3D). Expression of *DUX4* and its targets increased along the pseudotime, whereas that of pluripotency marker genes, such as *SOX2* and *NANOG*, decreased (Figures 3C and S3E; Table S4). These pluripotency marker genes are lowly expressed in cleavage-stage human embryos (Töhönen et al., 2015). DUX4 and LEUTX proteins were not detected in the untreated cells but were positive at 6 and 12 h (Figures 3D and S3F). Although *POU5F1* transcript did not significantly decrease, LEUTX-positive cells showed remarkably reduced POU5F1 staining, especially at 12 h (Figure 3D), as observed in mouse 2CLCs (Hendrickson et al., 2017; Macfarlan et al., 2012; Rodriguez-Terrones et al., 2018).

Next, to monitor the expression changes after the iBM stage, a pseudotime analysis was conducted on the 8,101 cells from the iBM and three late clusters. Cells from the iBM cluster bifurcated into two diverse branches, late 1 and late 2/3 (Figure 3E). Most of the naive pluripotent stem cell (PSC) markers (Liu et al., 2020) were upregulated along the pseudotime progression in the late 1 lineage, whereas primed PSC markers were highly expressed in the late 2/3 lineage (Figures 3F and S3G). We further directly compared the transcriptome of these cells with that of naive and primed hESCs (Messmer et al., 2019) and found that late 1 cluster cells clustered together with naive hESCs, whereas late 2/3 cluster cells clustered with primed hESCs (Figure S3H). Since naive PSCs have been described to have a similar expression profile as preimplantation epiblast (Liu et al., 2017), late 1 cluster cells were suggested to have some similarity to preimplantation embryos. To investigate the similarity of these cells to human embryos, we integrated our scRNA-seq data with that of human preimplantation embryos (Petropoulos et al., 2016). Of note, the iBM cells clustered together with eight-cell stage embryos (E3) showing similar expression levels of *LEUTX* and *ZSCAN4* (Figure 3G). Late 1 cluster cells clustered with early blastocysts (E5) (Figures 3G and S3I), whereas late 2/3 cluster cells clustered independently from the preimplantation embryos (Figure S3I). These results suggest that a majority of the iBM cells reverted to their original primed hESC state, but a subpopulation of the cells might mimic the transcriptional transition from morula to blastocyst in embryo.

Viable iBM cells can be enriched with an anti-NaPi2b antibody

Given that a subset of the *DUX4*-pulsed hESCs were classified as iBM cells, a practical method for the iBM cell enrichment is needed. We searched for a potential cell surface antigen specifically expressed in the iBM cluster (Table S3) and identified *SLC34A2*, encoding the sodium-dependent phosphate transporter NaPi2b (Figure 4A). *SLC34A2* is also one of the DUX4 target genes (Hendrickson et al.,

Figure 3. Clustering and trajectory analysis of *DUX4*-pulsed hESCs

(A) Clustering analysis of *DUX4*-pulsed hESCs with assigned cluster names. Pie charts represent the proportion of collected time points across the clusters.

(B) Expression scores of eight-cell (x axis) and ESC (y axis) of each cell, colored by clusters. Distribution of cells of each cluster is shown at the top and right. See also Table S1.

(C) Expression changes of *DUX4* and its target genes (top) and pluripotency marker genes (bottom) of single cells from the intermediate and the iBM clusters along the pseudotime. Middle panels show the cluster (above) and collected time point (below) of each single cell.

(D) Immunocytochemical detection of DUX4, LEUTX, and POU5F1 in *DUX4*-pulsed hESCs at 6 h (top) and 12 h (bottom). DAPI (blue) was used as nuclear counterstain. Scale bars, 20 μm. Mean fluorescence intensity of POU5F1 per nuclei in LEUTX-negative (Neg.) and positive (Pos.) cells is shown at the right (n = 293 for 6 h and 259 for 12 h; n denotes the number of analyzed nuclei).

(E) Trajectory reconstruction of single cells from the iBM and late clusters: pre-branch (before bifurcation), late 1 fate, and late 2/3 fate (after bifurcation). Top panel is colored by cluster, and bottom is colored by pseudotime.

(F) Expression scores of primed and naive PSC marker genes in each cluster. The horizontal black bars in violin plots indicate the median score per cluster, and the horizontal gray dotted line indicates the median score in the non-induced cluster.

(G) Integration of iBM and late 1 cluster cells with the human embryo (Petropoulos et al., 2016) projected onto the UMAP plot. iBM and late 1 cluster cells were downsampled to 400 cells per cluster. Colored by original cell identity (top left) and cluster annotation (top right) is shown. Expression levels of *LEUTX* and *ZSCAN4* in each single cell are shown at the bottom.

See also Figure S3.

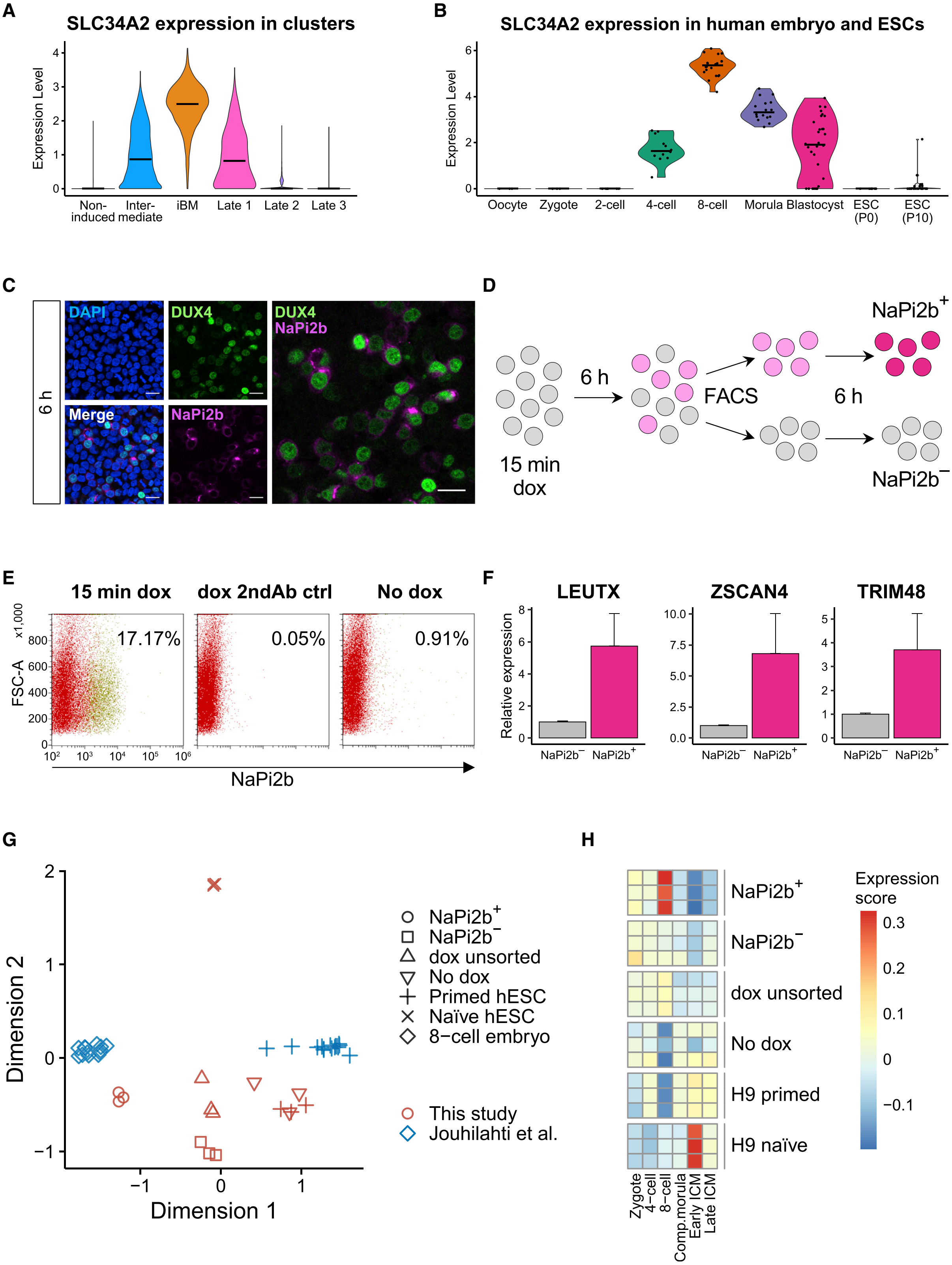

(legend on next page)

2017) and an EGA gene that is highly upregulated at the four- and eight-cell stage embryos (Figure 1E; Töhönen et al., 2015) but rarely expressed in hESCs (Figure 4B). Mouse 2CLCs also highly express *Slc34a2* (Hendrickson et al., 2017). Similar to other DUX4 target genes, *SLC34A2* was highly expressed at 6 and 12 h after induction (Figure S4A). We confirmed its expression on *DUX4*-pulsed hESCs at protein level using an anti-NaPi2b monoclonal antibody MX35, which recognizes its extracellular domain (Yin et al., 2008). MX35 specifically stained the cell surface of a subset of the *DUX4*-pulsed hESCs, already at 6 h after induction (Figures 4C and S4B).

Finally, we enriched the NaPi2b$^+$ cells by FACS at 6 h post-doxycycline treatment, and given that the iBM cells were most enriched at 12 h post-treatment, we plated the sorted cells for an additional 6 h culture (Figure 4D). The proportion of the NaPi2b$^+$ cells in the *DUX4*-pulsed hESCs was up to 17% (in two independent experiments; Figures 4E and S4C). The sorted NaPi2b$^+$ cells expressed higher levels of DUX4 target genes than the NaPi2b$^-$ cells (Figure 4F). We further characterized these cells by RNA-seq, which distinguished NaPi2b$^+$ cells from unsorted *DUX4*-pulsed hESCs or NaPi2b$^-$ cells (Figure S4D). The NaPi2b$^+$ cells showed more similar expression profile to that of eight-cell stage blastomeres (Figure 4G). Expression levels of eight-cell stage-specific genes (Stirparo et al., 2018) in the NaPi2b$^+$ cells were much higher than in the unsorted *DUX4*-pulsed hESCs and NaPi2b$^-$ cells (Figures 4H and S4E). Moreover, annexin V staining 6 h post-sorting showed that apoptotic rate between NaPi2b$^+$ and NaPi2b$^-$ cells was comparable, although slightly higher proportion of the NaPi2b$^-$ cells seemed to have attached (Figure S4F). These observations indicate that NaPi2b can be used as a marker to enrich the iBM cells by FACS.

DISCUSSION

We describe here the transcriptional reprogramming of primed hESCs into iBM cells by transient *DUX4* induction. Our data suggest that hESCs tolerate a short-term DUX4 activation with continued proliferation and without increased apoptosis. Although several studies have investigated DUX4-mediated cytotoxicity (Bosnakovski et al., 2008; Geng et al., 2012; Rickard et al., 2015; Shadle et al., 2017; Wallace et al., 2011), its mechanism is not fully understood yet. Given that DUX4 regulates human EGA genes, it remains unclear how its toxic effect is avoided in embryos. The balance between cytotoxicity and cell survival after *DUX4* induction deserves further studies.

The iBM cells share several features with mouse 2CLCs, which have been used as a model to study totipotency (Genet and Torres-Padilla, 2020). Both showed significant downregulation of *NANOG* and *SOX2* transcripts. POU5F1 protein was reduced in both cell types, although its expression was not significantly affected at transcriptional level (Fu et al., 2020; Hendrickson et al., 2017; Macfarlan et al., 2012; Rodriguez-Terrones et al., 2018). Since the late 1 cluster cells that were likely derived from iBM cells resembled early blastocyst cells, the iBM cells may provide another model with broad differentiation potential.

Finally, the iBM cells were marked by the expression of *SLC34A2*, encoding NaPi2b. Importantly, NaPi2b$^+$ cells showed higher similarity with the eight-cell stage blastomeres than other PSC types. Moreover, as an endogenous extracellular epitope, NaPi2b staining allows enrichment of the iBM cells without the need for a transgenic reporter construct. We envision that NaPi2b may be of use for isolating and culturing human eight-cell-like cells that were recently discovered among naive hESCs (Taubenschmid-Stowers et al., 2022).

Figure 4. Enrichment of viable iBM cells with an anti-NaPi2b antibody
(A) *SLC34A2* expression in *DUX4*-pulsed hESCs in each cluster. Expression levels are shown as log-normalized UMI counts.
(B) *SLC34A2* expression in human preimplantation embryo and ESCs (Yan et al., 2013). Expression levels are shown as log fragments per kilobase of transcript per million mapped reads (FPKM) values. The horizontal bars in each violin plot indicate the median expression level per cluster (A) or stage (B).
(C) Immunocytochemical detection of DUX4 and NaPi2b in *DUX4*-pulsed hESCs at 6 h. DAPI (blue) was used as nuclear counterstain. Scale bars, 20 μm.
(D) Schematic illustration of the cell-sorting procedure.
(E) Flow cytometric analysis showing the proportion of NaPi2b$^+$ cells (yellow dots). Representative data from two independent experiments are shown. FSC, forward scatter; 2ndAb ctrl, secondary antibody control.
(F) DUX4 target gene expression in NaPi2b$^+$ and NaPi2b$^-$ cells cultured for 6 h after cell sorting. Error bars represent the SEM of two independent experiments.
(G) Multidimensional scaling analysis integrating the sorted NaPi2b$^+$ and NaPi2b$^-$ cells, unsorted *DUX4*-pulsed hESCs (dox unsorted), no induction (no dox), and H9 primed and naive ESCs (n = 3 independent experiments), with the single-cell RNA-seq data of HS980 primed ESCs (n = 16 cells) and eight-cell stage embryo (n = 13 cells) from Jouhilahti et al. (2016).
(H) Heatmap showing the expression scores of stage-specific genes. Comp.morula, compacted morula; ICM, inner cell mass.
See also Figure S4.

The iBM cells can become a powerful tool to study the roles of specific genes in the context of the EGA, without the need for early human embryos that is ethically questioned and that are available in limited numbers where allowed. We envision further experiments where single EGA-associated genes can be inactivated by gene editing in hESCs and subsequently induced toward iBM using methods described here. Comparison of such cells to iBM cells by scRNA-seq may illuminate each gene's functional role and possible redundancies in the EGA transcriptome.

There are some limitations in our study. Our transcriptomic comparison with cleavage-stage embryos showed that EGA genes were efficiently activated by a transient *DUX4* induction. However, many oocyte-specific genes, which remain in the eight-cell stage blastomeres, were absent. Therefore, the iBM cells do not completely mimic the transcriptome of the embryonic blastomere cells. The relevance of these oocyte-specific factors for modeling early embryo behavior with stem cells remains to be determined. Our time-series analysis demonstrated that the iBM cell transcriptome reverted to the original ESC state within 48 h after *DUX4* induction, suggesting that the iBM cells could not be maintained under the culture condition optimal for primed hESCs. Identification of the critical signaling pathways that drive the differentiation of the iBM cells would allow further optimization of the conditions aiming at stable iBM cell cultures. A recent study succeeded in converting human PSCs into eight-cell-like cells with a combination of chemical treatments (Mazid et al., 2022). Another study established stable totipotent-like stem cells from mESCs by chemical induction (Yang et al., 2022). These reported chemicals might be useful for the optimization of the prolonged culture of the iBM cells. Feasibility of the iBM cells as embryo model requires further functional validation, such as directed differentiation assays.

EXPERIMENTAL PROCEDURES

Additional methods and more detailed descriptions of STRT RNA-seq and scRNA-seq can be found in the supplemental experimental procedures.

Cell culture

DUX4-TetOn hESCs (Vuoristo et al., 2022) were maintained on hESC-qualified Geltrex (Thermo Fisher Scientific) coated tissue culture dishes in Essential 8 culture medium (Thermo Fisher Scientific) in 5% CO_2 at 37°C. The cells were passaged every 3–5 days after a 3-min incubation with 0.5 mM EDTA (Thermo Fisher Scientific). For the cell growth assays, the cells were imaged with the Incucyte S3 analysis system (Sartorius). Naive H9 hESCs, which had been previously converted from primed to naive stem cell stage using the NaïveCult Induction kit (STEMCELL Technologies), were cultured on irradiated mouse embryonic fibroblast (MEF) feeders (Gibco) in the NaïveCult Expansion Medium (STEMCELL Technologies) in 5% O_2/5% CO_2 at 37°C. Naive hESCs were dissociated with TrypLE Express (Thermo Fisher Scientific) every 3–5 days and re-plated on MEF feeders, prepared a day before hESC seeding. The cell culture medium was supplemented with 10 μM ROCKi Y-27632 (Selleckchem) for the first 24 h post-naive hESC plating.

Doxycycline pulsing on DUX4-TetOn hESCs

DUX4-TetOn hESCs were incubated with 1 μg/mL of doxycycline in Essential 8 culture medium in 5% CO_2 incubator at 37°C for varied times as indicated. After the doxycycline induction, the DUX4-TetOn hESCs were washed three times with Essential 8 culture medium and incubated thereafter in Essential 8 medium for the indicated times.

RNA extraction and quantitative real-time PCR

Total RNA was isolated using NucleoSpin RNA kit (Macherey Nagel) according to the manufacturer's protocol. For cDNA synthesis, 500 ng of total RNA was reverse-transcribed by MMLV-RTase (Promega) with oligo dT priming. The resulting cDNA was used as a template for quantitative real-time PCR using 5× HOT FIREPol qPCR Mix (Solis BioDyne) on the LightCycler 96 System (Roche). Relative expression values were calculated with the $2^{-\Delta\Delta Ct}$ method (Livak and Schmittgen, 2001), using cyclophilin G (*PPIG*) as an internal control, normalized against the untreated cells (Figure S2C) or NaPi2b^{-} cells (Figure 4F).

STRT whole-culture RNA-seq library preparation and sequencing

Doxycycline-induced and control DUX4-TetOn hESCs were collected for STRT whole-culture RNA-seq immediately, 24 h, and 48 h after 15 min or 30 min of doxycycline or without doxycycline treatment. FACS-sorted cells were collected from three independent experiments as described later. Naive H9 hESCs were collected by hand picking the colonies from the cell culture dishes, using sterile needles. Conventional primed H9 hESCs were collected by washing the cells once with PBS and lysing the culture according to the NucleoSpin RNA kit protocol. We used 16–20 ng of RNA to generate a 48-plex RNA-seq library using a modified STRT method with unique molecular identifiers (UMIs) (Ezer et al., 2021; Islam et al., 2011, 2014). Briefly, RNA samples were placed in a 48-well plate, and a universal primer, template-switching oligo-nucleotides, and a well-specific 6-bp barcode sequence (for sample identification) were added to each well (Katayama et al., 2013; Krjutškov et al., 2016). We pooled the synthesized cDNAs into one library, performed fragmentation to 200–400 bp using an M220 Focused-ultrasonicator (Covaris), captured the 5′ fragments, added an adapter, and amplified the targets by PCR. The RNA-seq library was sequenced with Illumina NextSeq 500 System, High Output mode.

scRNA-seq library preparation and sequencing

DUX4-TetOn hESCs were seeded into three plates at each experiment, two doxycycline-treated and one untreated, and then collected at 6, 12, 24, and 48 h after treatment. The cells were washed once with PBS and incubated with TrypLE Express for

4 min. TrypLE was diluted with Essential 8 medium, and the cell suspensions were filtered through 40 μm Cell Strainers. Cell suspensions were centrifuged at 400 rcf for 8 min. Cell pellets were resuspended each in 100 μL of Dead Cell Removal Kit microbeads (Miltenyi Biotec) and incubated at room temperature for 15 min. After incubation, each cell-microbead suspension was gently resuspended to 800 μL of freshly prepared 1× binding buffer. Cell suspensions were pipetted to magnetic MS columns (Miltenyi Biotec) 500 μL at a time and let flow through. The columns were washed three times with 1× binding buffer. The cell suspensions were centrifuged at 400 rcf for 5 min, and the pellets were resuspended each in 400 μL of 10x Genomics sample buffer. The cells were counted, and the volumes were adjusted to approximately 1,200 cells/μL of suspension. The samples were kept on ice prior to analysis of cell quality and number and preparation of the scRNA sequencing libraries. Approximately 94% of the nucleated cells were alive. The libraries were prepared using Chromium Next GEM Single Cell 3′ Gene Expression v.3.1 chemistry and sequencing was performed using Illumina NovaSeq 6000 system at the Institute for Molecular Medicine Finland (FIMM) Single-Cell Analytics unit.

Immunocytochemical staining of DUX4-TetOn hESCs

Cells were fixed on Ibidi eight-well μ slides with 3.8% paraformaldehyde at room temperature for 15 min and washed three times with PBS. For the nuclear epitopes, the cells were permeabilized using 0.5% Triton X-100-PBS at room temperature for 7 min. The cells were washed once with PBS, and unspecific binding of antibodies was blocked by Ultravision Protein Block solution (Thermo Fisher Scientific) by a 10-min incubation at room temperature. Primary antibodies were diluted in washing buffer (0.1% Tween20-PBS) and incubated at 4°C overnight. Excess primary antibody solutions were removed, and the cells were washed three times with washing buffer. The secondary antibodies were diluted 1:1,000 in washing buffer and incubated at room temperature for 30 min. The samples were washed three times with washing buffer, and nuclei were counterstained with DAPI, diluted 1:1,000 in washing buffer. The samples were washed once and kept in PBS for imaging. Protocols of western blotting and annexin V staining are provided in supplemental experimental procedures. All the antibodies used in this study are listed in the key resources table.

Confocal microscopy and image analysis

Images were captured with a Leica TCS SP8 confocal laser scanning microscope (Leica Microsystems, Mannheim, Germany) using Leica HC PL APO CS2 40×/1.10NA water objective and 1,024 × 1,024 scan format. For annexin V stainings, the cells were imaged with a Leica TCS SP8 X confocal microscope with white laser. The images were captured with either 20× air objective or 63× oil objective using 1,024 × 1,024 scan format. The data were processed using Fiji (http://fiji.sc; Schindelin et al., 2012). The images were softened using Gaussian filter (radius = 1-pixel kernel). Fluorescence intensity was quantified using Fiji by segmenting the regions of interest with the Otsu thresholding method (Otsu, 1979). The mean fluorescence intensities of POU5F1 staining were compared between LEUTX-positive (intensity $\geq$ 10) and negative (intensity < 10) cells.

FACS of DUX4-TetOn hESCs

The DUX4-TetOn hESCs were washed once with PBS and incubated with TrypLE Express for 4 min in 5% CO_2 incubator at 37°C. The TrypLE Express was diluted with cold FACS buffer (5% fetal bovine serum in PBS supplemented with 10 μM ROCK inhibitor Y-27632), and the cell suspensions were let flow through 40 μm Cell Strainers. The cells were counted, and approximately 5×10^5 cells were aliquoted per Eppendorf tube. From here, on the cells were kept on ice. The cells were centrifuged at 4°C, 300 rcf for 5 min. The primary anti-NaPi2b antibody, mouse MX35, a kind gift from Dr. Gerd Ritter, was diluted 1:100 (final concentration 20 μg/mL) in FACS buffer. The cells were incubated for 1 h on ice for primary antibody staining (MX35). The samples were washed three times with FACS buffer by centrifugation as above. Secondary antibody Alexa-Fluor-594-conjugated donkey anti-mouse (A-21203, Thermo Fisher Scientific), was diluted 1:1,000 in FACS buffer and incubated with cells on ice for 30 min. The cells were washed three times as above. The cells were analyzed and separated using Sony SH800Z Cell Sorter (Sony Biotechnology), using 100 μm nozzle. Altogether 5×10^5 cells were collected for follow-up culture. The cells were centrifuged at 4°C, 300 rcf for 5 min, resuspended in Essential 8 culture medium with 10 μM ROCK inhibitor, and cultured for 6 h in 5% CO_2, at 37°C, prior to cell lysis for RNA isolation.

STRT RNA-seq data processing

The sequenced STRT RNA-seq raw reads were processed as described elsewhere (https://github.com/my0916/STRT2; Ezer et al., 2021). Two samples collected immediately after induction were excluded due to a low number of mapped reads. The STRT RNA-seq data of continuous *DUX4* induction, treated by doxycycline for 4 h, were obtained from Vuoristo et al. (2022) and reprocessed as described elsewhere (https://github.com/my0916/STRT2; Ezer et al., 2021). The list of EGA genes was retrieved from Töhönen et al. (2015; Table S1), and TFEs overlapped with these gene regions were analyzed further. The list of DUX4 target genes expressed in the cleavage-stage human embryo was retrieved from Resnick et al. (2019; Table S1). The list of stage-specific genes was retrieved from Stirparo et al. (2018). The STRT RNA-seq data of HS980 primed ESCs and eight-cell stage cells were obtained from Jouhilahti et al. (2016). Detailed analysis methods are provided in the supplemental experimental procedures.

scRNA-seq data processing

The raw BCL files were demultiplexed and converted to FASTQ files with Cell Ranger (10x Genomics, v.3.1.0) mkfastq and mapped against the customized human reference genome (GRCh38 with DUX4-IRES-EmGFP) with STAR (Dobin et al., 2013). The cellranger aggr pipeline was used to combine all the data to generate a gene-count matrix. The output count data were subsequently analyzed with the R package Seurat (v.4.0.0) (Hao et al., 2021). To measure the expression of *DUX4*, we quantified the expression of DUX4-IRES-EmGFP to avoid problems of mapping to the D4Z4 repeat locus. Gene expression scores of each signature were calculated using the gene signature scoring function retrieved from Liu et al. (2020). The list of EGA genes was obtained from Töhönen et al. (2015), and that of signature genes of primed and naive PSCs was obtained

from Liu et al. (2020). The list of eight-cell and ESC genes was retrieved from Jouhilahti et al. (2016; Table S1). The list of 299 LEUTX-target genes was retrieved from our STRT RNA-seq data of *LEUTX*-inducible hESCs (L.G., E.-M.J., M.Y., Fei Liangru, J.W., Tomi T. Airenne, R.T., Shruti Bhagat, M.H.T., Yasuhiro Murakawa, Kari Salokas, Xiaonan Liu, Sini Miettinen, S.V., Thomas R. Bürglin, Biswajyoti Sahu, T.O., Mark S. Johnson, S.K., M.V., J.K., unpublished data; Table S1). Cell type annotation was conducted with the R package SingleR (v.1.4.1) (Aran et al., 2019), using the scRNA-seq data of human preimplantation embryos and ESCs (Yan et al., 2013) as the reference data. Pseudotime trajectory analysis was performed using the R package Monocle (v.2.18.0) (Qiu et al., 2017). scRNA-seq data of human preimplantation embryos (Petropoulos et al., 2016) and naive and primed hESCs (Messmer et al., 2019) were obtained from the ArrayExpress database with the accession number E-MTAB-3929 and E-MTAB-6819, respectively. These data were processed and integrated with our scRNA-seq dataset of *DUX4*-pulsed hESCs using the FindIntegrationAnchors and IntegrateData functions in Seurat. Our cells were randomly downsampled to 400 cells per cluster so that the number of cells was comparable between different datasets. Detailed analysis methods are provided in the supplemental experimental procedures.

Data and code availability

The STRT whole-culture RNA-seq and scRNA-seq data of DUX4-TetOn hESCs used in this study have been deposited in the ArrayExpress database at EMBL-EBI and are available under the accession codes "E-MTAB-10569" and "E-MTAB-10581," respectively.

SUPPLEMENTAL INFORMATION

Supplemental information can be found online at https://doi.org/10.1016/j.stemcr.2022.06.002.

AUTHOR CONTRIBUTIONS

Conceptualization, M.Y., S.V., and J.K.; data curation, M.Y.; formal analysis, M.Y. and J.S.; funding acquisition, M.Y., T.O., S.V., and J.K.; investigation, M.Y., I.K., S.N., J.S., K.L., L.G., E.-M.J., M.V., M.H.T., and S.V.; methodology, M.Y., J.W., R.T., and S.V.; project administration, J.K.; resources, R.T., S.K., and S.V.; software, M.Y. and S.K.; supervision, T.O., R.T., S.K., S.V., and J.K.; validation, M.Y., I.K., S.N., and S.V.; visualization, M.Y. and S.V.; writing – original draft, M.Y. and S.V.; writing – review & editing, all authors.

ACKNOWLEDGMENTS

The authors thank Dr. Gerd Ritter (Ludwig Institute for Cancer Research, New York City, USA) for kindly providing anti-NaPi2b monoclonal antibody (MX35). The STRT whole-culture RNA-seq and scRNA-seq were carried out at the Biomedicum Functional Genomics Unit (FuGU), University of Helsinki and FIMM Single-Cell Analytics unit supported by HiLIFE and Biocenter Finland, respectively. The flow cytometry analyses and FACS were performed at the HiLIFE Flow Cytometry Unit, and the confocal imaging was performed at the HiLIFE Biomedicum Imaging Unit, both at the University of Helsinki. The live-cell imaging was performed at the Biomedicum Stem Cell Center, the University of Helsinki, supported by HiLIFE and Biocenter Finland. Most of the computations for this work were performed on resources provided by the Swedish National Infrastructure for Computing (SNIC) through the Uppsala Multidisciplinary Center for Advanced Computational Science (UPPMAX) under project SNIC 2017/7-317. M.Y. was supported by Scandinavia-Japan Sasakawa Foundation, Japan Eye Bank Association, Astellas Foundation for Research on Metabolic Disorders, and Japan Society for the Promotion of Science (JSPS) Overseas Research Fellowships. S.N. was supported by the University of Helsinki Doctoral Program in Biomedicine. S.V. lab was supported by the research project grants from the Sigrid Jusélius Foundation and the University of Helsinki. Work in the J.K. lab was supported by Jane and Aatos Erkko Foundation, Sigrid Jusélius Foundation, Swedish Research Council, the Finnish Medical Society Finska Läkaresällskapet, and Föreningen Livoch Hälsa. Work in the T.O. lab was supported by Jane and Aatos Erkko Foundation and Helsinki University Hospital funds. J.W. was supported by Sigrid Jusélius Foundation and Päivikki and Sakari Sohlberg Foundation. S.K. was supported by Jane and Aatos Erkko Foundation. The preprint of this work was first deposited on bioRxiv (https://doi.org/10.1101/2021.08.25.457357) on August 27, 2021.

CONFLICTS OF INTERESTS

The authors declare no competing interests.

Received: May 24, 2022
Revised: June 2, 2022
Accepted: June 2, 2022
Published: June 30, 2022

REFERENCES

Aran, D., Looney, A.P., Liu, L., Wu, E., Fong, V., Hsu, A., Chak, S., Naikawadi, R.P., Wolters, P.J., Abate, A.R., et al. (2019). Reference-based analysis of lung single-cell sequencing reveals a transitional profibrotic macrophage. Nat. Immunol. *20*, 163–172.

Bosnakovski, D., Xu, Z., Gang, E.J., Galindo, C.L., Liu, M., Simsek, T., Garner, H.R., Agha-Mohammadi, S., Tassin, A., Coppée, F., et al. (2008). An isogenetic myoblast expression screen identifies DUX4-mediated FSHD-associated molecular pathologies. EMBO J. *27*, 2766–2779.

Braude, P., Bolton, V., and Moore, S. (1988). Human gene expression first occurs between the four- and eight-cell stages of preimplantation development. Nature *332*, 459–461.

De Iaco, A., Planet, E., Coluccio, A., Verp, S., Duc, J., and Trono, D. (2017). DUX-family transcription factors regulate zygotic genome activation in placental mammals. Nat. Genet. *49*, 941–945.

De Iaco, A., Coudray, A., Duc, J., and Trono, D. (2019). DPPA2 and DPPA4 are necessary to establish a 2C-like state in mouse embryonic stem cells. EMBO Rep. *20*, e47382.

Dobin, A., Davis, C.A., Schlesinger, F., Drenkow, J., Zaleski, C., Jha, S., Batut, P., Chaisson, M., and Gingeras, T.R. (2013). STAR: ultrafast universal RNA-seq aligner. Bioinformatics *29*, 15–21.

Ezer, S., Yoshihara, M., Katayama, S., Daub, C., Lohi, H., Krjutskov, K., and Kere, J. (2021). Generation of RNA sequencing libraries for

transcriptome analysis of globin-rich tissues of the domestic dog. STAR Protoc. *2*, 100995.

Fu, X., Wu, X., Djekidel, M.N., and Zhang, Y. (2019). Myc and Dnmt1 impede the pluripotent to totipotent state transition in embryonic stem cells. Nat. Cell Biol. *21*, 835–844.

Fu, X., Djekidel, M.N., and Zhang, Y. (2020). A transcriptional roadmap for 2C-like-to-pluripotent state transition. Sci. Adv. *6*, eaay5181.

Genet, M., and Torres-Padilla, M.E. (2020). The molecular and cellular features of 2-cell-like cells: a reference guide. Development *147*, dev189688.

Geng, L.N., Yao, Z., Snider, L., Fong, A.P., Cech, J.N., Young, J.M., van der Maarel, S.M., Ruzzo, W.L., Gentleman, R.C., Tawil, R., et al. (2012). DUX4 activates germline genes, retroelements, and immune mediators: implications for facioscapulohumeral dystrophy. Dev. Cell *22*, 38–51.

Hao, Y., Hao, S., Andersen-Nissen, E., Mauck, W.M., 3rd, Zheng, S., Butler, A., Lee, M.J., Wilk, A.J., Darby, C., Zager, M., et al. (2021). Integrated analysis of multimodal single-cell data. Cell *184*, 3573–3587.e29.

Hendrickson, P.G., Doráis, J.A., Grow, E.J., Whiddon, J.L., Lim, J.W., Wike, C.L., Weaver, B.D., Pflueger, C., Emery, B.R., Wilcox, A.L., et al. (2017). Conserved roles of mouse DUX and human DUX4 in activating cleavage-stage genes and MERVL/HERVL retrotransposons. Nat. Genet. *49*, 925–934.

Islam, S., Zeisel, A., Joost, S., La Manno, G., Zajac, P., Kasper, M., Lönnerberg, P., and Linnarsson, S. (2014). Quantitative single-cell RNA-seq with unique molecular identifiers. Nat. Methods *11*, 163–166.

Islam, S., Kjällquist, U., Moliner, A., Zajac, P., Fan, J.B., Lönnerberg, P., and Linnarsson, S. (2011). Characterization of the single-cell transcriptional landscape by highly multiplex RNA-seq. Genome Res. *21*, 1160–1167.

Jouhilahti, E.M., Madissoon, E., Vesterlund, L., Töhönen, V., Krjutškov, K., Plaza Reyes, A., Petropoulos, S., Månsson, R., Linnarsson, S., Bürglin, T., et al. (2016). The human PRD-like homeobox gene LEUTX has a central role in embryo genome activation. Development *143*, 3459–3469.

Jukam, D., Shariati, S.A.M., and Skotheim, J.M. (2017). Zygotic genome activation in vertebrates. Dev. Cell *42*, 316–332.

Katayama, S., Töhönen, V., Linnarsson, S., and Kere, J. (2013). SAMstrt: statistical test for differential expression in single-cell transcriptome with spike-in normalization. Bioinformatics *29*, 2943–2945.

Kowaljow, V., Marcowycz, A., Ansseau, E., Conde, C.B., Sauvage, S., Mattéotti, C., Arias, C., Corona, E.D., Nuñez, N.G., Leo, O., et al. (2007). The DUX4 gene at the FSHD1A locus encodes a pro-apoptotic protein. Neuromuscul. Disord. *17*, 611–623.

Krjutškov, K., Katayama, S., Saare, M., Vera-Rodriguez, M., Lubenets, D., Samuel, K., Laisk-Podar, T., Teder, H., Einarsdottir, E., Salumets, A., et al. (2016). Single-cell transcriptome analysis of endometrial tissue. Hum. Reprod. *31*, 844–853.

Liu, X., Nefzger, C.M., Rossello, F.J., Chen, J., Knaupp, A.S., Firas, J., Ford, E., Pflueger, J., Paynter, J.M., Chy, H.S., et al. (2017). Comprehensive characterization of distinct states of human naive pluripotency generated by reprogramming. Nat. Methods *14*, 1055–1062.

Liu, X., Ouyang, J.F., Rossello, F.J., Tan, J.P., Davidson, K.C., Valdes, D.S., Schröder, J., Sun, Y.B.Y., Chen, J., Knaupp, A.S., et al. (2020). Reprogramming roadmap reveals route to human induced trophoblast stem cells. Nature *586*, 101–107.

Livak, K.J., and Schmittgen, T.D. (2001). Analysis of relative gene expression data using real-time quantitative PCR and the 2(-Delta Delta C(T)) Method. Methods *25*, 402–408.

Macfarlan, T.S., Gifford, W.D., Driscoll, S., Lettieri, K., Rowe, H.M., Bonanomi, D., Firth, A., Singer, O., Trono, D., and Pfaff, S.L. (2012). Embryonic stem cell potency fluctuates with endogenous retrovirus activity. Nature *487*, 57–63.

Mazid, M.A., Ward, C., Luo, Z., Liu, C., Li, Y., Lai, Y., Wu, L., Li, J., Jia, W., Jiang, Y., et al. (2022). Rolling back human pluripotent stem cells to an eight-cell embryo-like stage. Nature *605*, 315–324.

Messmer, T., von Meyenn, F., Savino, A., Santos, F., Mohammed, H., Lun, A.T.L., Marioni, J.C., and Reik, W. (2019). Transcriptional heterogeneity in naive and primed human pluripotent stem cells at single-cell resolution. Cell Rep. *26*, 815–824.e4.

Otsu, N. (1979). A threshold selection method from gray-level histograms. IEEE Trans. Syst. Man Cybern. *9*, 62–66.

Petropoulos, S., Edsgärd, D., Reinius, B., Deng, Q., Panula, S.P., Codeluppi, S., Plaza Reyes, A., Linnarsson, S., Sandberg, R., and Lanner, F. (2016). Single-cell RNA-seq reveals lineage and X chromosome dynamics in human preimplantation embryos. Cell *165*, 1012–1026.

Qiu, X., Mao, Q., Tang, Y., Wang, L., Chawla, R., Pliner, H.A., and Trapnell, C. (2017). Reversed graph embedding resolves complex single-cell trajectories. Nat. Methods *14*, 979–982.

Resnick, R., Wong, C.J., Hamm, D.C., Bennett, S.R., Skene, P.J., Hake, S.B., Henikoff, S., van der Maarel, S.M., and Tapscott, S.J. (2019). DUX4-Induced histone variants H3.X and H3.Y mark DUX4 target genes for expression. Cell Rep. *29*, 1812–1820.e5.

Rickard, A.M., Petek, L.M., and Miller, D.G. (2015). Endogenous DUX4 expression in FSHD myotubes is sufficient to cause cell death and disrupts RNA splicing and cell migration pathways. Hum. Mol. Genet. *24*, 5901–5914.

Rodriguez-Terrones, D., Gaume, X., Ishiuchi, T., Weiss, A., Kopp, A., Kruse, K., Penning, A., Vaquerizas, J.M., Brino, L., and Torres-Padilla, M.E. (2018). A molecular roadmap for the emergence of early-embryonic-like cells in culture. Nat. Genet. *50*, 106–119.

Schindelin, J., Arganda-Carreras, I., Frise, E., Kaynig, V., Longair, M., Pietzsch, T., Preibisch, S., Rueden, C., Saalfeld, S., Schmid, B., et al. (2012). Fiji: an open-source platform for biological-image analysis. Nat. Methods *9*, 676–682.

Schlaeger, T.M., Daheron, L., Brickler, T.R., Entwisle, S., Chan, K., Cianci, A., DeVine, A., Ettenger, A., Fitzgerald, K., Godfrey, M., et al. (2015). A comparison of non-integrating reprogramming methods. Nat. Biotechnol. *33*, 58–63.

Shadle, S.C., Zhong, J.W., Campbell, A.E., Conerly, M.L., Jagannathan, S., Wong, C.J., Morello, T.D., van der Maarel, S.M., and Tapscott, S.J. (2017). DUX4-induced dsRNA and MYC mRNA stabilization activate apoptotic pathways in human cell models of facioscapulohumeral dystrophy. PLoS Genet. *13*, e1006658.

Stirparo, G.G., Boroviak, T., Guo, G., Nichols, J., Smith, A., and Bertone, P. (2018). Integrated analysis of single-cell embryo data yields a unified transcriptome signature for the human pre-implantation epiblast. Development *145*, dev158501.

Taubenschmid-Stowers, J., Rostovskaya, M., Santos, F., Ljung, S., Argelaguet, R., Krueger, F., Nichols, J., and Reik, W. (2022). 8C-like cells capture the human zygotic genome activation program in vitro. Cell Stem Cell *29*, 449–459.e6.

Töhönen, V., Katayama, S., Vesterlund, L., Jouhilahti, E.M., Sheikhi, M., Madissoon, E., Filippini-Cattaneo, G., Jaconi, M., Johnsson, A., Bürglin, T.R., et al. (2015). Novel PRD-like homeodomain transcription factors and retrotransposon elements in early human development. Nat. Commun. *6*, 8207.

Töhönen, V., Katayama, S., Vesterlund, L., Sheikhi, M., Antonsson, L., Filippini-Cattaneo, G., Jaconi, M., Johnsson, A., Linnarsson, S., Hovatta, O., et al. (2017). Transcription activation of early human development suggests DUX4 as an embryonic regulator. Preprint at bioRxiv. https://doi.org/10.1101/123208.

Vuoristo, S., Bhagat, S., Hydén-Granskog, C., Yoshihara, M., Gawriyski, L., Jouhilahti, E.M., Ranga, V., Tamirat, M., Huhtala, M., Kirjanov, I., et al. (2022). DUX4 is a multifunctional factor priming human embryonic genome activation. iScience *25*, 104137.

Wallace, L.M., Garwick, S.E., Mei, W., Belayew, A., Coppee, F., Ladner, K.J., Guttridge, D., Yang, J., and Harper, S.Q. (2011). DUX4, a candidate gene for facioscapulohumeral muscular dystrophy, causes p53-dependent myopathy in vivo. Ann. Neurol. *69*, 540–552.

Whiddon, J.L., Langford, A.T., Wong, C.J., Zhong, J.W., and Tapscott, S.J. (2017). Conservation and innovation in the DUX4-family gene network. Nat. Genet. *49*, 935–940.

Yan, L., Yang, M., Guo, H., Yang, L., Wu, J., Li, R., Liu, P., Lian, Y., Zheng, X., Yan, J., et al. (2013). Single-cell RNA-Seq profiling of human preimplantation embryos and embryonic stem cells. Nat. Struct. Mol. Biol. *20*, 1131–1139.

Yang, F., Huang, X., Zang, R., Chen, J., Fidalgo, M., Sanchez-Priego, C., Yang, J., Caichen, A., Ma, F., Macfarlan, T., et al. (2020). DUX-miR-344-ZMYM2-Mediated activation of MERVL LTRs induces a totipotent 2C-like state. Cell Stem Cell *26*, 234–250.e7.

Yang, M., Yu, H., Yu, X., Liang, S., Hu, Y., Luo, Y., Izsvák, Z., Sun, C., and Wang, J. (2022). Chemical-induced chromatin remodeling reprograms mouse ESCs to totipotent-like stem cells. Cell Stem Cell *29*, 400–418.e13.

Yin, B.W., Kiyamova, R., Chua, R., Caballero, O.L., Gout, I., Gryshkova, V., Bhaskaran, N., Souchelnytskyi, S., Hellman, U., Filonenko, V., et al. (2008). Monoclonal antibody MX35 detects the membrane transporter NaPi2b (SLC34A2) in human carcinomas. Cancer Immun. *8*, 3.

Young, J.M., Whiddon, J.L., Yao, Z., Kasinathan, B., Snider, L., Geng, L.N., Balog, J., Tawil, R., van der Maarel, S.M., and Tapscott, S.J. (2013). DUX4 binding to retroelements creates promoters that are active in FSHD muscle and testis. PLoS Genet. *9*, e1003947.

Stem Cell Reports, Volume *17*

Supplemental Information

Transient *DUX4* expression in human embryonic stem cells induces blastomere-like expression program that is marked by SLC34A2

Masahito Yoshihara, Ida Kirjanov, Sonja Nykänen, Joonas Sokka, Jere Weltner, Karolina Lundin, Lisa Gawriyski, Eeva-Mari Jouhilahti, Markku Varjosalo, Mari H. Tervaniemi, Timo Otonkoski, Ras Trokovic, Shintaro Katayama, Sanna Vuoristo, and Juha Kere

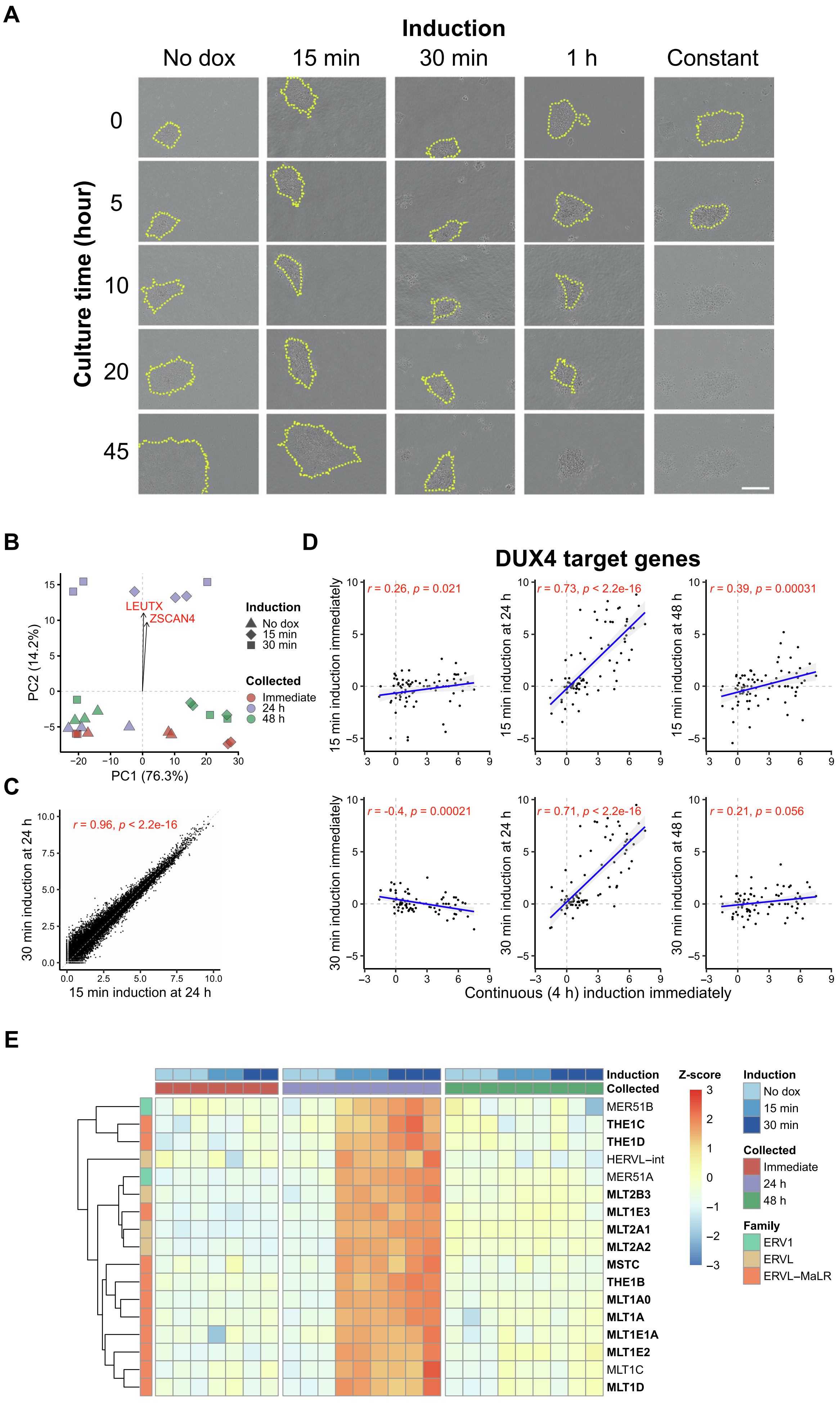
A
Induction
No dox
15 min
30 min
1 h
Constant
Culture time (hour)
0
5
10
20
45
B
PC2 (14.2%)
PC1 (76.3%)
LEUTX
ZSCAN4
Induction
No dox
15 min
30 min
Collected
Immediate
24 h
48 h
C
30 min induction at 24 h
15 min induction at 24 h
r = 0.96, p < 2.2e-16
D
DUX4 target genes
15 min induction immediately
15 min induction at 24 h
15 min induction at 48 h
30 min induction immediately
30 min induction at 24 h
30 min induction at 48 h
r = 0.26, p = 0.021
r = 0.73, p < 2.2e-16
r = 0.39, p = 0.00031
r = -0.4, p = 0.00021
r = 0.71, p < 2.2e-16
r = 0.21, p = 0.056
Continuous (4 h) induction immediately
E
Induction
Collected
MER51B
THE1C
THE1D
HERVL–int
MER51A
MLT2B3
MLT1E3
MLT2A1
MLT2A2
MSTC
THE1B
MLT1A0
MLT1A
MLT1E1A
MLT1E2
MLT1C
MLT1D
Z-score
Induction
No dox
15 min
30 min
Collected
Immediate
24 h
48 h
Family
ERV1
ERVL
ERVL–MaLR

Figure S1. Effect of transient *DUX4* induction in hESCs, related to Figure 1

(A) Bright field microscopy images of DUX4-TetOn hESCs after varied times of doxycycline induction. Living cells used for the measurement of colony size are surrounded by yellow lines. Scale bars, 200 μm.

(B) Principal component analysis of the STRT whole-culture RNA-seq data. Arrows show the variables of *LEUTX* and *ZSCAN4* on PC1 and PC2.

(C) Correlation of gene expression profiles of DUX4-TetOn hESCs at 24 h after 15 min (x-axis) and 30 min (y-axis) of induction. Expression levels are shown as log normalized counts.

(D) Transcriptional changes of 80 DUX4 target genes after continuous *DUX4* induction (x-axis) and transient *DUX4* induction (y-axis). Axes show the $\log_2$ fold expression changes over no *DUX4* induction. The correlation coefficients (r) and *P*-values were calculated using a two-sided Spearman's correlation test. The linear regression line (blue) and 95% confidence interval (gray shaded) are shown. See also Table S1.

(E) Heatmap showing the expression of repetitive elements. Repetitive elements significantly upregulated at 24 h after 15 min and 30 min of induction are shown. Elements bound by DUX4 (Young et al., 2013) are shown in bold.

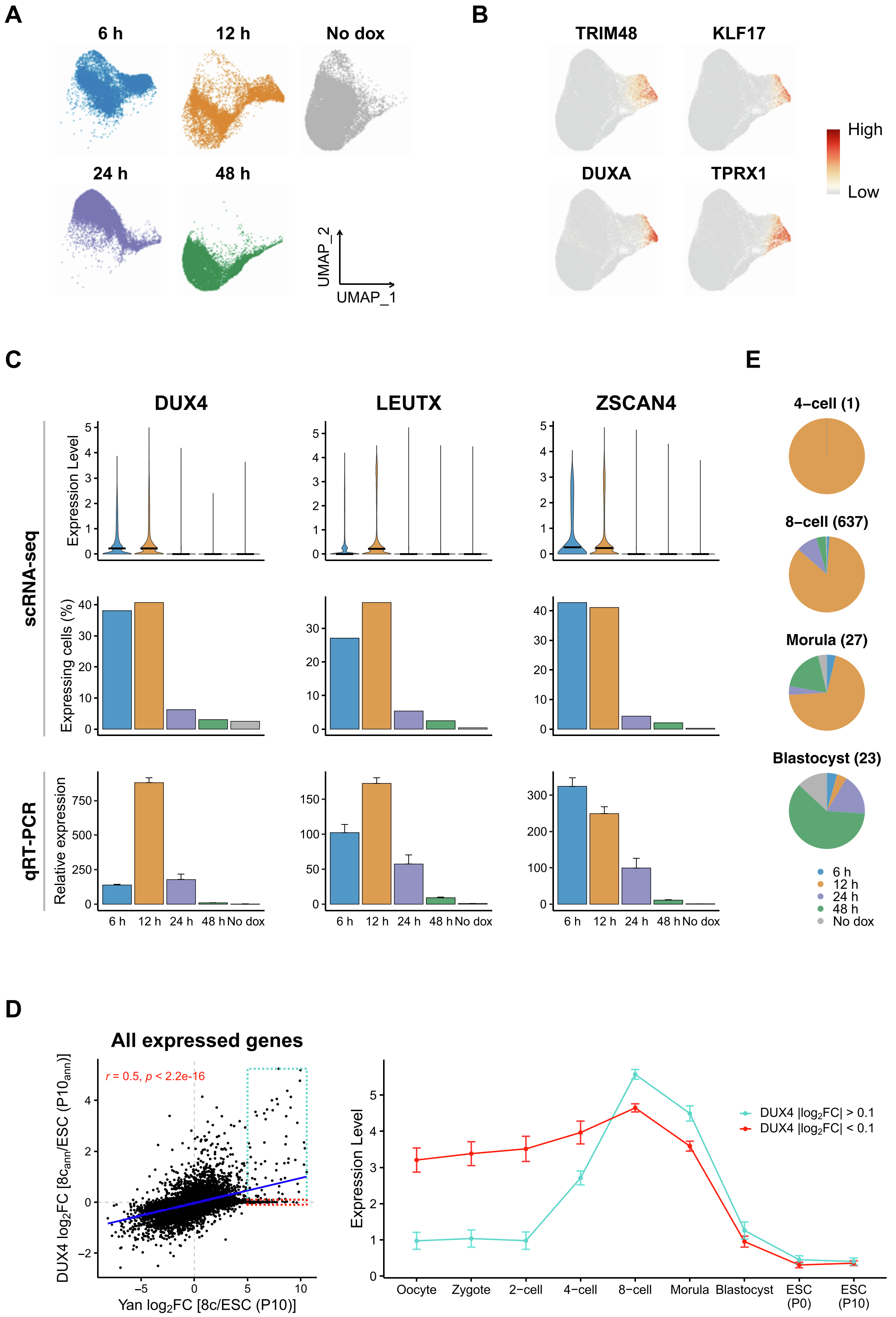
A
6 h
12 h
No dox
24 h
48 h
UMAP_2
UMAP_1
B
TRIM48
KLF17
DUXA
TPRX1
High
Low
C
DUX4
LEUTX
ZSCAN4
scRNA-seq
Expression Level
Expressing cells (%)
qRT-PCR
Relative expression
6 h 12 h 24 h 48 h No dox
E
4−cell (1)
8−cell (637)
Morula (27)
Blastocyst (23)
6 h
12 h
24 h
48 h
No dox
D
All expressed genes
r = 0.5, p < 2.2e-16
DUX4 log2FC [8cann/ESC (P10ann)]
Yan log2FC [8c/ESC (P10)]
Expression Level
Oocyte
Zygote
2−cell
4−cell
8−cell
Morula
Blastocyst
ESC (P0)
ESC (P10)
DUX4 |log2FC| > 0.1
DUX4 |log2FC| < 0.1

Figure S2. Time-course analysis of *DUX4*-pulsed hESCs at single-cell level, related to Figure 2

(A) UMAP plot colored by collected time points. Note that there are few untreated (No dox) cells in the rightmost cluster.

(B) Expression pattern of DUX4 target genes projected onto the UMAP plot.

(C) Expression of *DUX4* and its target genes by collected time points. Top: expression levels shown as log normalized UMI counts. Middle: proportion of expressing cells (UMI count > 1). Bottom: expression in whole-culture cells measured by quantitative real-time (qRT)-PCR. Error bars represent the SEM of three different culture plates.

(D) Transcriptional changes of 15,902 genes in actual eight-cell stage cells (x-axis) and *DUX4*-pulsed hESCs annotated as eight-cell stage cells (y-axis) compared with hESCs. Axes show the $\log_2$ fold expression changes over hESCs (P10; x-axis) or cells annotated as hESCs (P10; y-axis). $8c_{ann}$, cells annotated as eight-cell stage cells; ESC ($P10_{ann}$), cells annotated as ESC (P10). Right panel shows the mean expression (log FPKM) during early development of 64 differentially expressed genes ($|\log_2 FC| > 0.1$; lightblue) and 72 unchanged genes ($|\log_2 FC| < 0.1$; red) by *DUX4* induction. Error bars denote SEM.

(E) Proportion of collected time points in cells annotated as early embryonic stage cells.

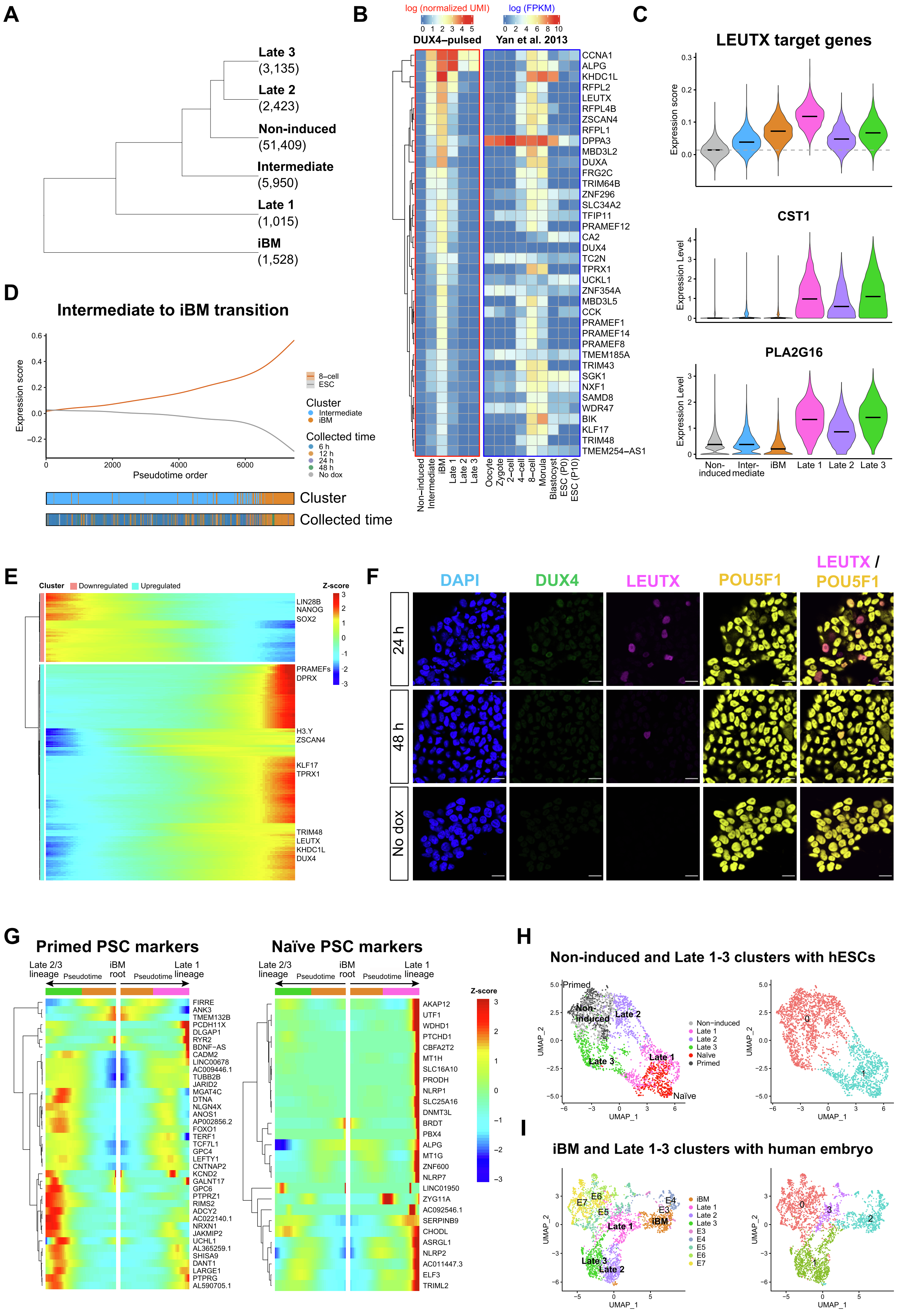
A
Late 3 (3,135)
Late 2 (2,423)
Non-induced (51,409)
Intermediate (5,950)
Late 1 (1,015)
iBM (1,528)
B
log (normalized UMI)
log (FPKM)
DUX4–pulsed
Yan et al. 2013
CCNA1
ALPG
KHDC1L
RFPL2
LEUTX
RFPL4B
ZSCAN4
RFPL1
DPPA3
MBD3L2
DUXA
FRG2C
TRIM64B
ZNF296
SLC34A2
TFIP11
PRAMEF12
CA2
DUX4
TC2N
TPRX1
UCKL1
ZNF354A
MBD3L5
CCK
PRAMEF1
PRAMEF14
PRAMEF8
TMEM185A
TRIM43
SGK1
NXF1
SAMD8
WDR47
BIK
KLF17
TRIM48
TMEM254–AS1
Non–induced
Intermediate
iBM
Late 1
Late 2
Late 3
Oocyte
Zygote
2–cell
4–cell
8–cell
Morula
Blastocyst
ESC (P0)
ESC (P10)
C
LEUTX target genes
Expression score
CST1
Expression Level
PLA2G16
Non-induced
Inter-mediate
iBM
Late 1
Late 2
Late 3
D
Intermediate to iBM transition
Expression score
Pseudotime order
8–cell
ESC
Cluster
Intermediate
iBM
Collected time
6 h
12 h
24 h
48 h
No dox
Cluster
Collected time
E
Cluster
Downregulated
Upregulated
Z-score
LIN28B
NANOG
SOX2
PRAMEFs
DPRX
H3.Y
ZSCAN4
KLF17
TPRX1
TRIM48
LEUTX
KHDC1L
DUX4
F
DAPI
DUX4
LEUTX
POU5F1
LEUTX / POU5F1
24 h
48 h
No dox
G
Primed PSC markers
Naïve PSC markers
Late 2/3 lineage
Pseudotime
iBM root
Late 1 lineage
Z-score
FIRRE
ANK3
TMEM132B
PCDH11X
DLGAP1
RYR2
BDNF–AS
CADM2
LINC00678
AC009446.1
TUBB2B
JARID2
MGAT4C
DTNA
NLGN4X
ANOS1
AP002856.2
FOXO1
TERF1
TCF7L1
GPC4
LEFTY1
CNTNAP2
KCND2
GALNT17
GPC6
PTPRZ1
RIMS2
ADCY2
AC022140.1
NRXN1
JAKMIP2
UCHL1
AL365259.1
SHISA9
DANT1
LARGE1
PTPRG
AL590705.1
AKAP12
UTF1
WDHD1
PTCHD1
CBFA2T2
MT1H
SLC16A10
PRODH
NLRP1
SLC25A16
DNMT3L
BRDT
PBX4
ALPG
MT1G
ZNF600
NLRP7
LINC01950
ZYG11A
AC092546.1
SERPINB9
CHODL
ASRGL1
NLRP2
AC011447.3
ELF3
TRIML2
H
Non-induced and Late 1-3 clusters with hESCs
Primed
Non-induced
Late 2
Late 3
Late 1
Naïve
Non-induced
Late 1
Late 2
Late 3
Naïve
Primed
UMAP_1
UMAP_2
I
iBM and Late 1-3 clusters with human embryo
E7
E6
E5
E4
E3
Late 1
iBM
Late 3
Late 2
iBM
Late 1
Late 2
Late 3
E3
E4
E5
E6
E7
UMAP_1
UMAP_2

Figure S3. Detailed characterization of *DUX4*-pulsed hESCs at single-cell level, related to Figure 3

(A) Hierarchical clustering analysis of the 6 clusters. Numbers in parentheses indicate the number of cells.

(B) iBM cluster-marker gene expression in *DUX4*-pulsed hESCs in each cluster (left) and human preimplantation embryo (right). Clustering of genes was performed based on the expression pattern across clusters in *DUX4*-pulsed hESCs. See also Table S3.

(C) LEUTX target gene expression in each cluster. LEUTX target gene expression score was calculated with the 299 genes. The horizontal gray dotted line indicates the median score in the non-induced cluster. *CST1* and *PLA2G16* are representatives. See also Table S1.

(D) Gene expression score changes of eight-cell and ESC in cells from the intermediate and the iBM clusters along the pseudotime.

(E) Heatmap of 675 significantly changed genes ($q < 1e\text{-}100$) along the pseudotime from intermediate to iBM transition, clustered by pseudotemporal expression pattern. x-axis corresponds to the pseudotime order shown in Figure S3D. See also Table S4.

(F) Immunocytochemical detection of DUX4, LEUTX, and POU5F1 in *DUX4*-pulsed hESCs at 24 h, 48 h, and without induction (No dox). DAPI (blue) was used as nuclear counterstain. Scale bars, 20 µm.

(G) Expression changes of primed (left) and naïve (right) PSC markers along the pseudotime from iBM to late transition.

(H) Integration of non-induced and late 1–3 cluster cells with naïve and primed hESCs (Messmer et al., 2019) projected onto the UMAP plot. Non-induced and late 1–3 cluster cells were downsampled to 400 cells per cluster. Colored by original cell identity (left) and cluster annotation (right).

(I) Integration of iBM and late 1–3 cluster cells with the human embryo (Petropoulos et al., 2016) projected onto the UMAP plot. iBM and late 1–3 cluster cells were downsampled to 400 cells per cluster. Colored by original cell identity (left) and cluster annotation (right).

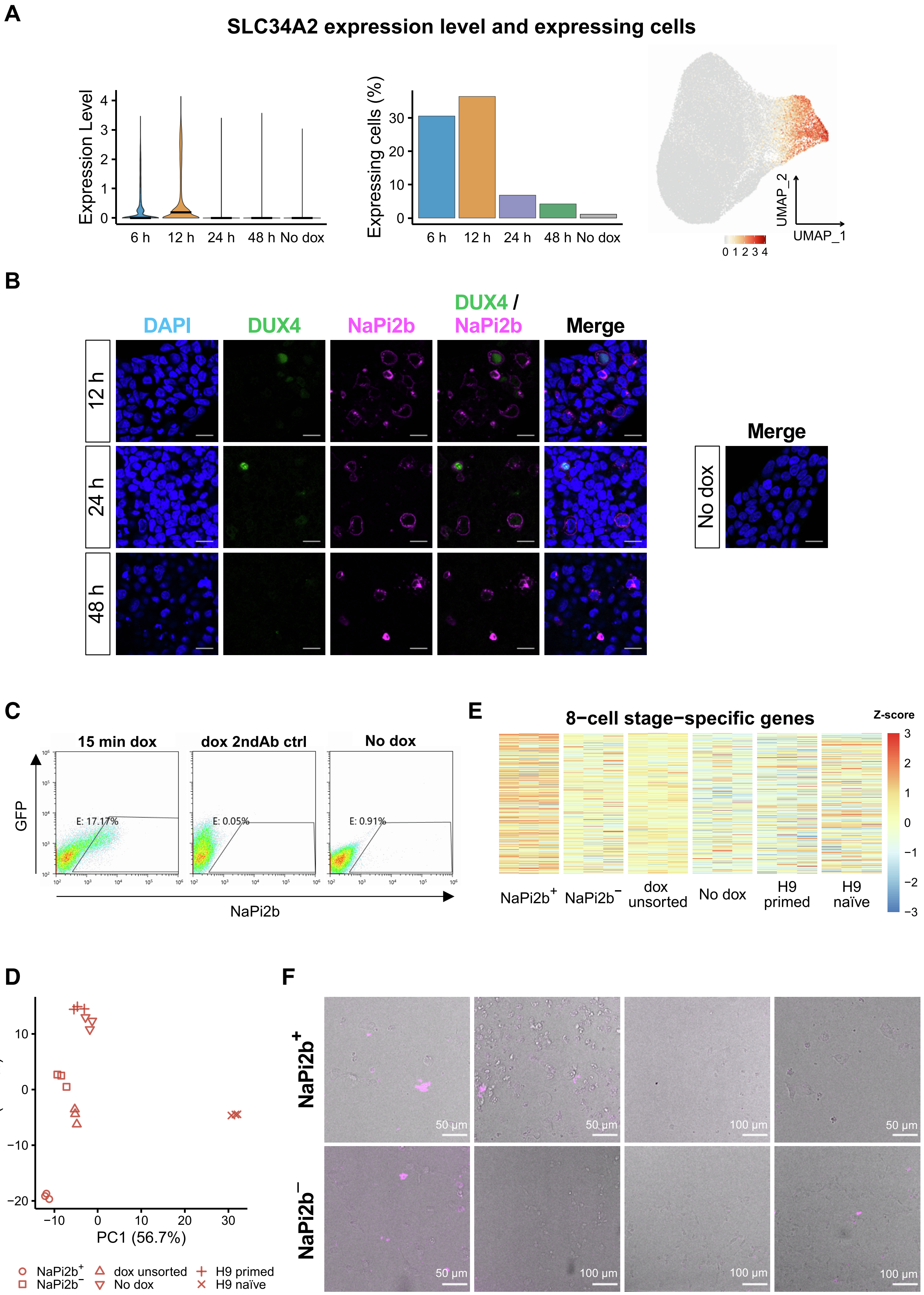
A
SLC34A2 expression level and expressing cells
Expression Level
6 h
12 h
24 h
48 h
No dox
Expressing cells (%)
UMAP_2
UMAP_1
0 1 2 3 4
B
DAPI
DUX4
NaPi2b
DUX4 / NaPi2b
Merge
12 h
24 h
48 h
No dox
C
15 min dox
dox 2ndAb ctrl
No dox
GFP
NaPi2b
E: 17.17%
E: 0.05%
E: 0.91%
E
8−cell stage−specific genes
Z-score
NaPi2b+
NaPi2b−
dox unsorted
No dox
H9 primed
H9 naïve
D
PC2 (35.2%)
PC1 (56.7%)
NaPi2b+
NaPi2b−
dox unsorted
No dox
H9 primed
H9 naïve
F
NaPi2b+
NaPi2b−
50 µm
100 µm

Figure S4. iBM cells could be enriched with an anti-NaPi2b antibody, related to Figure 4

(A) Left: *SLC34A2* expression levels shown as log normalized UMI counts. Middle: proportion of *SLC34A2* expressing cells (UMI count > 1). Right: *SLC34A2* expression projected onto the UMAP plot.

(B) Immunocytochemical detection of DUX4 and NaPi2b in *DUX4*-pulsed hESCs at 12 h, 24 h, and 48 h. Untreated cells (No dox) are shown in right. DAPI (blue) was used as nuclear counterstain. Scale bars, 20 μm.

(C) Flow cytometric analysis showing the gating of NaPi2b-positive cells. Representative data from two independent experiments are shown. 2ndAb ctrl, secondary antibody control.

(D) Principal component analysis of the sorted NaPi2b$^+$ and NaPi2b$^-$ cells, unsorted *DUX4*-pulsed hESCs (dox unsorted), no induction (No dox), and H9 primed and naïve ESCs (n = 3 independent experiments).

(E) Heatmap showing the expression of eight-cell stage-specific genes (990 genes).

(F) Annexin V staining of NaPi2b$^+$ and NaPi2b$^-$ cells after 6 h of culture. Scale bars are as shown in the images.

KEY RESOURCES TABLE

REAGENT or RESOURCE	SOURCE	IDENTIFIER
Antibodies		
Mouse monoclonal anti-DUX4	Merck Millipore	Cat# MABD116; clone 9A12
Rabbit monoclonal anti-DUX4	Abcam	Cat# ab124699; RRID: AB_10973363; clone E5-5
Goat polyclonal anti-OCT3/4 (N-19)	Santa Cruz Biotechnology	Cat# sc-8628; RRID: AB_653551
Rabbit polyclonal anti-KLF17	Atlas Antibodies	Cat# HPA024629; RRID: AB_1668927
Rabbit polyclonal anti-LEUTX	Novus Biologicals	Cat# NBP1-90890; RRID: AB_11053314
Rabbit polyclonal anti-Cleaved Caspase-3 (Asp175)	Cell Signaling Technology	Cat# 9661; RRID: AB_2341188
Mouse monoclonal anti-NaPi2b (MX35)	Memorial Sloan Kettering	
Donkey Anti-Mouse IgG (H+L) (Alexa Fluor® 647)	Thermo Fisher Scientific	Cat# A31571; RRID: AB_162542
Donkey Anti-Rabbit IgG (H+L) (Alexa Fluor® 488)	Thermo Fisher Scientific	Cat# A21206; RRID: AB_2535792
Donkey Anti-Goat IgG (H+L) (Alexa Fluor® 594)	Thermo Fisher Scientific	Cat# A11058; RRID: AB_2534105
Donkey Anti-Mouse IgG (H+L) (Alexa Fluor® 488)	Thermo Fisher Scientific	Cat# A21202; RRID: AB_141607
Donkey Anti-Rabbit IgG (H+L) (Alexa Fluor® 594)	Thermo Fisher Scientific	Cat# A21207; RRID: AB_141637
Donkey Anti-Rabbit IgG HRP Conjugate	Jackson ImmunoResearch	Cat# 711-035-152; RRID: AB_10015282
Annexin V-Cy5 Apoptosis Detection Kit	Abcam	Cat# ab14150
Chemicals, peptides, and recombinant proteins		
Geltrex	Thermo Fisher Scientific	Cat# A1413302
Essential 8 culture medium	Thermo Fisher Scientific	Cat# A1517001
NaïveCult Expansion Medium	Stemcell Technologies	Cat# 05590
EDTA	Thermo Fisher Scientific	Cat# 15575-020
MMLV-RTase	Promega	Cat# M1701
5× HOT FIREPol qPCR Mix	Solis BioDyne	Cat# 08-25-00020
TrypLE Express Enzyme	Thermo Fisher Scientific	Cat# 12604-021
Ultravision protein Block solution	Thermo Fisher Scientific	Cat# TA-060-PBQ
ROCK inhibitor: Y-27632	Selleckchem	Cat# S1049
DPBS, no calcium, no magnesium	Thermo Fisher Scientific	Cat# 14200
Tween20	Fisher Scientific	Cat# BP337-100
Triton X-100	Fisher Scientific	Cat# BP151-100

4× Laemmli Sample Buffer	Bio-Rad	Cat# 161-0747
Clarity Western ECL Substrate	Bio-Rad	Cat# 170-5061
Critical commercial assays		
NucleoSpin RNA kit	Macherey Nagel	Cat# 740955
Dead Cell Removal Binding Kit	Miltenyi Biotec	Cat# 130-090-101
MS Columns	Miltenyi Biotec	Cat# 130-042-201
Chromium Next GEM Single Cell 3' Kit v3.1	10x Genomics	
Deposited data		
STRT RNA-seq data of *DUX4*-pulsed hESCs	This paper	E-MTAB-10569
STRT RNA-seq data of continuously *DUX4*-induced hESCs	(Vuoristo et al., 2022)	GEO: GSE171803
STRT RNA-seq data of early human embryos	(Töhönen et al., 2015)	ENA PRJEB8994
scRNA-seq data of *DUX4*-pulsed hESCs	This paper	E-MTAB-10581
scRNA-seq data of early human embryos and hESCs	(Yan et al., 2013)	GEO: GSE36552
STRT RNA-seq data of human eight-cell stage cells and hESCs	(Jouhilahti et al., 2016)	ENA PRJEB12467
STRT RNA-seq data of *LEUTX*-inducible hESCs	Gawriyski et al., unpublished	E-MTAB-10539
scRNA-seq data of human preimplantation embryos	(Petropoulos et al., 2016)	E-MTAB-3929
scRNA-seq data of naïve and primed hESCs	(Messmer et al., 2019)	E-MTAB-6819
Experimental models: Cell lines		
Human: DUX4-TetOn hESCs	(Vuoristo et al., 2022)	N/A
H9	WiCell	WA09
Oligonucleotides		
Primer: *LEUTX* Forward: GCTACAATGGGGAAACTGGC	(Jouhilahti et al., 2016)	N/A
Primer: *LEUTX* Reverse: CTCTTCCATTTGGCACGCTG	(Jouhilahti et al., 2016)	N/A
Primer: *ZSCAN4* Forward: CCTCCCAGACTTCCCAAGAT	(Vuoristo et al., 2022)	N/A
Primer: *ZSCAN4* Reverse: TGTTCCAGCCATCTTGTTCA	(Vuoristo et al., 2022)	N/A
Primer: *TRIM48* Forward: CATCACTGGACTGAGGGACA	(Vuoristo et al., 2022)	N/A
Primer: *TRIM48* Reverse: TGACTGTTGGCTTCATTGTGA	(Vuoristo et al., 2022)	N/A
Primer: cyclophilin G (*PPIG*) Forward: TCTTGTCAATGGCCAACAGA	(Weltner et al., 2018)	N/A
Primer: cyclophilin G (*PPIG*) Reverse: GCCCATCTAAATGAGGAGTT	(Weltner et al., 2018)	N/A
Software and algorithms		
STRT2 pipeline b3e589c	(Ezer et al., 2021)	https://github.com/my0916/STRT2
Picard v2.20.4	https://github.com/broadinstitute/picard	http://broadinstitute.github.io/picard/
HISAT2 v2.1.0	(Kim et al., 2019)	https://daehwankimlab.github.io/hisat2/
featureCounts v1.5.2	(Liao et al., 2014)	http://subread.sourceforge.net/
StringTie v1.3.3	(Pertea et al., 2015)	https://ccb.jhu.edu/software/stringtie/
TEtranscripts v2.2.1	(Jin et al., 2015)	https://github.com/mhammell-laboratory/TEtranscripts

R v4.0.0	(R Development Core Team, 2020)	https://www.r-project.org/
DESeq2 v1.30.0	(Love et al., 2014)	http://www.bioconductor.org/packages/release/bioc/html/DESeq2.html
Cell Ranger v3.1.0	10x Genomics	https://support.10xgenomics.com/single-cell-gene-expression/software/pipelines/latest/what-is-cell-ranger
STAR aligner	(Dobin et al., 2013)	https://github.com/alexdobin/STAR
Seurat v4.0.0	(Hao et al., 2021)	https://satijalab.org/seurat/
SingleR v1.4.1	(Aran et al., 2019)	https://bioconductor.org/packages/release/bioc/html/SingleR.html
Monocle v2.18.0	(Qiu et al., 2017)	http://cole-trapnell-lab.github.io/monocle-release/
STRTprep	(Krjutškov et al., 2016)	https://github.com/shka/STRTprep
ImageLab	Bio-Rad	http://www.bio-rad.com/en-ch/product/image-lab-software?ID=KRE6P5E8Z
Fiji	(Schindelin et al., 2012)	https://fiji.sc/

Supplemental Tables in Excel files

Table S1. List of genes used in this study, related to Figures 1–3.

Table S2. Quality metrics of the scRNA-seq data in each experiment, related to Figures 2 and 3.

Table S3. List of marker genes in each cluster, related to Figure 3.

Table S4. List of differentially expressed genes along the pseudotime (q < 1e-100) from intermediate to iBM transition, related to Figure 3.

Supplemental experimental procedures

Western blotting

Cells were washed with PBS and lysed with RIPA buffer (Thermo Scientific). Lysate was centrifuged at 14,000 rcf for 15 min and supernatant was collected to a new tube. Protein samples were prepared using 4× Laemmli Sample Buffer (Bio-Rad) with 10% beta mercaptoethanol and boiled at 100°C for 5 min. Samples were loaded on Mini-PROTEAN TGX Stain-Free Gels (Bio-Rad) and run with 1× Tris-Glycine-SDS (Bio-Rad) buffer in Mini-PROTEAN Tetra Vertical Electrophoresis Cell (Bio-Rad). Proteins were transferred using the Trans Blot Turbo device (Bio-Rad) and Trans-Blot Turbo Transfer Pack (Bio-Rad). Membranes were quickly soaked first in dH_2O and then in 70% EtOH and blocked in 5% skim milk diluted in 1× Tris Buffered Saline with 0.05% Tween 20 (TBST) for 1 h. After blocking, membrane was washed three times with TBST and incubated with DUX4 antibody (diluted 1:1000 in 3% Skim Milk-TBST) overnight at 4°C. The next day, the membrane was washed three times with TBST on shaker for 10 min and incubated with HRP-conjugated anti-rabbit IgG (diluted 1:40000 in 3% Skim Milk-TBST) at room temperature for 1 h. The membrane was washed three times with TBST on shaker for 10 min and developed in Clarity Western ECL Substrate (Bio-Rad) at room temperature for 5 min. Membrane was imaged with ChmeiDoc MP Imaging System. Image was analyzed with ImageLab Software (Bio-Rad) by normalizing DUX4 to total protein.

Annexin V staining

The cells were washed with Annexin V binding buffer and incubated in Annexin V solution (1:100 in binding buffer) at room temperature in dark, for 5 min. The cells were washed with Annexin V Binding buffer, fixed with 2% paraformaldehyde at room temperature in dark, for 10 min. The cells were washed twice with PBS and imaged.

STRT RNA-seq data processing

The sequenced STRT RNA-seq raw reads were processed as described elsewhere (https://github.com/my0916/STRT2) (Ezer et al., 2021). Briefly, raw base call (BCL) files were demultiplexed and converted to FASTQ files with Picard tools (v2.20.4; http://broadinstitute.github.io/picard/), and aligned to the human reference genome hg19, human ribosomal DNA unit (GenBank: U13369), and ERCC spike-ins (SRM 2374) with the GENCODE (v28) transcript annotation by HISAT2 (v2.1.0) (Kim et al., 2019). Potential PCR duplicates were flagged with Picard MarkDuplicates. For gene-based analysis, uniquely mapped reads within the 5'-UTR or 500 bp upstream of the protein-coding genes were counted using Subread featureCounts (v1.5.2) (Liao et al., 2014) with '−−ignoreDup' option. The mapped reads were further assembled by StringTie (v1.3.3) (Pertea et al., 2015) and those reads within the first exons of the assembled transcripts (TFEs) were counted as previously described (Töhönen et al., 2015). Two samples collected immediately after induction were

excluded due to a low number of mapped reads. PCA was performed using the top 500 most variable genes. The STRT RNA-seq data of continuous *DUX4* induction, treated by doxycycline for 4 h, was obtained from Vuoristo et al. (Vuoristo et al., 2022) and reprocessed as described above. The expression of transposable elements was quantified using TEtranscripts (v2.2.1) with 'uniq' mode (Jin et al., 2015). Differential expression analysis between doxycycline-induced and non-induced cells was performed with the R (v4.0.0) package DESeq2 (v1.30.0) (Love et al., 2014), and the expression values were normalized by the library size calculated with DESeq2. Genes or transposable elements with Benjamini–Hochberg-adjusted *P*-value < 0.05 were considered statistically significant. The correlation coefficients (r) and *P*-values were calculated using a two-sided Spearman's correlation test. The list of EGA genes was retrieved from Töhönen et al. (Töhönen et al., 2015) (**Table S1**), and TFEs overlapped with these gene regions were analyzed further. The list of DUX4 target genes expressed in the cleavage-stage human embryo was retrieved from Resnick et al. (Resnick et al., 2019) (**Table S1**). The list of stage-specific genes was retrieved from Stirparo et al. (Stirparo et al., 2018). The STRT RNA-seq data of HS980 primed ESCs and eight-cell stage cells was obtained from Jouhilahti et al. (Jouhilahti et al., 2016). For the integration of the two STRT RNA-seq datasets, log-normalized expression values were quantile normalized and subtracted by the mean of each gene across the samples in each dataset (Liu et al., 2017). The multidimensional scaling analysis was performed using the cmdscale function based on the Spearman correlation distance matrix between the samples of the two datasets.

scRNA-seq data processing

Data pre-processing and cell clustering

The raw BCL files were demultiplexed and converted to FASTQ files with Cell Ranger (10x Genomics, v3.1.0) mkfastq, and mapped against the customized human reference genome (GRCh38 with DUX4-IRES-EmGFP) with STAR (Dobin et al., 2013). The cellranger aggr pipeline was used to combine all the data to generate a gene-count matrix. The output count data were subsequently analyzed with the R package Seurat (v4.0.0) (Hao et al., 2021). Cells with 15,000–100,000 UMI counts, expressing over 3,500 genes and less than 15% mitochondrial counts were kept, resulting in 65,460 cells in total. These data were then processed using the NormalizeData and FindVariableFeatures (using 2,000 features) functions. Next, cell-cycle scores were calculated using the CellCycleScoring function, and data scaling was performed with the ScaleData function, regressing out the S and G2M scores. Principal component analysis (PCA) was performed on the scaled data using the RunPCA function, and cell clustering was performed using the FindNeighbours (using the top 10 PCs) and FindClusters (resolution = 0.6) functions. UMAP was implemented on the top 10 PCs with the RunUMAP function. Here, 10 clusters with lower UMAP_1 values (left clusters) showing similar expression profiles were mainly composed of no-dox cells and were assigned as the 'non-induced' cluster. The dendrogram was generated with the BuildClusterTree function. To measure the expression of *DUX4*, we quantified the expression of DUX4-IRES-EmGFP to avoid problems of mapping to the D4Z4 repeat locus. Average expression level in each

cluster was calculated with the AverageExpression function. The iBM cluster specific markers shown in Figure S3B were identified by the FindAllMarkers function and selected as pct.1 > 0.8, pct.2 < 0.5, and avg_logFC > 1.

Gene expression scoring and cell type annotation

Gene expression scores of each signature were calculated using the gene signature scoring function retrieved from Liu et al. (Liu et al., 2020). Briefly, the average expression values of the genes of interest were subtracted by the aggregated expression values of a set of randomly selected control genes at similar expression level. The list of EGA genes were obtained from Töhönen et al. (Töhönen et al., 2015), and that of signature genes of primed and naïve PSCs were obtained from Liu et al. (Liu et al., 2020). The list of eight-cell and ESC genes were retrieved from Jouhilahti et al. (Jouhilahti et al., 2016), where the top 121 and 119 differentially expressed genes based on the differential expression score by STRTprep (Krjutškov et al., 2016) were selected, respectively (**Table S1**). The list of 299 LEUTX-target genes were retrieved from the significantly upregulated genes in our unpublished STRT RNA-seq data on *LEUTX*-inducible hESCs (Gawriyski et al., unpublished) (**Table S1**). Cell type annotation was conducted with the R package SingleR (v1.4.1) (Aran et al., 2019), using the scRNA-seq data of human preimplantation embryos and ESCs (Yan et al., 2013) as the reference data.

Pseudotime trajectory analysis

Pseudotime trajectory analysis was performed using the R package Monocle (v2.18.0) (Qiu et al., 2017) for two groups of clusters: i) intermediate and iBM clusters, ii) iBM and late 1–3 clusters, respectively. Cluster marker genes (**Table S3**) identified by the FindAllMarkers function in Seurat were used for ordering the cells, and dimensionality was reduced using the DDRTree algorithm. A generalized additive model (GAM) was fitted to the scaled expression values calculated by Seurat and the pseudotime order of cells using the geom_smooth function of the R package ggplot2 (v3.3.3). Heatmaps were generated with the Monocle plot_pseudotime_heatmap and plot_genes_branched_heatmap functions.

Integration of scRNA-seq datasets

scRNA-seq data of human preimplantation embryos (Petropoulos et al., 2016) and naïve and primed hESCs (Messmer et al., 2019) were obtained from the ArrayExpress database with the accession number E-MTAB-3929 and E-MTAB-6819, respectively. These data were processed and integrated with our scRNA-seq dataset of *DUX4*-pulsed hESCs using the FindIntegrationAnchors and IntegrateData functions in Seurat with dimensionality of 30. Our cells were randomly downsampled to 400 cells per cluster so that the number of cells was comparable between different datasets (1,529 human embryonic cells and 836 hESCs). Identical cells from each cluster were used for the integration with different datasets.

Supplemental References

Aran, D., Looney, A.P., Liu, L., Wu, E., Fong, V., Hsu, A., Chak, S., Naikawadi, R.P., Wolters, P.J., Abate, A.R., *et al.* (2019). Reference-based analysis of lung single-cell sequencing reveals a transitional profibrotic macrophage. Nat Immunol *20*, 163-172.

Dobin, A., Davis, C.A., Schlesinger, F., Drenkow, J., Zaleski, C., Jha, S., Batut, P., Chaisson, M., and Gingeras, T.R. (2013). STAR: ultrafast universal RNA-seq aligner. Bioinformatics *29*, 15-21.

Ezer, S., Yoshihara, M., Katayama, S., Daub, C., Lohi, H., Krjutskov, K., and Kere, J. (2021). Generation of RNA sequencing libraries for transcriptome analysis of globin-rich tissues of the domestic dog. STAR Protoc *2*, 100995.

Hao, Y., Hao, S., Andersen-Nissen, E., Mauck, W.M., 3rd, Zheng, S., Butler, A., Lee, M.J., Wilk, A.J., Darby, C., Zager, M., *et al.* (2021). Integrated analysis of multimodal single-cell data. Cell *184*, 3573-3587.e3529.

Jin, Y., Tam, O.H., Paniagua, E., and Hammell, M. (2015). TEtranscripts: a package for including transposable elements in differential expression analysis of RNA-seq datasets. Bioinformatics *31*, 3593-3599.

Jouhilahti, E.M., Madissoon, E., Vesterlund, L., Töhönen, V., Krjutškov, K., Plaza Reyes, A., Petropoulos, S., Månsson, R., Linnarsson, S., Bürglin, T., *et al.* (2016). The human PRD-like homeobox gene LEUTX has a central role in embryo genome activation. Development *143*, 3459-3469.

Kim, D., Paggi, J.M., Park, C., Bennett, C., and Salzberg, S.L. (2019). Graph-based genome alignment and genotyping with HISAT2 and HISAT-genotype. Nat Biotechnol *37*, 907-915.

Krjutškov, K., Katayama, S., Saare, M., Vera-Rodriguez, M., Lubenets, D., Samuel, K., Laisk-Podar, T., Teder, H., Einarsdottir, E., Salumets, A., *et al.* (2016). Single-cell transcriptome analysis of endometrial tissue. Hum Reprod *31*, 844-853.

Liao, Y., Smyth, G.K., and Shi, W. (2014). featureCounts: an efficient general purpose program for assigning sequence reads to genomic features. Bioinformatics *30*, 923-930.

Liu, X., Nefzger, C.M., Rossello, F.J., Chen, J., Knaupp, A.S., Firas, J., Ford, E., Pflueger, J., Paynter, J.M., Chy, H.S., *et al.* (2017). Comprehensive characterization of distinct states of human naive pluripotency generated by reprogramming. Nat Methods *14*, 1055-1062.

Liu, X., Ouyang, J.F., Rossello, F.J., Tan, J.P., Davidson, K.C., Valdes, D.S., Schröder, J., Sun, Y.B.Y., Chen, J., Knaupp, A.S., *et al.* (2020). Reprogramming roadmap reveals route to human induced trophoblast stem cells. Nature *586*, 101-107.

Love, M.I., Huber, W., and Anders, S. (2014). Moderated estimation of fold change and dispersion for RNA-seq data with DESeq2. Genome Biol *15*, 550.

Messmer, T., von Meyenn, F., Savino, A., Santos, F., Mohammed, H., Lun, A.T.L., Marioni, J.C., and Reik, W. (2019). Transcriptional Heterogeneity in Naive and Primed Human Pluripotent Stem Cells at Single-Cell Resolution. Cell Rep *26*, 815-824.e814.

Pertea, M., Pertea, G.M., Antonescu, C.M., Chang, T.C., Mendell, J.T., and Salzberg, S.L. (2015). StringTie enables improved reconstruction of a transcriptome from RNA-seq reads. Nat Biotechnol *33*, 290-295.

Petropoulos, S., Edsgärd, D., Reinius, B., Deng, Q., Panula, S.P., Codeluppi, S., Plaza Reyes, A., Linnarsson, S., Sandberg, R., and Lanner, F. (2016). Single-Cell RNA-Seq Reveals Lineage and X Chromosome Dynamics in Human Preimplantation Embryos. Cell *165*, 1012-1026.

Qiu, X., Mao, Q., Tang, Y., Wang, L., Chawla, R., Pliner, H.A., and Trapnell, C. (2017). Reversed graph embedding resolves complex single-cell trajectories. Nat Methods *14*, 979-982.

R Development Core Team (2020). R: A language and environment for statistical computing. (R Foundation for Statistical Computing, Vienna, Austria).

Resnick, R., Wong, C.J., Hamm, D.C., Bennett, S.R., Skene, P.J., Hake, S.B., Henikoff, S., van der Maarel, S.M., and Tapscott, S.J. (2019). DUX4-Induced Histone Variants H3.X and H3.Y Mark DUX4 Target Genes for Expression. Cell Rep *29*, 1812-1820.e1815.

Stirparo, G.G., Boroviak, T., Guo, G., Nichols, J., Smith, A., and Bertone, P. (2018). Integrated analysis of single-cell embryo data yields a unified transcriptome signature for the human pre-implantation epiblast. Development *145,* dev158501.

Töhönen, V., Katayama, S., Vesterlund, L., Jouhilahti, E.M., Sheikhi, M., Madissoon, E., Filippini-Cattaneo, G., Jaconi, M., Johnsson, A., Bürglin, T.R.*, et al.* (2015). Novel PRD-like homeodomain transcription factors and retrotransposon elements in early human development. Nat Commun *6*, 8207.

Vuoristo, S., Bhagat, S., Hydén-Granskog, C., Yoshihara, M., Gawriyski, L., Jouhilahti, E.M., Ranga, V., Tamirat, M., Huhtala, M., Kirjanov, I.*, et al.* (2022). DUX4 is a multifunctional factor priming human embryonic genome activation. iScience *25*, 104137.

Weltner, J., Balboa, D., Katayama, S., Bespalov, M., Krjutskov, K., Jouhilahti, E.M., Trokovic, R., Kere, J., and Otonkoski, T. (2018). Human pluripotent reprogramming with CRISPR activators. Nat Commun *9*, 2643.

Yan, L., Yang, M., Guo, H., Yang, L., Wu, J., Li, R., Liu, P., Lian, Y., Zheng, X., Yan, J.*, et al.* (2013). Single-cell RNA-Seq profiling of human preimplantation embryos and embryonic stem cells. Nat Struct Mol Biol *20*, 1131-1139.
